# Synthesis, anticancer evaluation, molecular docking and ADME study of novel pyrido[4ʹ,3ʹ:3,4]pyrazolo[1,5-*a*]pyrimidines as potential tropomyosin receptor kinase A (TrKA) inhibitors

**DOI:** 10.1186/s13065-024-01166-7

**Published:** 2024-04-06

**Authors:** Nadia Hanafy Metwally, Emad Abdullah Deeb, Ibrahim Walid Hasani

**Affiliations:** Chemistry Department, Faculty of Science, Cairo University, Giza, 12613 Egypt

**Keywords:** Pyrido[4ʹ,3ʹ:3,4]pyrazolo[1,5-*a*]pyrimidines, Anticancer activity, TrKA enzyme, Apoptotic activity, Molecular docking, ADME studies

## Abstract

The starting compound 3-amino-1,7-dihydro-4*H*-pyrazolo[4,3-*c*]pyridine-4,6(5*H*)-dione** (1)** is reacted with each of diketone and *β*-ketoester, forming pyridopyrazolo[1,5-*a*]pyrimidines **4a**,**b** and **14a**,**b**, respectively. The compounds **4** and **14** reacted with each of aromatic aldehyde and arenediazonium salt to give the respective arylidenes and arylhydrazo derivatives, respectively. The structure of the new products was established using spectroscopic techniques. The cytotoxic activity of selected targets was tested in *vitro* against three cancer cell lines **MCF7**, **HepG2** and **HCT116**. The data obtained from enzymatic assays of TrKA indicated that compounds **7b** and **16c** have the strongest inhibitory effects on TrKA with IC_50_ = 0.064 ± 0.0037 μg/ml and IC_50_ = 0.047 ± 0.0027 μg/ml, respectively, compared to the standard drug Larotrectinib with IC_50_ = 0.034 ± 0.0021 μg/ml for the **HepG2** cancer cell line. In cell cycle analysis, compounds **7b**, **15b**, **16a** and **16c** caused the greatest arrest in cell cycle at the G2/M phase. In addition, compound **15b** has a higher apoptosis-inducing effect (36.72%) than compounds **7b** (34.70%), **16a** (21.14) and **16c** (26.54%). Compounds **7b**, **16a** and **16c** were shown fit tightly into the active site of the TrKA kinase crystal structure (PDB: 5H3Q). Also, ADME study was performed on some selected potent anticancer compounds described in this study.

**Supplementary Information:**

The online version contains supplementary material available at 10.1186/s13065-024-01166-7.

## Highlights

- A series of new pyrido[4ʹ,3ʹ:3,4]pyrazolo[1,5-*a*]pyrimidine derivatives were synthesized.
- The anticancer activity of the new compounds were tested in vitro.
- Compounds **7b** and **16c** showed broad spectrum potent anticancer activity.
- Compound **15b** induced cell cycle arrest at G2/M phase in HepG-2 cell line.
- Compound **7c**, the most promising agent, can be absorbed very easily by the gastrointestinal tract with potential BBB permeability.

**Supplementary Information:**

The online version contains supplementary material available at 10.1186/s13065-024-01166-7.

## Introduction

Cancer is the growth of cells in certain parts of the body that grow out of control and can invade other tissues. Cancer is the second leading cause of death worldwide and chemotherapy, radiotherapy, and/or surgery are the most common cancer treatment techniques. Over the past decade, much research has focused on finding new therapies that reduce the side effects of conventional treatments.

**Fig. 1:**
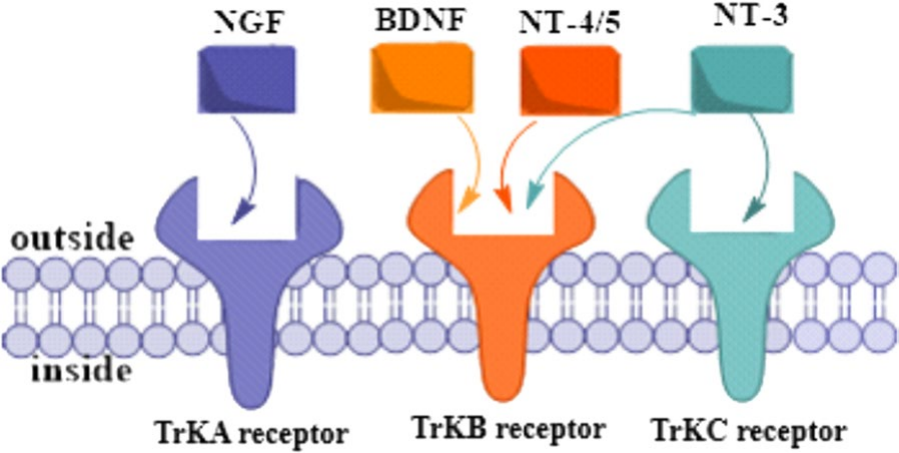
The three types of tropomyosin receptor kinases (Trks)

The identification of gene fusions in certain cancers has provided a practical target for expanding therapeutic options and advancing precision medicine. These genetic abnormalities lead to the expression of constitutively active fusion proteins that are carcinogenic drivers [1]. Gene fusions are a type of mutation that commonly occurs in many types of cancer. They often result from chromosomal rearrangement that cause migration of coding or regulatory regions between genes. The tropomyosin tyrosine receptor kinase (TrK) family is of interest because the NTRK genes encoding have been implicated in gene fusions identified in a variety of adult and pediatric tumors. Three members of TrKA, encode transmembrane proteins NTRK1, TrKB (NTRK2) and TrKC (NTRK3) [2, 3]. As shown in Fig. 1, Trks are activated by the a family of nerve growth factors including nerve growth factor (NGF), brain-derived neurotrophic factor (BDNF), Neurotrophin-4 (NT-4) and Neurotrophin-3 (NT-3) [3].

**Fig. 2:**
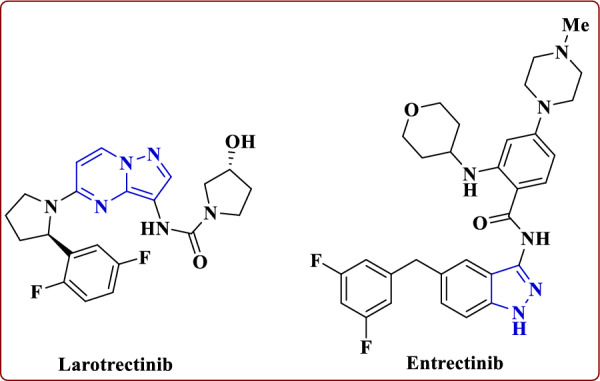
Larotrectinib and entrectinib as type-I multi-target kinase inhibitors

Larotrectinib is an inhibitor of the tropomyosin receptor kinases TrkA, TrkB, and TrkC (approved by the FDA in 2018). It has been indicated in adults and adolescents with solid tumors harboring NTRK gene fusions without a known acquired resistance mutations, in case of metastases or undergoing surgery. Resection can cause serious complications. Figure 2 shows another multitarget type-I kinase inhibitor with a pyrazole ring, such as entrectinib [4–7]. Despite the high response rates achieved with first-generation TrK inhibitors, drug resistance still exists, ultimately leading to treatment failure [8, 9]. Additionally, TrKA is the most commonly identified oncogene, found in several tumor types at a rate of approximately 7.4% (4% for TRKB and 3.4% for TRKA) [10, 11].

Furthermore, TrKA has been shown to mediate the stimulation of early tumor growth [12]. Therefore, inhibiting TrKA signaling is an attractive clinical approach for cancer therapy. Therefore, it is highly desirable to obtain new selective Trk inhibitors with different chemical scaffolds as new anti-neuroblastoma (NB) agents. Previously, two TrKA inhibitors were approved by the U.S. Food and Drug Administration (FDA). Larotrectinib was approved for solid tumors with NTRK gene fusions in November 2018 [13], with very low IC_50_ value for the Trk family (IC_50_ = 2–20 nM), and significant activity outside this kinase family [14]. Entrectinib was approved in August 2019 for NTRK gene fusion-positive or ROS1-positive solid tumors [15]. According to the classification of Shokat et al. [16] all are classified as type I kinase inhibitors.

**Fig. 3:**
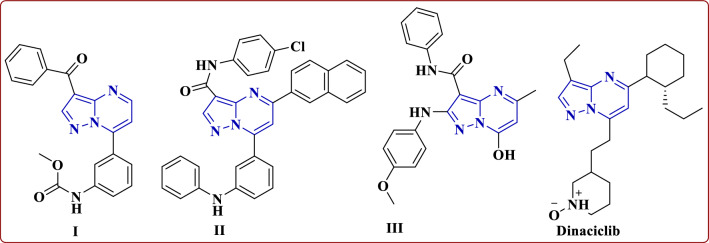
Some pyrazolo[1,5-*a*]pyrimidines such as **I–III** with a standard drug dinaciclib as anticancer agents

Additionally, some pyrazolo[1,5-*a*]pyrimidine derivatives such as **I**, **II** and **III** showed good activity against HCT116, HeLa and HepG2 cell lines, respectively [17–19]. Moreover, the standard drug dinaciclib **IV** acts as a potent and selective cyclin-dependent kinase (CDK) inhibitor (Fig. 3) [20, 21].

**Fig. 4:**
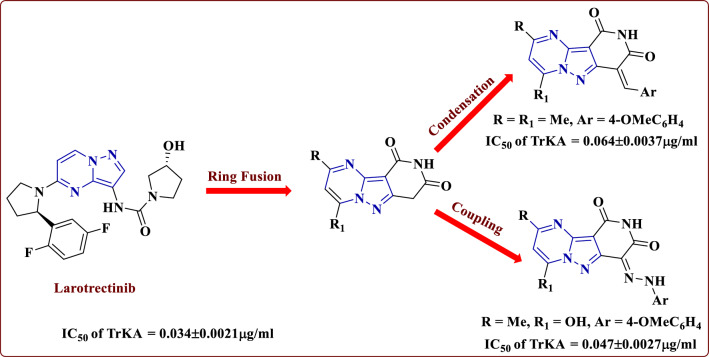
Our target compounds as anticancer agents and TrKA inhibitors compared to larotrectinib

Based on our research program to synthesize several bioactive heterocyclic compounds [22–33], we followed our previous work [24], which showed that some pyrazolopyridine derivatives have good cytotoxic activity against the MCF7 and HepG2 cell lines, respectively. Therefore, we synthesized other series of some novel heterocycles containing pyrazolo[1,5-*a*] pyrimidine hybrid with pyridine moiety to evaluate their anticancer activity against the three cell lines MCF7, HepG2 and HCT116. Moreover, evaluation of these compounds against TrKA enzyme was done (Fig. 4).

## Experimental

### Materials and methods

The melting points are uncorrected and measured on an Electrothermal instrument (9100). Infrared spectra were recorded on a Perkin Elmer 1430 spectrophotometer (KBr pellet). On a Varian Gemini NMR spectrometer using tetramethylsilane as the internal reference and the results are expected as δ value, the ^1^H NMR and.^13^C NMR spectra were recorded at deuterated dimethylsulfoxide at 300 and 75 MHz. Mass spectra were performed on a Shimadzu GCMS-QP 1000 Ex mass spectrometer at 70 eV. Elemental analysis was performed at the Center for Microanalyses of Cairo University, Giza, Egypt. Enzyme, cell cycle and apoptosis inhibition were performed at VACSERA, Cairo, Egypt. Compound **1** was prepared according to the previous literature [34]

### General procedure of synthesis of 4a,b

In 15 ml of DMF, a mixture of compound **1** (0.01 mol) and diketones **2a**,**b** (0.01 mol) containing few drops of piperidine was heated under reflux for 10 h. The resulting solid was filtered, washed with ethanol and recrystallized from DMF.

#### 2,4-Dimethylpyrido[4ʹ,3ʹ:3,4]pyrazolo[1,5-*a*]pyrimidine-8,10(7*H*,9*H*)-diones (4a)

Brown crystals, yield 86%, m.p. 330 °C, νmax/cm^−1^ (KBr) 3187 (NH), 1702 (CO); 1H NMR (DMSO-d6) δ = 2.58 (s, 3H, CH3), 2.70 (s, 3H, CH3), 4.05 (s, 2H, CH2), 7.16 (s, 1H, pyrimidine-H), 10.82 (s, 1H, NH); 13 C NMR (DMSO-d6) δ = 14.55, 16.74, 50.60, 72.32, 79.24, 79.75, 114.41, 117.11, 143.55, 154.16, 167.77; m/z 230 = (M+, 67.6%), 214 (2.78%), 201 (3.19%), 187 (85.1%), 159 (18.79%), 132 (9.1%), 113 (23.6%), 101 (18.87%), 87 (22.2%), 78 (13.4%), 59 (100%), 52 (6.85%); Anal. Calcd for C11H10N4O2: C, 57.39; H, 4.38; N, 24.34. Found: C, 57.53; H, 4.55; N, 24.12%.

#### 2,4-Diphenylpyrido[4ʹ,3ʹ:3,4]pyrazolo[1,5-*a*]pyrimidine-8,10(7*H*,9*H*)-dione (4b)

Brown crystals, yield 76%, m.p. 300 °C, ν_max_/cm^−1^ (KBr) 3181 (NH), 1693 (CO); ^1^H NMR (DMSO-*d*_6_) δ = 4.04 (s, 2H, CH_2_), 7.55–7.59 (m, 7H, Ar–H), 7.61 (s, 1H, Ar), 7.98 (m, 2H, Ar–H), 8.11 (m, 1H, CH), 11.0 (s, 1H, NH); m/z 354 = (M^+^, 100%), 311 (93.1%), 282 (8.1%), 255 (5.2%), 204 (18.8%), 189 (9.3%), 155 (13.3%), 127 (17.5%), 102 (57.1%), 77 (28.2%), 64 (8.0%), 51 (11.4%); Anal. Calcd for C_21_H_14_N_4_O_2_: C, 71.18; H, 3.98; N, 15.81. Found: C, 71.33; H, 3.79; N, 15.57%.

### General procedure of synthesis of 7a–t

A mixture of compounds **4a** (or **4b**) (0.01 mol) and the appropriate aldehyde **6a**–**j** (0.01 mol) in DMF (15 ml) with few drops of piperidine was refluxed for 5 h. The reaction mixture was cooled at room temperature, the solid so formed was collected by filtration and recrystallized from DMF.

#### 7-Benzylidene-2,4-dimethylpyrido[4ʹ,3ʹ:3,4]pyrazolo[1,5-*a*]pyrimidine-8,10(7*H*,9*H*)-dione (7a)

Brown crystals, yield 63%, m.p. 240 °C, ν_max_/cm^−1^ (KBr) 3181 (NH), 1698 (CO); ^1^H NMR (DMSO-*d*_6_) δ = 2.54 (s, 3H, CH_3_), 2.62 (s, 3H, CH_3_), 7.14 (s, 1H, CH), 7.48 (m, 3H, Ar–H), 8.15 (s, 1H, CH), 8.31–8.33 (s, 2H, Ar), 11.0 (s, 1H, NH); ^13^C NMR (DMSO-*d*_6_) δ = 17.02, 24.90, 98.38, 112.48, 118.74, 128.34, 131.67, 132.94, 133.95, 145.40, 146.33, 146.94, 150.60, 159.70, 163.62, 166.00; Anal. Calcd for C_18_H_14_N_4_O_2_: C, 67.92; H, 4.43; N, 17.60. Found: C, 67.75; H, 4.54; N, 17.36%.

#### 7-(4-Methoxyphenyl-2,4-dimethylpyrido[4ʹ,3ʹ:3,4]pyrazolo[1,5-*a*]pyrimidine-8,10-(7*H*,9*H*)-dione (7b)

Brown crystals, yield 70%, m.p. 280 °C, ν_max_/cm^−1^ (KBr) 3211 (NH), 1697 (CO); ^1^H NMR (DMSO-*d*_6_) δ = 2.55 (s, 3H, CH_3_), 2.69 (s, 3H, CH_3_), 3.68 (s, 3H, OCH_3_), 7.0 (d, *J* = 6 Hz, 2H, Ar–H), 7.12 (s, 1H, Ar–H), 8.10 (s, 1H, CH), 8.49 (d, *J* = 6 Hz, 2H, Ar–H), 10.93 (s, 1H, NH); Anal. Calcd for C_19_H_16_N_4_O_3_: C, 65.51; H, 4.63; N, 16.08. Found: C, 65.38; H, 4.74; N, 16.34%.

#### 7-(4-Chlorophenyl-2,4-dimethylpyrido[4ʹ,3ʹ:3,4]pyrazolo[1,5-*a*]pyrimidine-8,10(7*H*,9*H*)-dione (7c)

Yellow crystals, yield 71%, m.p. 260 °C, ν_max_/cm^−1^ (KBr) 3211 (NH), 1697 (CO); ^1^H NMR (DMSO-*d*_6_) δ = 2.46 (s, 3H, CH_3_), 2.71 (s, 3H, CH_3_), 7.18 (s, 1H, CH), 7.46–7.63 (m, 4H, Ar–H). 8.42 (s, 1H, CH), 11.02 (s, 1H, NH); Anal. Calcd for C_18_H_13_ClN_4_O_2_: C, 61.28; H, 3.71; Cl, 10.05; N, 15.88. Found: C, 61.38; H, 3.59; N, 15.65%.

#### 7-(2-Hydroxybenzylidene)-2,4-dimethylpyrido[4ʹ,3ʹ:3,4]pyrazolo[1,5-*a*]pyrimidine-8,10(7*H*,9*H*)-dione (7d)

Brown crystals, yield 89%, m.p > 360 °C, ν_max_/cm^−1^ (KBr) 3417 (OH), 3269 (NH), 1691(CO);^1^H NMR (DMSO-*d*_6_) δ = 2.58 (s, 3H, CH_3_), 2.63 (s, 3H, CH_3_), 6.75–7.44 (m, 5H, Ar–H),8.47 (s, 1H, CH), 10.41 (s, 1H, OH), 11.07 (s, 1H, NH); Anal. Calcd for C_18_H_14_N_4_O_3_: C, 64.67; H, 4.22; N, 16.76. Found: C, 64.51; H, 4.35; N, 16.54%.

#### 7-(2,5-Dimethoxyphenyl-2,4-dimethylpyrido[4ʹ,3ʹ:3,4]pyrazolo[1,5-*a*]pyrimidine-8,10(7*H*,9*H*)-dione (7e)

Yellow crystals, yield 90%, m.p. 275 °C, ν_max_/cm^−1^ (KBr) 3195 (NH), 170 (CO); ^1^H NMR (DMSO-*d*_6_) δ = 2.41 (s, 3H, CH_3_), 2.68 (s, 3H, CH_3_), 3.36 (s, 3H, OCH_3_), 3.81 (s, 3H, OCH_3_), 6.71–7.19 (m, 4H, Ar–H), 8.32 (s, 1H, CH), 10.89 (s, 1H, NH); Anal. Calcd for C_20_H_18_N4O_4_: C, 63.49; H, 4.80; N, 14.81. Found: C, 63.31; H, 4.64; N, 14.54%.

#### 2,4-Dimethyl-7-(3,4,5-trimethoxybenzylidene)pyrido[4ʹ,3ʹ:3,4]pyrazolo[1,5-*a*]pyrimidine-8,10(7*H*,9*H*)-dione (7f)

Yellow crystals, yield 82%, m.p.240 °C, ν_max_/cm^−1^ (KBr) 3211 (NH), 1701 (CO); ^13^C NMR (DMSO-*d*_6_) δ = 17.38, 24.77, 56.87, 60.72, 98.47, 111.55, 112.47, 117.18, 129.30, 141.25, 146.45, 150.88, 152.68, 159.58, 163.50, 166.22; Anal. Calcd for C_21_H_20_N_4_O_5_: C, 61.76; H, 4.94; N, 13.72. Found: C, 61.65; H, 4.78; N, 13.51%.

#### 7-(Benzo[*d*][1,3]dioxol-5-ylmethylene)-2,4-dimethylpyrido[4ʹ,3ʹ:3,4]pyrazolo[1,5-*a*]-pyrimidine-8,10(7*H*,9*H*)-dione (7g)

Yellow crystals, yield 65%, m.p. 300 °C, ν_max_/cm^−1^ (KBr) 3169 (NH), 1682(CO);^1^H NMR (DMSO-*d*_6_) δ = 2.57 (s, 3H, CH_3_), 2.67 (s, 3H, CH_3_), 6.14 (s, 2H, CH_2_), 6.98 (d, 1H, *J* = 8.1 Hz, Ar–H), 7.14 (s, 1H, CH), 7.69 (d, 1H, *J* = 7.8 Hz, Ar–H), 8.03 (s, 1H, Ar–H)8.66 (s, 1H, CH), 10.94 (s, 1H, NH); Anal. Calcd for C_19_H_14_N_4_O_4_: C, 62.98; H, 3.89; N, 15.46. Found: C, 62.81; H, 3.70; N, 15.68%.

#### 7-(Furan-2-ylmethylene)-2,4-dimethylpyrido[4ʹ,3ʹ:3,4]pyrazolo[1,5-*a*]pyrimidine-8,10(7*H*,9*H*)-dione (7h)

Brown crystals, yield 61%, m.p 305 °C, ν_max_/cm^−1^ (KBr) 3217 (NH), 1696 (CO);^1^H NMR (DMSO-*d*_6_) δ = 2.60 (s, 3H, CH_3_), 2.81 (s, 3H, CH_3_), 6.88(t, 1H, *J* = 3.6 Hz, Ar–H), 7.22 (s, 1H, CH),7.94 (d, 1H, *J* = 5.4 Hz, Ar–H), 8.14 (s, 1H, CH), 8.70(d, 1H, *J* = 3.64 Hz, Ar–H), 11.04 (s, 1H, NH); Anal. Calcd for C_16_H_12_N_4_O_2_: C, 62.33; H, 3.92; N, 18.17. Found: C, 62.51; H, 3.73; N, 18.40%.

#### 2,4-Dimethyl-7-(thiophen-2-ylmethylene)pyrido[4ʹ,3ʹ:3,4]pyrazolo[1,5-*a*]pyrimidine-8,10(7*H*,9*H*)-dione (7i)

Brown crystals, yield 85%, m.p. 290 °C, ν_max_/cm^−1^ (KBr) 3174 (NH), 1683 (CO);^1^H NMR (DMSO-*d*_6_) δ = 2.62 (s, 3H, CH_3_), 2.91 (s, 3H, CH_3_), 7.24 (s, 1H, CH),7.33 (t, 1H, *J* = 9 Hz, Ar–H), 8.15(d, 1H, *J* = 5.1 Hz, Ar–H), 8.21(d, 1H, *J* = 3.3 Hz, Ar–H), 8.42 (s, 1H, CH), 11.01 (s, 1H, NH); Anal. Calcd for C_16_H_12_N_4_O_2_S: C, 59.25; H, 3.73; N, 17.27; S, 9.88.Found: C, 59.43; H, 3.54; N, 17.49; S, 9.67%.

#### 2,4-Dimethyl-7-(pyridin-4-ylmethylene)pyrido[4ʹ,3ʹ:3,4]pyrazolo[1,5-*a*]pyrimidine-8,10(7*H*,9*H*)-dione (7j)

Brown crystals, yield 81%, m.p. 270 °C, ν_max_/cm^−1^ (KBr) 3435 (NH), 1695(CO);^1^H NMR (DMSO-*d*_6_) δ = 2.49 (s, 3H, CH_3_), 2.60 (s, 3H, CH_3_), 7.24 (s, 1H, CH), 8.03–81.2 (m, 3H, Ar–H), 8.68–8.71 (m, 2H, Ar–H), 11.23 (s, 1H, NH); Anal. Calcd for C_17_H_13_N_5_O_2_: C, 63.94; H, 4.10; N, 21.93. Found: C, 63.81; H, 4.27; N, 21.69%.

#### 7-Benzylidene-2,4-diphenylpyrido[4ʹ,3ʹ:3,4]pyrazolo[1,5-*a*]pyrimidine-8,10(7*H*,9H)-dione (7k)

Brown crystals, yield 67%, m.p. 275 °C, ν_max_/cm^−1^ (KBr) 3231 (NH), 1691 (CO); ^1^H NMR (DMSO-*d*_6_) δ = 7.35 (m, 1H, Ar–H). 7.55–7.91 (m, 4H, Ar–H), 8.03–8.15 (m, 11H, Ar–H), 8.36 (s, 1H, CH), 11.21 (s, 1H, NH); ^13^C NMR (DMSO-*d*_6_) δ = 99.43, 108.54, 118.74, 128.33, 128.66, 128.95, 129.47, 130.52, 130.69, 131.51, 131.79, 131.89, 132.76, 134.05, 136.35, 145.73, 147.61, 147.75, 151.63, 159.30, 159.70, 166.05; Anal. Calcd for C_28_H_18_N_4_O_2_: C, 76.01; H, 4.10; N, 12.66. Found: C, 76.14; H, 4.26; N, 12.43%.

#### 7-(4-Methoxybenzylidene-2,4-diphenylpyrido[4ʹ,3ʹ:3,4]pyrazolo[1,5-*a*]pyrimidine-8,10(7*H*,9*H*)-dione (7l)

Yellow crystals, yield 64%, m.p. 315 °C, ν_max_/cm^−1^ (KBr) 3216 (NH), 1697 (CO); ^1^H NMR (DMSO-*d*_6_) δ = 3.84 (s, 3H, OCH_3_), 7.13–7.21 (m, 6H, Ar–H). 7.52–7.92 (m, 7H, Ar–H), − 8.03–8.21 (m, 3H, Ar–H and CH), 11.09 (s, 1H, NH); Anal. Calcd for C2_9_H_20_N_4_O_3_: C, 73.72; H, 4.27; N, 11.86. Found: C, 73.54; H, 4.40; N, 11.63%.

#### 7-(4-Chlorobenzylidene)-2,4-diphenylpyrido[4ʹ,3ʹ:3,4]pyrazolo[1,5-*a*]pyrimidine-8,10(7*H*,9*H*)-dione (7m)

Brown crystals, yield 63%, m.p. 320 °C, ν_max_/cm^−1^ (KBr) 3209 (NH), 1687 (CO); ^1^H NMR (DMSO-*d*_6_) δ = 7.39 (d, 2H, *J* = 8.7 Hz, CH), 7.60–7.66 (m, 7H, Ar–H and CH), 8.08 (d, 2H, *J* = 8.4 Hz, CH), 8.12 (s, 1H,CH), 8.19–8.21 (m, 2H, Ar–H), 8.42–8.45 (m, 2H, Ar–H), 11.25 (s, 1H, NH);Anal. Calcd for C_28_H_17_ClN_4_O_2_: C, 70.52; H, 3.59; Cl, 7.43; N, 11.75. Found: C, 70.37; H, 3.43; Cl, 7.62; N, 11.53%.

#### 7-(2-Hydroxybenzylidene)-2,4-diphenylpyrido[4ʹ,3ʹ:3,4]pyrazolo[1,5-*a*]pyrimidine-8,10(7*H*,9*H*)-dione (7n)

Brown crystals, yield 75%, m.p. 275 °C, ν_max_/cm^−1^ (KBr) 3426 (OH), 3176 (NH), 1684 (CO); ^1^H NMR (DMSO-*d*_6_) δ = 6.77 (t, 1H, *J* = 7.5 Hz, CH), 6.92 (d, 1H, *J* = 7.8 Hz, CH), 7.32 (t, 1H, *J* = 7.2 Hz, CH), 7.57–7.68 (m, 7H, Ar–H), 8.08 (s, 1H,CH), 8.15 (d, 2H, *J* = 7.5 Hz, CH), 8.40–8.56 (m, 3H, Ar–H), 10.32 (s, 1H, OH), 11.13 (s, 1H, NH); Anal. Calcd for C_28_H_18_N_4_O_3_: C, 73.35; H, 3.96; N, 12.22. Found: C, 73.52; H, 3.77; N, 12.46%.

#### 7-(2,5-Dimethoxybenzylidene)-2,4-diphenylpyrido[4ʹ,3ʹ:3,4]pyrazolo[1,5-*a*]pyrimidine-8,10(7*H*,9*H*)-dione (7o)

Brown crystals, yield 66%, m.p. 275 °C, ν_max_/cm^−1^ (KBr) 3269 (NH), 1700 (CO);1680 (CO); ^1^H NMR (DMSO-*d*_6_) δ = 3.39 (s, 3H, OCH_3_), 3.78 (s, 3H, OCH_3_), 6.99 (d, 1H, *J* = 6.9 Hz, CH), 7.06 (d, 1H, *J* = 6.6 Hz, CH), 7.40 (t, 2H, *J* = 7.5 Hz, CH), 7.56–7.57 (m, 4H, Ar–H), 7.64 (s, 1H, CH), 8.04–8.07 (m, 3H, Ar–H and CH),8.32 (s, 1H, CH), 8.37–8.39 (m, 2H, Ar),11.17 (s, 1H, NH); Anal. Calcd for C_30_H_22_N_4_O_4_: C, 71.70; H, 4.41; N, 11.15. Found: C, 71.55; H, 4.59; N, 11.39%.

#### 2,4-Diphenyl-7-(3,4,5-trimethoxybenzylidene)pyrido[4ʹ,3ʹ:3,4]pyrazolo[1,5-*a*]-pyrimidine-8,10(7*H*,9*H*)-dione (7p)

Brown crystals, yield 65%, m.p. 315 °C, ν_max_/cm^−1^ (KBr) 3186 (NH), 1705 (CO);1677 (CO); ^1^H NMR (DMSO-*d*_6_) δ = 3.40 (s, 6H, 2OCH_3_), 3.76 (s, 3H, OCH_3_), 7.43–7.66 (m, 8H, Ar–H),8.03 (s, 1H, CH), 8.04–8.08 (m, 2H, Ar–H), 8.18 (s, 1H, CH), 8.37–8.39 (m, 2H, Ar–H), 11.16 (s, 1H, NH); Anal. Calcd for C_31_H_24_N_4_O_5_: C, 69.92; H, 4.54; N, 10.52. Found: C, 69.79; H, 4.69; N, 10.76%.

#### 7-(Benzo[*d*][1,3]dioxol-5-ylmethylene)-2,4-diphenylpyrido[4ʹ,3ʹ:3,4]pyrazolo[1,5-a]-pyrimidine-8,10(7*H*,9*H*)-dione (7q)

Brown crystals, yield 89%, m.p. 335 °C, ν_max_/cm^−1^ (KBr) 3227 (NH), 1688 (CO);^13^C NMR (DMSO-*d*_6_) δ = 99.24, 102.36, 108.56, 112.18, 116.01, 128.15, 128.29, 129.15, 129.45, 130.21, 130.69, 130.75, 131.76, 131.86, 136.33, 145.96, 147.68, 150.72, 151.96, 159.22, 159.69, 166.37; Anal. Calcd for C_29_H_18_N_4_O_4_: C, 71.60; H, 3.73; N, 11.52. Found: C, 71.76; H, 3.64; N, 11.73%.

#### 7-(Furan-2-ylmethylene)-2,4-diphenylpyrido[4ʹ,3ʹ:3,4]pyrazolo[1,5-*a*]pyrimidine-8,10(7*H*,9*H*)-dione (7r)

Brown crystals, yield 70%, m.p. 335 °C, ν_max_/cm^−1^ (KBr) 3219 (NH), 1684 (CO); ^1^H NMR (DMSO-*d*_6_) δ = 6.65 (d, 1H, *J* = 5.7 Hz, CH), 7.61–7.63 (m, 3H, Ar–H), 7.72–7.76 (m, 3H, Ar–H), 8.02 (s, 1H,CH), 8.10–8.19 (m, 4H, Ar–H), 8.40–8.46 (m, 3H, Ar–H), 11.19 (s, 1H, NH); Anal. Calcd for C_26_H_16_N_4_O_3_: C, 72.22; H, 3.73; N, 12.96.Found: C, 72.39; H, 3.59; N, 12.73%.

#### 2,4-Diphenyl-7-(thiophen-2-ylmethylene)pyrido[4ʹ,3ʹ:3,4]pyrazolo[1,5-*a*]pyrimidine-8,10(7*H*,9*H*)-dione (7s)

Brown crystals, yield 61%, m.p. 315 °C, ν_max_/cm^−1^ (KBr) 3219 (NH), 1684 (CO); ^1^H NMR (DMSO-*d*_6_) δ = 7.18 (d.d, 1H, *J* = 4.92 Hz, CH), 7.57–7.60 (m, 3H, Ar–H), 7.69–7.76 (m, 3H, Ar–H), 7.96 (d, 1H, *J* = 5 Hz, CH), 8.07 (s, 1H,CH), 8.15 (dd, 2H, *J* = 7.92 Hz, CH),8.35–8.36 (m, 2H, Ar–H), 8.40–8.43 (m, 2H, Ar–H), 11.15 (s, 1H, NH); Anal. Calcd for C_26_H_16_N_4_O_2_S: C, 69.63; H, 3.60; N, 12.49; S, 7.15. Found: C, 69.44; H, 3.76; N, 12.74; S, 7.34%.

#### 2,4-Diphenyl-7-(pyridin-4-ylmethylene)pyrido[4ʹ,3ʹ:3,4]pyrazolo[1,5-a]pyrimidine-8,10(7H,9H)-dione (7t)

Brown crystals, yield 68%, m.p. 340 °C, ν_max_/cm^−1^ (KBr) 3238 (NH), 1696 (CO); ^1^H NMR (DMSO-*d*_6_) δ = 7.57–7.60 (m, 5H, Ar–H), 7.66 (d, 1H, *J* = 7.36 Hz, CH), 7.88 (d, 2H, *J* = 5.56 Hz, CH), 8.01 (d, 2H, *J* = 7.44 Hz, CH), 8.10 (s, 1H, CH), 8.13 (s, 1H, CH), 8.39–8.41 (m, 2H, Ar–H),8.61 (d, 2H, *J* = 5.76 Hz, CH), 11.35 (s, 1H, NH); Anal. Calcd for C_27_H_17_N_5_O_2_: C, 73.13; H, 3.86; N, 15.79. Found: C, 73.32; H, 3.72; N, 15.55%.

### General procedure of synthesis of compounds 9a-d and 11a-d

**Method** **A**: Compound **4a** or **4b** (0.01 mol) and sodium acetate (0.01 mol) were stirred in DMF (5 ml) under cooling in an ice-bath (0–5 °C). To the resulting cold solution is added portionwise a cold solution of the appropriate arenediazonium chlorides **8a**–**d.** The mixture was stirred again under cooling conditions for 3 h., the resulting solid was filtered, washed with water and recrystallized from DMF.

**Method** **B**: A mixture of 3-amino-7-(2-arylhydrazono)-1,7-dihydro-4*H*-pyrazolo[4,3-*c*]pyridine-4,6-diones **10a**–**d** (0.01 mol) and each of acetylacetone **3a** or dibenzoyl methane **3b** was refluxed in DMF (10 ml) in the presence of piperidine for 7 h. The solid that formed was filtered and recrystallized from DMF.

#### 2,4-Dimethyl-7-(2-phenylhydrazono)pyrido[4ʹ,3ʹ:3,4]pyrazolo[1,5-*a*]pyrimidine-8,10(7*H*,9*H*)-dione (9a)

Brown crystals, yield 65% (A), 60% (B), m.p.300 °C, ν_max_/cm^−1^ (KBr) 3140 (NH), 1675 (CO); ^1^H NMR (DMSO-*d*_6_) δ = 2.63 (s, 3H, CH_3_), 2.80 (s, 3H, CH_3_), 7.30 (s, 1H, CH), 7.42–7.54 (m, 5H, Ar–H), 11.01 (s, 1H, NH), 12.43 (s, 1H, NH); Anal. Calcd for C_17_H_14_N_6_O_2_: C, 61.07; H, 4.22; N, 25.14. Found: C, 61.23; H, 4.41; N, 25.36%.

#### 2,4-Dimethyl-7-(2-(*p*-tolyl)hydrazono)pyrido[4ʹ,3ʹ:3,4]pyrazolo[1,5-*a*]pyrimidine-8,10(7*H*,9*H*)-dione (9b)

Yellow crystals, yield 66% (A), 61% (B), m.p. 285 °C, ν_max_/cm^−1^ (KBr) 3187 (NH), 1687 (CO); ^1^H NMR (DMSO-*d*_6_) δ = 2.30 (s, 3H, CH_3_), 2.62 (s, 3H, CH_3_), 2.87 (s, 3H, CH_3_), 7.19–7.42 (m, 5H, Ar–H), 10.94 (s, 1H, NH), 12.40 (s, 1H, NH); m/z 348 = (M^+^, 100%), 319 (5.2%), 304 (8.5%), 257 (3.7%), 229 (12.4%), 199 (99.1%), 174 (35.1%), 158 (23.5%), 105 (28.3%), 91 (67.6%), 77 (42.3%), 65 (38.7%); Anal. Calcd for C_18_H_16_N_6_O_2_: C, 62.06; H, 4.63; N, 24.12. Found: C, 62.25; H, 4.49; N, 24.35%.

#### 7-(2-(4-Methoxyphenyl)hydrazono)-2,4-dimethylpyrido[4ʹ,3ʹ:3,4]pyrazolo[1,5-*a*]pyri-midine-8,10(7*H*,9*H*)-dione (9c)

Brown crystals, yield 72% (A), 68% (B), m.p. 305 °C, ν_max_/cm^−1^ (KBr) 3156 (NH), 1679 (CO); ^1^H NMR (DMSO-*d*_6_) δ = 2.44 (s, 3H, CH_3_), 2.71 (s, 3H, CH_3_), 3.76 (s, 3H, OCH_3_), 7.22–7.63 (m, 5H, Ar–H), 10.82 (s, 1H, NH), 12.11 (s, 1H, NH); m/z 364 = (M^+^, 2.75%), 230 (84.97%), 187 (100%), 158 (16.1%), 132 (5.2%), 108 (9.3%), 78 (6.8%), 65 (5.4%); Anal. Calcd for C_18_H_16_N_6_O_3_: C, 59.34; H, 4.43; N, 23.07. Found: C, 59.47; H, 4.27; N, 23.30%.

#### 7-(2-(4-Chlorophenyl)hydrazono)-2,4-dimethylpyrido[4ʹ,3ʹ:3,4]pyrazolo[1,5-*a*]pyrimidine-8,10(7*H*,9*H*)-dione (9d)

Brown crystals, yield 67% (A), 62% (B), m.p. 300 °C, ν_max_/cm^−1^ (KBr) 3183 (NH), 1676(CO); ^1^H NMR (DMSO-*d*_6_) δ = 2.56 (s, 3H, CH_3_), 2.76 (s, 3H, CH_3_), 7.25 (s, 1H, CH), 7.41–7.51 (m, 4H, Ar–H), 10.96 (s, 1H, NH), 12.26 (s, 1H, NH); Anal. Calcd for C_17_H_13_ClN_6_O_2_: C, 55.37; H, 3.55; Cl, 9.61; N, 22.79. Found: C, 55.51; H, 3.38; Cl, 9.67; N, 22.58%.

#### 2,4-Diphenyl-7-(2-phenylhydrazono)pyrido[4ʹ,3ʹ:3,4]pyrazolo[1,5-*a*]pyrimidine-8,10(7*H*,9*H*)-dione (11a)

Brown crystals, yield 70% (A), 68% (B), m.p. 315 °C, ν_max_/cm^−1^ (KBr) 3369 (NH), 1689(CO); ^1^H NMR (DMSO-*d*_6_) δ = 7.21 (d, 2H, *J* = 7.2 Hz, CH), 7.42–7.46 (m, 3H, Ar–H), 7.60–7.80 (m, 6H, Ar–H),8.20 (s, 1H, CH), 8.29–8.43 (m, 4H, Ar–H),11.09 (s, 1H, NH), 12.36 (s, 1H, NH); Anal. Calcd for C_27_H_18_N_6_O_2_: C, 70.73; H, 3.96; N, 18.33. Found: C, 70.55; H, 3.80; N, 18.55%.

#### 2,4-Diphenyl-7-(2-(*p*-tolyl)hydrazono)pyrido[4ʹ,3ʹ:3,4]pyrazolo[1,5-*a*]pyrimidine-8,10(7*H*,9*H*)-dione (11b)

Yellow crystals, yield 66% (A), 67% (B), m.p. 320 °C, ν_max_/cm^−1^ (KBr) 3189 (NH), 1701 (CO);^1^H NMR (DMSO-*d*_6_) δ = 2.25 (s, 3H, CH_3_), 7.03 (d, 2H, *J* = 8.4 Hz, CH), 7.15 (d, 2H, *J* = 8.1 Hz, CH), 7.56–7.58 (m, 3H, Ar–H), 7.75–7.77 (m, 3H, Ar–H),8.13 (s, 1H, Ar–H),8.25–8.27 (m, 2H, Ar–H),8.38–8.41 (m, 2H, Ar–H),11.01 (s, 1H, NH), 12.27 (s, 1H, NH); Anal. Calcd for C_28_H_20_N_6_O_2_: C, 71.18; H, 4.27; N, 17.79. Found: C, 71.34; H, 4.46; N, 17.54%.

#### 7-(2-(4-Methoxyphenyl)hydrazono)-2,4-diphenylpyrido[4ʹ,3ʹ:3,4]pyrazolo[1,5-*a*]pyri-midine-8,10(7*H*,9*H*)-dione (11c)

Brown crystals, yield 60% (A), 62% (B), m.p. 325 °C, ν_max_/cm^−1^ (KBr) 3265 (NH), 1692 (CO); ^1^H NMR (DMSO-*d*_6_) δ = 3.81 (s, 3H, OCH_3_), 7.14–7.39 (m, 4H, Ar–H), 7.53–7.72 (m, 6H, Ar–H), 8.09 (s, 1H,CH), 8.21–8.39 (m, 4H, Ar–H), 11.07 (s, 1H, NH), 12.35 (s, 1H, NH); Anal. Calcd for C_28_H_20_N_6_O_3_: C, 68.84; H, 4.13; N, 17.20. Found: C, 68.69; H, 4.29; N, 17.43%.

#### 7-(2-(4-Chlorophenyl)hydrazono)-2,4-diphenylpyrido[4ʹ,3ʹ:3,4]pyrazolo[1,5-*a*]pyrimidine-8,10(7*H*,9*H*)-dione (11d)

Brown crystals, yield 72% (A), 68% (B), m.p. 335 °C, ν_max_/cm^−1^ (KBr) 3148 (NH), 1686 (CO); ^1^H NMR (DMSO-*d*_6_) δ = 7.13–7.32 (m, 4H, Ar–H), 7.59–7.85 (m, 6H, Ar–H), 8.13(s, 1H,CH), 8.27–8.45 (m, 4H, Ar–H), 11.13 (s, 1H, NH), 12.42 (s, 1H, NH); m/z 492 = (M^+^, 30.2%), 452 (1.8%), 423 (1.3%), 396 (2.0%), 367 (3.6%), 346 (17.6%), 323 (24.6%), 304 (28.7%), 282 (17.0%), 231 (16.5%), 204 (21.5%), 165 (22.8%), 129 (55.3%), 111 (97.9%), 99 (48.5%), 77 (100%), 43 (85.1%);Anal. Calcd for C_27_H_17_ClN_6_O_2_: C, 65.79; H, 3.48; Cl, 7.19; N, 17.05. Found: C, 65.62; H, 3.32; Cl, 7.41; N, 17.28%.

### General procedure for synthesis of compounds 14a,b

A mixture of compound **1** (0.01 mol) and *β*-ketoesters **12a**,**b** (0.01 mol) was refluxed in glacial acetic acid (20 ml) for 9 h. The resulting solid was collected by filtration and recrystallized from DMF.

#### 4-Hydroxy-2-methylpyrido[4ʹ,3ʹ:3,4]pyrazolo[1,5-*a*]pyrimidine-8,10(7*H*,9*H*)-dione (14a)

Brown crystals, yield 65%, m.p. > 360 °C, ν_max_/cm^−1^ (KBr) 3430 (OH), 3187 (NH), 1689 (CO); ^1^H NMR (DMSO-*d*_6_) δ = 2.37 (s, 3H, CH_3_), 3.95 (s, 2H, CH_2_), 5.86 (s, 1H, CH), 10.96 (s, 1H, NH), 12.80 (s, 1H, OH); m/z 232 = (M^+^, 100%), 214 (29.3%), 204 (5.7%), 189 (5.1%), 175 (2.1%), 159 (9.0%), 148 (5.2%), 133 (40.2%), 120 (4.1%), 105 (11.2%), 92 (7.4%), 78 (9.2%), 65 (29.2%), 52 (7.9%);Anal. Calcd for C_10_H_8_N_4_O_3_: C, 51.73; H, 3.47; N, 24.13.Found: C, 51.57; H, 3.28; N, 24.36%.

#### 4-Hydroxy-2-phenylpyrido[4ʹ,3ʹ:3,4]pyrazolo[1,5-*a*]pyrimidine-8,10(7*H*,9*H*)-dione (14b)

Brown crystals, yield 65%, m.p. 310 °C, ν_max_/cm^−1^ (KBr) 3404 (OH),3189 (NH), 1688 (CO); ^1^H NMR (DMSO-*d*_6_) δ = 3.83 (s, 1H, OH), 4.00 (s, 2H, CH_2_), 6.22 (s, 1H, CH), 7.51–7.59 (m, 3H, Ar–H), 7.74–7.77 (m, 2H, Ar–H), 10.97 (s, 1H, NH); m/z 294 = (M^+^, 100%), 276 (22.2%), 265 (1.8%), 251 (4.6%), 232 (2.9), 220 (3.9%), 195 (21.1%), 166 (29.4%), 140 (6.4%), 129 (20.2%), 123 (26.0%), 102 (66.3%), 92 (4.7%), 76 (17.8%), 66 (28.8%), 51 (13.0%);Anal. Calcd for C_15_H_10_N_4_O_3_: C, 61.22; H, 3.43; N, 19.04. Found: C, 61.37; H, 3.26; N, 19.26%.

### *Generalprocedureforsynthesisofcompounds15a*–*f*

Refluxing of a mixture of compounds **14a**,**b** (0.01 mol) and aldehydes **6a**–**c** (0.01 mol) in DMF (20 ml) in a few drops of piperidine for 10 h. The solid that formed was filtered and crystallized from DMF.

#### 7-Benzylidene-4-hydroxy-2-methylpyrido[4ʹ,3ʹ:3,4]pyrazolo[1,5-*a*]pyrimidine-8,10(7*H*,9*H*)-dione (15a)

Brown crystals, yield 79%, m.p. 305 °C, ν_max_/cm^−1^ (KBr) 3419 (OH), 3203 (NH), 1704 (CO); ^13^C NMR (DMSO-*d*_6_) δ = 19.38, 95.29, 100.20, 118.19, 128.71, 131.98, 133.48, 133.81, 141.91, 145.54, 148.10, 152.58, 155.27, 160.24, 166.42; Anal. Calcd for C_17_H_12_N_4_O_3_: C, 63.75; H, 3.78; N, 17.49.Found: C, 63.66; H, 3.65; N, 17.71%.

#### 4-Hydroxy-7-(4-methoxybenzylidene)-2-methylpyrido[4ʹ,3ʹ:3,4]pyrazolo[1,5-*a*]pyrimidine-8,10(7*H*,9*H*)-dione (15b)

Brown crystals, yield 84%, m.p. 345 °C, ν_max_/cm^−1^ (KBr) 3423 (OH), 3195 (NH), 1708 (CO); ^1^H NMR (DMSO-*d*_6_) δ = 2.41 (s, 3H, CH_3_), 3.89 (s, 3H, OCH_3_), 5.95 (s, 1H, CH), 7.07 (d, 2H, *J* = 8.8 Hz, Ar–H), 8.19 (s, 1H, CH), 8.75 (d, 2H, *J* = 8.8 Hz, Ar–H), 11.21 (s, 1H, NH), 12.75 (s, 1H, OH); Anal. Calcd for C_18_H_14_N_4_O_4_: C, 61.71; H, 4.03; N, 15.99.Found: C, 61.71; H, 4.03; N, 15.99%.

#### 7-(4-Chlorobenzylidene)-4-hydroxy-2-methylpyrido[4ʹ,3ʹ:3,4]pyrazolo[1,5-*a*]pyrimidine-8,10(7*H*,9*H*)-dione (15c)

Brown crystals, yield 65%, m.p. 330 °C, ν_max_/cm^−1^ (KBr) 3434 (OH), 3188 (NH), 1713 (CO);^1^H NMR (DMSO) δ = 2.39 (s, 3H, CH_3_), 5.93 (s, 1H, Ar–H), 7.53 (dd, 2H, *J* = 8.7 Hz, Ar–H), 8.16 (s, 1H, CH), 8.52 (d, 2H, *J* = 7.8 Hz, Ar–H), 11.29 (s, 1H, NH), 12.76 (s, 1H, OH); Anal. Calcd for C_17_H_11_ClN_4_O_3_: C, 57.56; H, 3.13; Cl, 9.99; N, 15.79.Found: C, 57.40; H, 3.32; Cl, 9.82; N, 15.57%.

#### 7-Benzylidene-4-hydroxy-2-phenylpyrido[4ʹ,3ʹ:3,4]pyrazolo[1,5-*a*]pyrimidine-8,10(7*H*,9*H*)-dione (15d)

Brown crystals, yield 69%, m.p. 300 °C, ν_max_/cm^−1^ (KBr) 3387 (OH), 3179 (NH), 1689 (CO); ^1^H NMR (DMSO-*d*_6_) δ = 3.37 (s, 1H, OH), 6.24 (s, 1H, CH), 7.23–7.56 (m, 6H, Ar), 7.61–7.67 (m, 4H, Ar), 8.11 (s, 1H, CH), 11.33 (s, 1H, NH); Anal. Calcd for C_22_H_14_N_4_O_3_: C, 69.10; H, 3.69; N, 14.65.Found: C, 69.26; H, 3.56; N, 14.44%.

#### 4-Hydroxy-7-(4-methoxybenzylidene)-2-phenylpyrido[4ʹ,3ʹ:3,4]pyrazolo[1,5-*a*]pyrimidine-8,10(7*H*,9*H*)-dione (15e)

Brown crystals, yield 63%, m.p. 300 °C, ν_max_/cm^−1^ (KBr) 3401 (OH), 3197 (NH), 1705 (CO); ^1^H NMR (DMSO-*d*_6_) δ = 3.39 (s, 1H, OH), 3.76 (s, 3H, OCH_3_), 6.23 (s, 1H, CH), 7.11–7.42 (m, 5H, Ar–H), 7.51–7.59 (m, 4H, Ar–H), 8.06 (s, 1H, CH), 11.41 (s, 1H, NH); ^13^ C NMR (DMSO-*d*_6_) δ = 56.06, 79.24, 79.50, 79.79, 99.59, 114.25, 114.42, 116.33, 127.0, 128.72, 129.05, 135.72, 136.75, 139.50, 142.46, 145.64, 145.83, 160.0, 161.24, 162.09, 166.81, 167.19, 170.02; Anal. Calcd for C_23_H_16_N_4_O_4_: C, 66.99; H, 3.91; N, 13.59.Found: C, 66.82; H, 3.79; N, 13.83%.

#### 7-(4-Chlorobenzylidene)-4-hydroxy-2-phenylpyrido[4ʹ,3ʹ:3,4]pyrazolo[1,5-*a*]pyrimidine-8,10(7*H*,9*H*)-dione (15f)

Brown crystals, yield 72%, m.p. 330 °C, ν_max_/cm^−1^ (KBr) 3412 (OH), 3197 (NH), 1704 (CO); ^1^H NMR (DMSO-*d*_6_) δ = 3.34 (s, 1H, OH), 6.28 (s, 1H, CH), 7.54–7.60 (m, 5H, Ar–H), 7.76–7.79 (m, 2H, Ar–H), 8.17 (s, 1H, CH), 8.52 (d, 2H, *J* = 8.4 Hz, Ar–H), 11.30 (s, 1H, NH); Anal. Calcd for C_22_H_13_ClN_4_O_3_: C, 63.39; H, 3.14; Cl, 8.50; N, 13.44. Found: C, 63.53; H, 3.31; Cl, 8.33; N, 13.67%.

### General procedure of synthesis of compounds 16a–h

A cold solution of arenediazonium chlorides **8a**–**d** was added drop wise in an ice-bath (0–5 °C) to a mixture of compound **4a** (0.01 mol) and sodium acetate (0.01 mol) in DMF (5 ml), after stirring for 3 h. After forming, the resultant solid was filtrated, washed with water and recrystallized from DMF.

#### 4-Hydroxy-2-methyl-7-(2-phenylhydrazono)pyrido[4ʹ,3ʹ:3,4]pyrazolo[1,5-*a*]pyrimid-ine-8,10(7*H*,9*H*)-dione (16a)

Brown crystals, yield 69%, m.p. 336 °C, ν_max_/cm^−1^ (KBr) 3411 (OH), 3201 (NH), 1702 (CO); ^1^H NMR (DMSO-*d*_6_) δ = 2.33 (s, 3H, CH_3_), 5.93 (s, 1H, CH), 7.11–7.56 (m, 5H, Ar–H), 11.08 (s, 1H, NH), 12.41 (s, 1H, NH), 12.94 (s, 1H, OH); m/z 336 = (M^+^, 46.3%), 307 (3.1%), 298 (15.8%), 270 (15.8%), 259 (7.2%), 232 (9.1%), 203 (14.0%), 176 (62.4%), 133 (14.3%), 121 (15.9%), 105 (20.3%), 91 (43.2%), 77 (100%), 44 (50.2%); Anal. Calcd for C_16_H_12_N_6_O_3_: C, 57.14; H, 3.60; N, 24.99. Found: C, 57.33; H, 3.47; N, 24.73%.

#### 4-Hydroxy-2-methyl-7-(2-(*p*-tolyl)hydrazono)pyrido[4ʹ,3ʹ:3,4]pyrazolo[1,5-*a*]pyrimidine-8,10(7*H*,9*H*)-dione (16b)

Brown crystals, yield 71%, m.p. 330 °C, ν_max_/cm^−1^ (KBr) 3441 (OH), 3183 (NH), 1694 (CO); ^1^H NMR (DMSO-*d*_6_) δ = 2.34 (s, 3H, CH_3_), 2.43 (s, 3H, CH_3_), 5.79–5.90 (m, 2H, Ar–H), 7.19–7.35 (m, 3H, Ar–H), 11.05 (s, 1H, NH), 12.33 (s, 1H, NH), 12.78 (s, 1H, OH); Anal. Calcd for C_17_H_14_N_6_O_3_: C, 58.28; H, 4.03; N, 23.99. Found: C, 58.44; H, 4.21; N, 23.74%.

#### 4-Hydroxy-7-(2-(4-methoxyphenyl)hydrazono)-2-methylpyrido[4ʹ,3ʹ:3,4]pyrazolo[1,5-*a*]pyrimidine-8,10(7*H*,9*H*)-dione (16c)

Brown crystals, yield 67%, m.p. 321 °C, ν_max_/cm^−1^ (KBr) 3426 (OH), 3195 (NH), 1702 (CO); ^1^H NMR (DMSO-*d*_6_) δ = 2.41 (s, 3H, CH_3_), 3.76 (s, 3H, OCH_3_), 5.91 (s, 1H, CH), 7.43–7.66 (m, 4H, Ar–H), 11.19 (s, 1H, NH), 12.37 (s, 1H, NH), 12.87 (s, 1H, OH); m/z 366 (M^+^, 49.7%), 338 (4.6%), 300 (6.2%), 298 (97.6%), 270 (100%), 242 (35.7%), 232 (17.5%), 201 (16.9%), 176 (22.3%), 159 (15.3%), 133 (18.1%), 121 (79.4%), 102 (42.8%), 77 (72.8%), 67 (95.2%), 51 (28.5%); Anal. Calcd for C_17_H_14_N_6_O_4_: C, 55.74; H, 3.85; N, 22.94. Found: C, 55.87; H, 3.69; N, 22.73%.

#### 7-(2-(4-Chlorophenyl)hydrazono)-4-hydroxy-2-methylpyrido[4ʹ,3ʹ:3,4]pyrazolo[1,5-*a*]pyrimidine-8,10(7*H*,9*H*)-dione (16d)

Brown crystals, yield 65%, m.p. 310 °C, ν_max_/cm^−1^ (KBr) 3436 (OH), 3181 (NH), 1689 (CO); ^1^H NMR (DMSO-*d*_6_) δ = 2.39 (s, 3H, CH_3_), 5.98 (s, 1H, CH), 7.36 (d, 2H, *J* = 8.7 Hz, Ar–H), 7.46 (d, 2H, *J* = 8.7 Hz, Ar–H), 11.15 (s, 1H, NH), 12.38 (s, 1H, NH), 12.95 (s, 1H, OH); Anal. Calcd for C_16_H_11_ClN_6_O_3_: C, 51.83; H, 2.99; Cl, 9.56; N, 22.67. Found: C, 51.69; H, 2.82; Cl, 9.71; N, 22.43%.

#### 4-Hydroxy-2-phenyl-7-(2-phenylhydrazono)pyrido[4ʹ,3ʹ:3,4]pyrazolo[1,5-*a*]pyrimidine-8,10(7*H*,9*H*)-dione (16e)

Brown crystals, yield 61%, m.p. 300 °C, ν_max_/cm^−1^ (KBr) 3411 (OH), 3173 (NH), 1706 (CO); ^1^H NMR (DMSO-*d*_6_) δ = 3.51 (s, 1H, OH), 6.35 (s, 1H, Ar–H), 7.21–7.33 (m, 5H, Ar–H), 7.44–7.60 (m, 3H, Ar–H), 7.75–7.80 (m, 2H, Ar–H), 11.09 (s, 1H, NH), 12.54 (s, 1H, NH); Anal. Calcd for C_21_H_14_N_6_O_3_: C, 63.31; H, 3.54; N, 21.10. Found: C, 63.44; H, 3.38; N, 21.36%.

#### 4-Hydroxy-2-phenyl-7-(2-(*p*-tolyl)hydrazono)pyrido[4ʹ,3ʹ:3,4]pyrazolo[1,5-*a*]pyrimidine-8,10(7*H*,9H)-dione (16f)

Brown crystals, yield 63%, m.p. 320 °C, ν_max_/cm^−1^ (KBr) 3437 (OH), 3186 (NH), 1685 (CO); ^1^H NMR (DMSO) δ = 2.31 (s, 3H, CH_3_), 3.39 (s, 1H, OH), 6.35 (s, 1H, Ar), 7.21–7.33 (m, 4H, Ar), 7.44–7.60 (m, 3H, Ar), 7.75–7.80 (m, 2H, Ar), 11.09 (s, 1H, NH), 12.54 (s, 1H, NH); Anal. Calcd for C_22_H_16_N_6_O_3_: C, 64.07; H, 3.91; N, 20.38. Found: C, 64.25; H, 3.77; N, 20.62%.

#### 4-Hydroxy-7-(2-(4-methoxyphenyl)hydrazono)-2-phenylpyrido[4ʹ,3ʹ:3,4]pyrazolo[1,5-*a*]pyrimidine-8,10(7*H*,9*H*)-dione (16g)

Brown crystals, yield 64%, m.p. 323 °C, ν_max_/cm^−1^ (KBr) 3418 (OH), 3205 (NH), 1697 (CO); ^1^H NMR (DMSO-*d*_6_) δ = 3.48 (s, 1H, OH), 3.81 (s, 3H, OCH_3_), 6.35 (s, 1H, Ar–H), 7.21–7.33 (m, 4H, Ar–H), 7.44–7.60 (m, 3H, Ar–H), 7.75–7.80 (m, 2H, Ar–H), 11.09 (s, 1H, NH), 12.54 (s, 1H, NH); ^13^C NMR (DMSO-*d*_6_) δ = 55.73, 79.22, 79.75, 99.42, 100.0, 115.53, 117.03, 117.64, 128.72, 129.0, 129.33, 131.40, 136.17, 147.60, 155.80, 157.0, 159.40, 161.89, 162.20, 169.83, 170.91, 171.27; Anal. Calcd for C_22_H_16_N_6_O_4_: C, 61.68; H, 3.76; N, 19.62. Found: C, 61.47; H, 3.62; N, 19.89%.

#### 7-(2-(4-Chlorophenyl)hydrazono)-4-hydroxy-2-phenylpyrido[4ʹ,3ʹ:3,4]pyrazolo[1,5-*a*]pyrimidine-8,10(7*H*,9*H*)-dione (16h)

Brown crystals, yield 76%, m.p. 330 °C, ν_max_/cm^−1^ (KBr) 3429 (OH), 3199 (NH), 1709 (CO); ^1^H NMR (DMSO-*d*_6_) δ = 3.52 (s, 1H, OH), 6.35 (s, 1H, Ar–H), 7.21–7.33 (m, 4H, Ar–H), 7.44–7.60 (m, 3H, Ar–H), 7.75–7.80 (m, 2H, Ar), 11.09 (s, 1H, NH), 12.54 (s, 1H, NH); Anal. Calcd for C_21_H_13_ClN_6_O_3_: C, 58.28; H, 3.03; Cl, 8.19; N, 19.42. Found: C, 58.15; H, 3.22; Cl, 8.41; N, 19.19%

## Biological investigation

### Materials and methods

#### Cell line

The three cell lines MCF7, HePG2 and HTC 116 were obtained from ATCC via Holding company for biological products and vaccines (VACSERA), Cairo, Egypt. Doxorubicin was used as a standard anticancer drug for comparison.

#### Chemical reagents

The reagents are RPMI-1640 medium, MTT and DMSO (sigma co., St. Louis, USA), Fetal Bovine serum (GIBCO, UK).

#### MTT assay

Determination of the inhibitory effects of compounds on cell growth was performed through the MTT assay [35, 36]. This colorimetric assay is based on the conversion of the yellow tetrazolium bromide (MTT) to a purple formazan derivative by mitochondrial succinate dehydrogenase in viable cells. The protocol was discussed in details in Additional file 1.

#### Tropomyosin receptor kinase A (TrKA) inhibitory assay

The TrkA assay Kit is designed to measure TrkA activity for screening and profiling applications using Kinase-Glo® MAX as a detection reagent. The TrkA Assay Kit comes in a convenient 96-well format, with enough purified recombinant TrkA enzyme, TrkA substrate, ATP and kinase assay buffer for 100 enzyme reactions. The method was discussed in details in the ESI.

#### In-vitro cell cycle analysis

HepG-2 cells are pre-cultured in 25 cm^2^ cell culture flask. RPMI-1640 medium was used. Tested compounds **7b**, **9c**, **15b**, **16a** and **16c** were used in the cell treatment at their IC_50_ by dissolving them in the required medium separately. The procedure was discussed in details in the ESI.

#### Annexin V-FITC apoptosis assay

HepG-2 cells were harvested and incubated with compounds **7b**, **15b**, **16a** and **16c** separately for 48 h. Then, the cells were collected and washed with PBS two successive times followed by centrifugation. After that, the cells were treated with Annexin V-FITC and propidium iodide (PI) using the apoptosis detection kit (BD Biosciences and Annexin V-FITC and PI binding were analyzed by a flow cytometer.

#### Molecular docking study

Molecular docking study was performed using program “Molecular Operating Environment (MOE) 2009. The protein structure was downloaded from the PDB data bank (http://www.rcsb.org/PDB codes: 5H3Q). The steps were discussed in details in the ESI.

#### In silico ADME studies

Physicochemical characteristics of **4a**, **7a**–**c**, **9c**, **15b**, **16a**, and **16c** were detected through Swiss Target Predication methodology [37, 38].

### Results and discussion

#### Chemistry

**Scheme 1:**
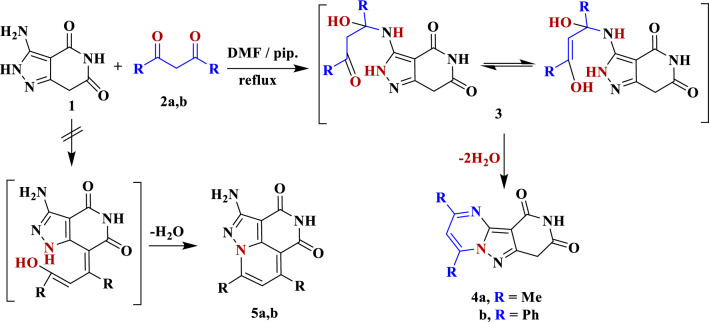
Synthesis of pyrido[4ʹ,3ʹ:3,4]pyrazolo[1,5-*a*]pyrimidines **4a**,**b**

**Scheme 2:**
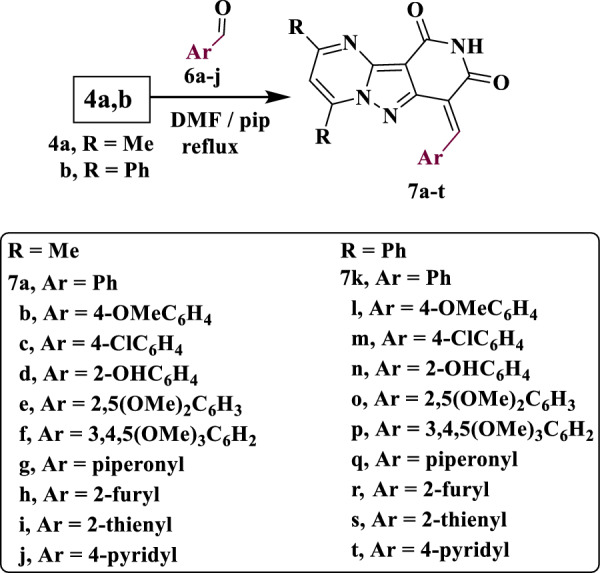
Synthesis of compounds **7a**–**t**

Reactivity of 3-amino-1,7-dihydro-4*H*-pyrazolo[4,3-*c*]pyridine-4,6(5*H*)-dione **1** as a precursor of some heterocycles of interesting biological activity [24, 39] encouraging us to continue our research on the synthesis of new compounds as a potential anticancer agents. Thus, condensation of compound **1** with each of acetylacetone **2a** and dibenzoylmethane **2b**, respectively, in *N,N*-dimethylformamide with a few drops of piperidine afforded products **4a**, **b**. The structures of **4a**, **b** were proven by spectroscopic techniques (Scheme 1). The IR spectrum of compound **4a** shows absorption bands at 3187 and 1702 cm^−1^ assigned to the NH and CO groups, respectively. Its ^1^H NMR spectrum revealed three singlet signals assigned to the two methyl and methylene protons at δ = 2.58, 2.70 and 4.05 ppm, respectively, in addition to a singlet signal at δ = 7.16 ppm for pyrimidine proton. In addition, the *D*_*2*_*O* exchangeable signal appeared at δ = 10.82 ppm corresponding to the NH proton. Furthermore, the mass spectrum of **4a** displayed a molecular ion peak at m/z = 230 (M^+^, 67.6%), consistent with the molecular formula C_11_H_10_N_4_O_2_ (Scheme 2).

The mechanism of the formation of compounds **4a**, **b** was suggested to proceed through nucleophilic attack of the exocyclic amino group in compound **1** on the ketonic function of acetylacetone **2a**, followed by intramolecular cyclization with elimination of water from the intermediate 3 to produce. The other pathway that leads to formation of compounds **5a**, **b** was excluded as shown in Scheme 1.

Each of compounds **4a** (and **4b**) was condensed with the appropriate aromatic aldehyde **6a**–**j** in refluxing DMF in presence of traces of piperidine to yield the respective arylmethylene derivatives **7a**–**t**. The structures of **7a**–**t** were supported by spectroscopic techniques. Compound **7a** exhibits absorption bands in its IR chart at ν 3181 and 1698 cm^−1^ assigned to the NH and CO groups, respectively. Its ^13^C NMR exhibited characteristic signals at 17.02 (CH_3_), 128.34 (CH=C), 163.62 (CO) and 166.00 (CO), in addition to the expected signals (Scheme 2).

**Scheme 3:**
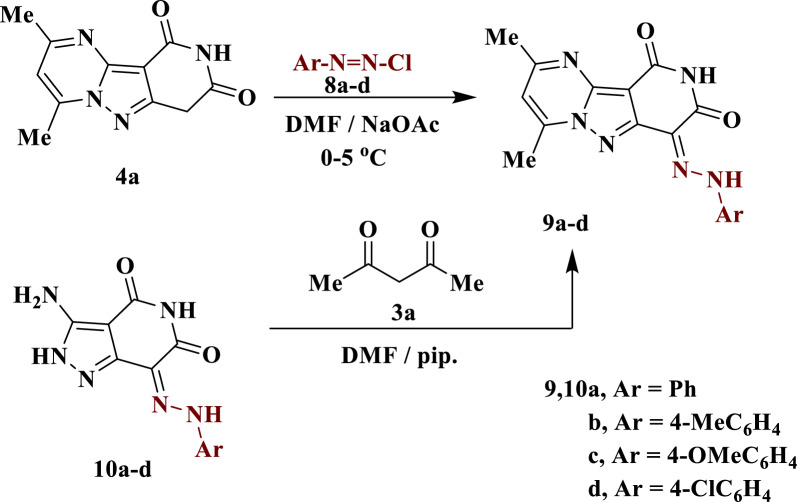
Synthetic route of arylhydrazonopyrido[4ʹ,3ʹ:3,4]pyrazolo[1,5-*a*]pyrimidine diones

Further coupling of compound **4a** with arenadiazonium salts **8a**–**d** in DMF containing sodium acetate at 0–5 °C afforded the corresponding arylhydrazono derivatives **9a**–**d** (Scheme 3). The resulting structures were established by elemental analysis and spectroscopic data. For example, the IR spectrum of **9b** is characterized by the presence of absorption bands at 3187 and 1687 cm^−1^ due to the NH and CO groups, respectively. Also, in ^1^H NMR spectrum appeared three singlet signals at δ = 2.30, 2.62 and 2.87 ppm due to three methyl groups, as well as two other singlet signals that can be exchanged with *D*_*2*_*O* at δ = 10.94 and 12.40 ppm due to two NH protons. The mass spectrum showed a molecular ion peak at m/z = 348 (M^+^, 100%), corresponding to the molecular formula C_18_H_16_N_6_O_2.._ Compounds **9a**–**d** were also obtained by an alternative chemical route by condensing aryl hydrazo derivatives **10a**–**d** [24] with acetylacetone **3a** under reflux conditions in DMF using piperidine as basic medium (Scheme 3).

**Scheme 4:**
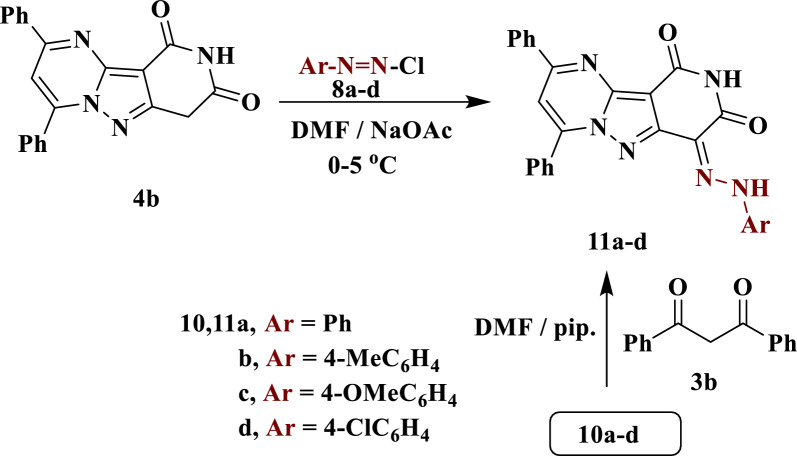
Synthesis of arylhydrazono derivatives **11a**–**d**

Similarly, **4b** coupled with arenadiazonium salts **8a**–**d** under the same reaction conditions to produce arylhydrazono derivatives **11a**–**d** (Scheme 4). The structures generated are supported by spectroscopic data (see exp.). Compounds **11a**–**d** were also obtained by condensation of each of compounds **10a**–**d** with dibenzoylmethane **3b**, as shown in Scheme 4.

**Scheme 5:**
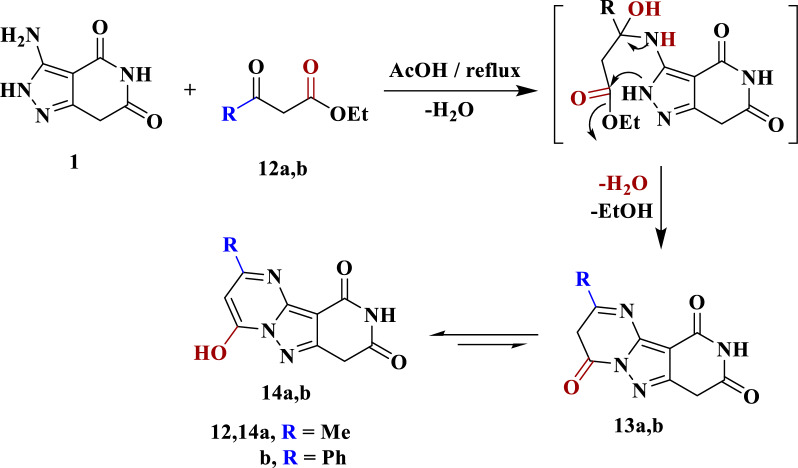
Synthetic route for 4-hydroxy-2-substituted pyrido[4ʹ,3ʹ:3,4]pyrazolo[1,5-*a*]-pyrimidine-8,10-diones **14a**,**b**

On the other hand, cyclocondensation of compound **1** with *β*-Ketoesters **12a**,**b** in glacial acetic acid upon reflux led to the formation of products **14a**,**b** (Scheme 5), which were confirmed by spectroscopic tools. The IR spectrum of compound **14a** was characterized by the presence of absorption bands at 3430, 3187 and 1689 cm^−1^ assigned to the OH, NH and CO groups, respectively. The ^1^H NMR spectrum also revealed a singlet signals assigned to methyl, methylene, pyrimidine-H, NH and OH protons at δ = 2.37, 3.95, 5.86, 10.96, and 12.80 ppm, respectively. The mass spectrum also exhibited a molecular ion peak at m/z = 232 = (M^+^, 100%), confirming that the molecular formula C_10_H_8_N_4_O_3._ The mechanism of formation of **14** is thought to occur initially by nucleophilic attack of the exocyclic amino group on **1** into the ketonic function of *β*-ketoesters **12a**,**b** leading to the elimination of water molecule, followed by the intramolecular cyclization, followed by elimination of the ethanol molecule to obtain the enol structure **14** instead of the keto form **13** as shown in Scheme 5.

**Scheme 6:**
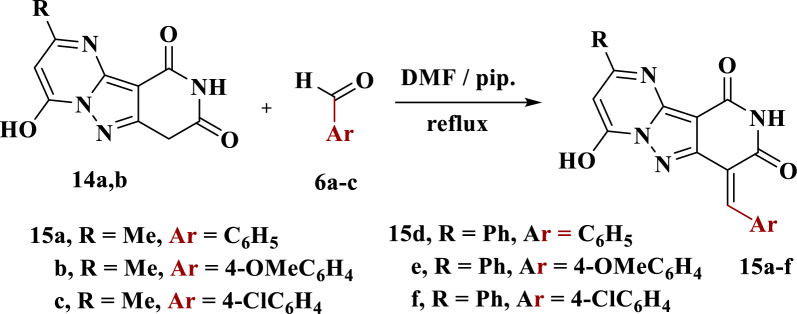
Synthesis of arylmethylene derivatives **15a**–**f**

Condensation of each of **14a**,**b** with the suitable aromatic aldehyde **6a**–**c** in DMF under reflux conditions using a few drops of piperidine yielded the respective arylmethylene derivatives **15a**–**f** (Scheme 6). The IR spectrum of **15a** presented absorption bands at 3419, 3203 and 1704 cm^−1^ assigned to OH, NH and CO groups, respectively. Its ^13^C NMR chart revealed the characteristic signals at 19.38 (CH_3_), 152.58 (CO), 155.27 (CO), 160.24 (C=N) and 166.42 (=C–OH) in addition to other signals assigned for aromatic carbons (see exp.).

**Scheme 7:**
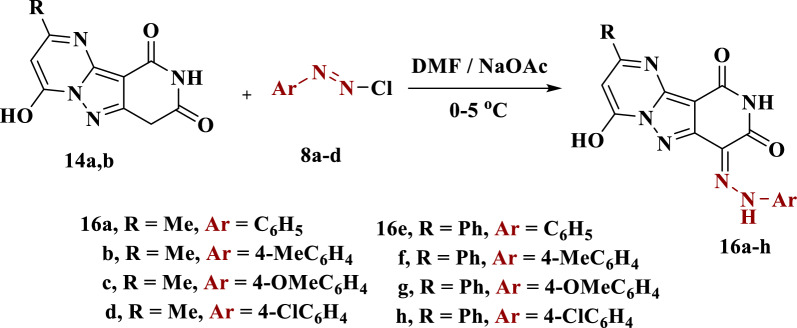
Synthesis of arylhydrazono derivatives **16a**–**h**

The coupling reaction of compounds **14a**,**b** with arenediazonium chlorides **8a**–**d** in DMF containing sodium acetate at 0–5 °C yielded the corresponding arylhydrazono derivatives **16a**–**h**. The structure of **16a**–**h** was determined by elemental analysis and spectral data. The IR spectrum of compound **16c** was characterized by the presence of absorption bands at 3426, 3195 and 1702 cm^−1^ owing to the OH, NH and CO groups, respectively. Moreover, ^1^H NMR chart of compound **16c** appeared two singlets at δ = 2.41 and 3.76 ppm due to methyl and methoxy protons along with three other singlet signals exchangeable with *D*_*2*_*O* in the region 11.19, 12.37 and 12.87 ppm due to three protons of 2NH and OH. The mass spectrum also showed a molecular ion peak at m/z = 366 (M^+^, 49.7%), which confirmed its molecular formula C_17_H_14_N_6_O_4_ (Scheme 7).

## Biological activity

### Anticancer activity

**Fig. 5:**
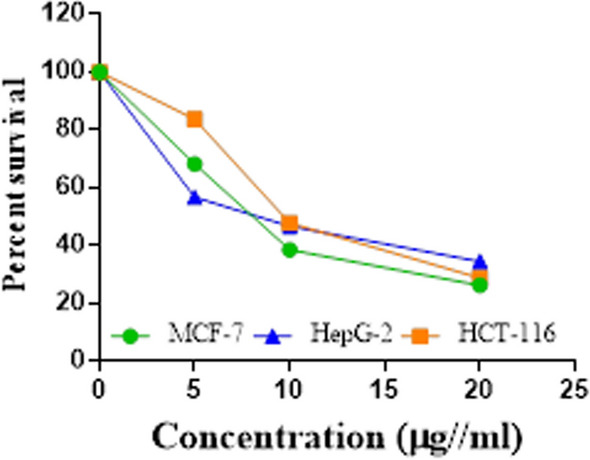
IC_50_ values of **1**

**Fig. 6:**
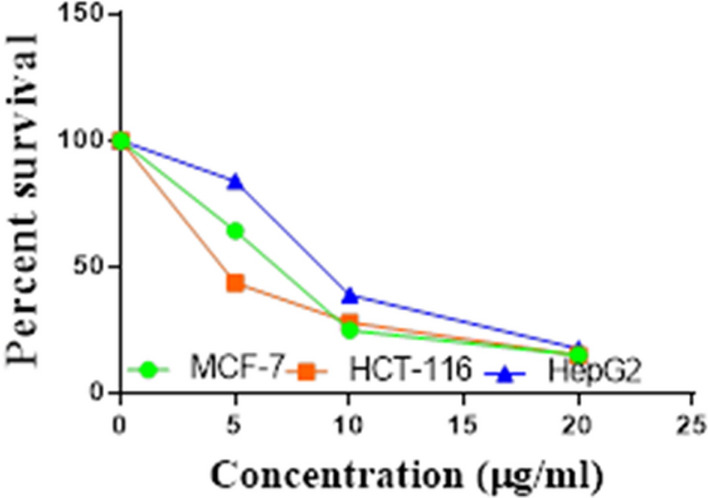
IC_50_ values of **4a**

**Fig. 7:**
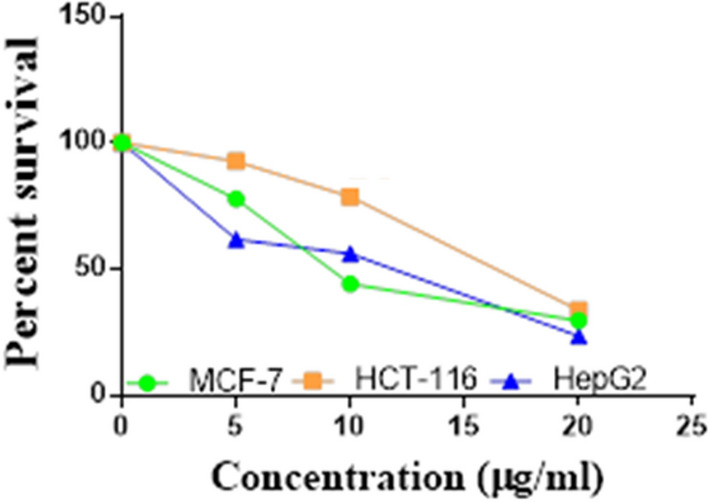
IC_50_ values of **4b**

**Fig. 8:**
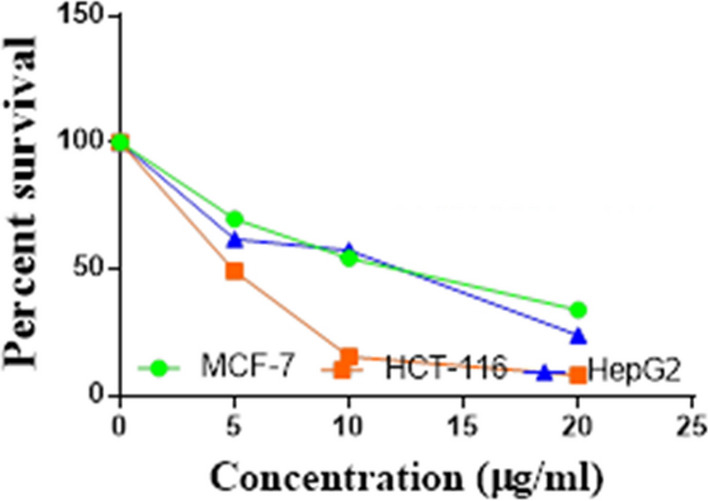
IC_50_ values of **7a**

**Fig. 9:**
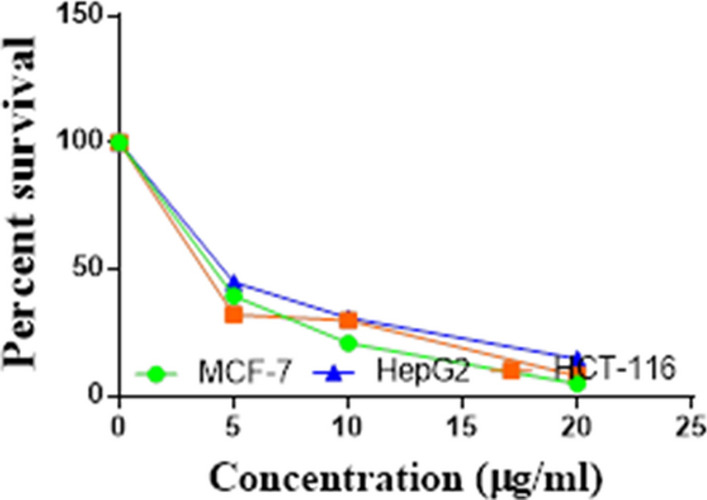
IC_50_ values of **7b**

**Fig. 10:**
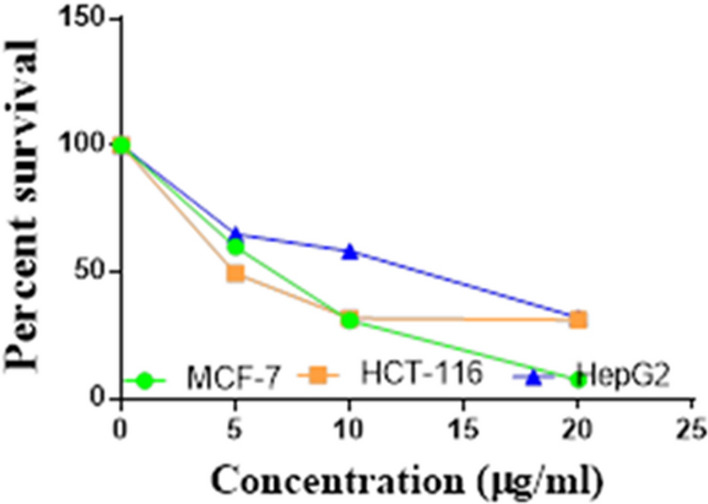
IC_50_ values of **7c**

**Fig. 11:**
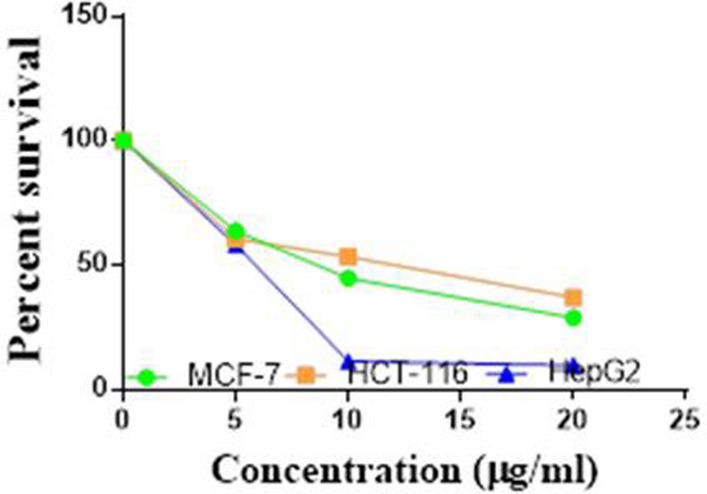
IC_50_ values of **7k**

**Fig. 12:**
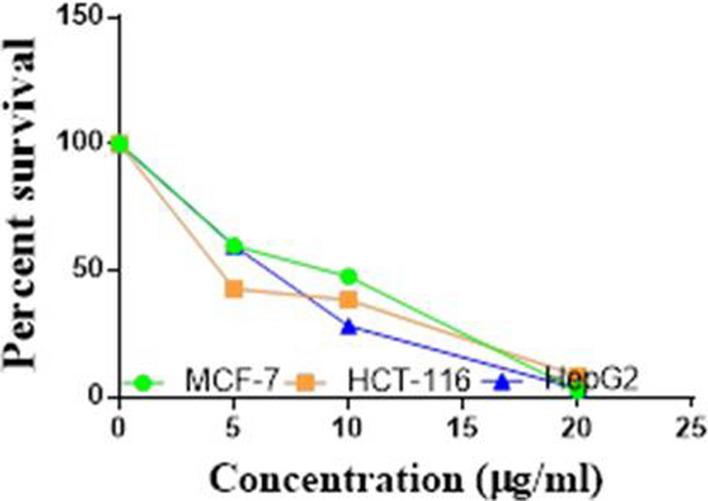
IC_50_ values of **7l**

**Fig. 13:**
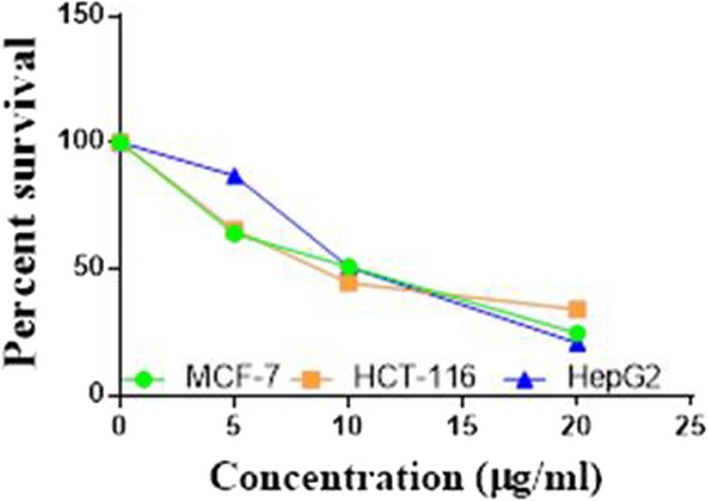
IC_50_ values of **9a**

**Fig. 14:**
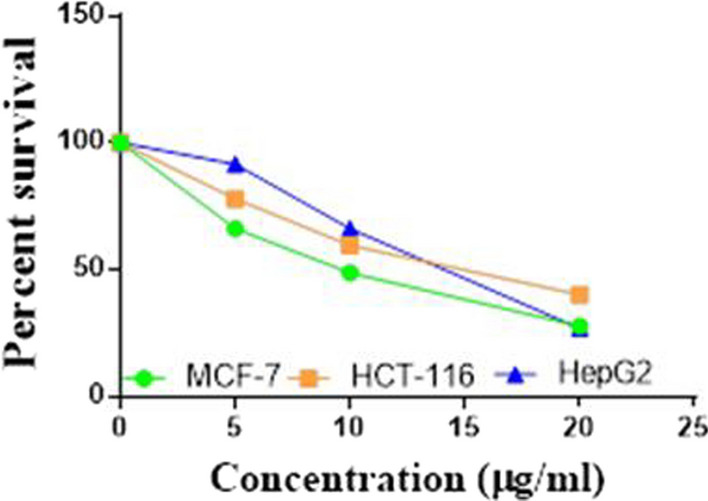
IC_50_ values of **9b**

**Fig. 15:**
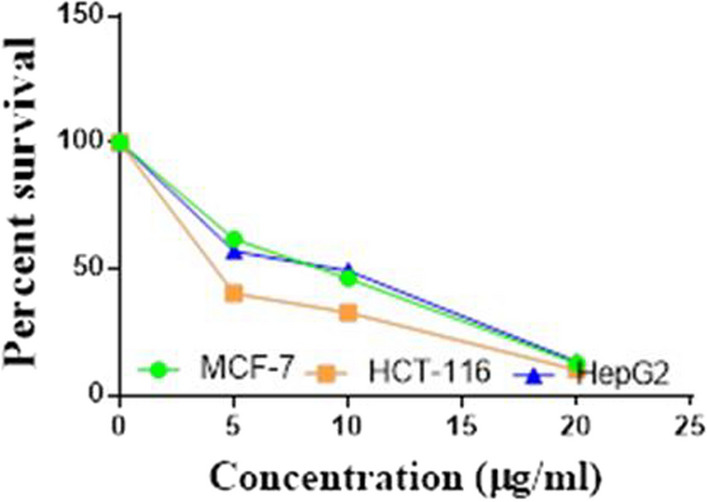
IC_50_ values of **9c**

**Fig. 16:**
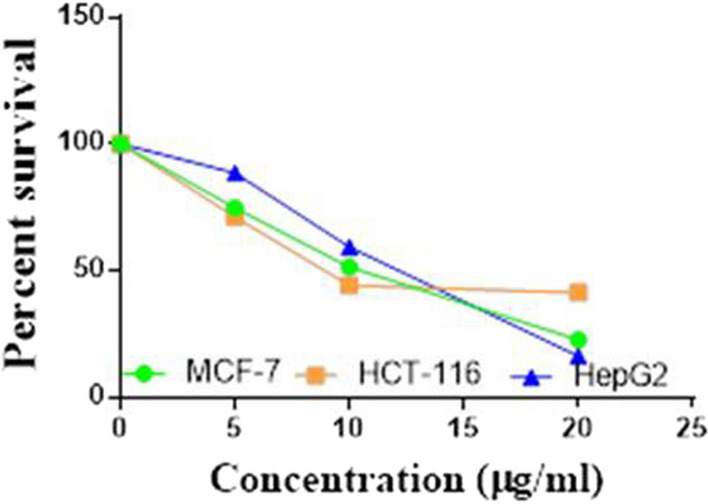
IC_50_ values of **14a**

**Fig. 17:**
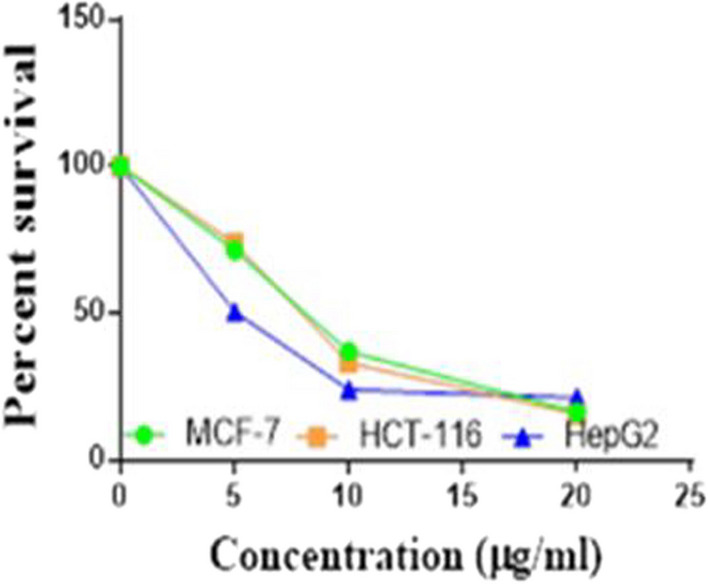
IC_50_ values of **15b**

**Fig. 18:**
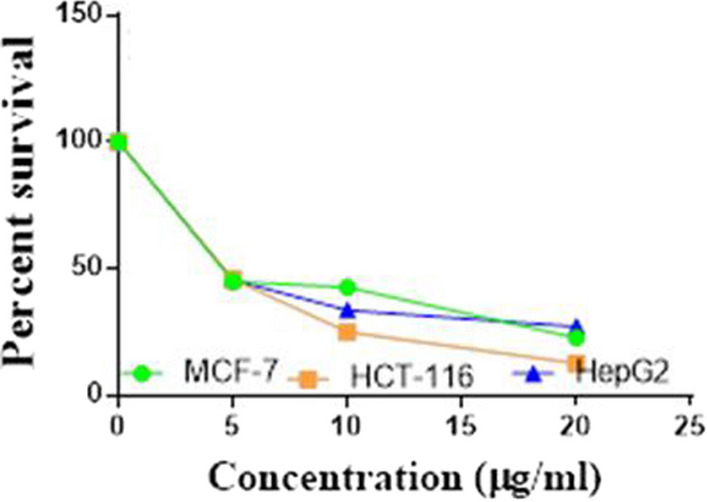
IC_50_ values of **16a**

**Fig. 19:**
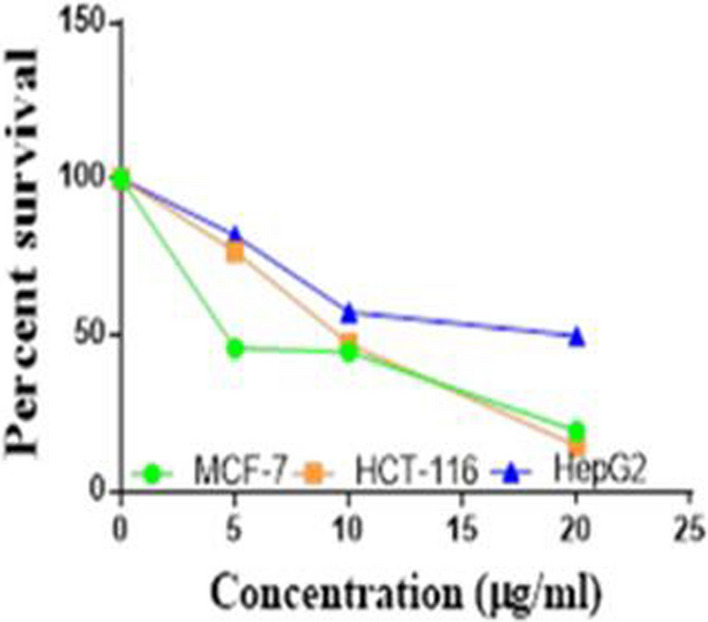
IC_50_ values of **16b**

**Fig. 20:**
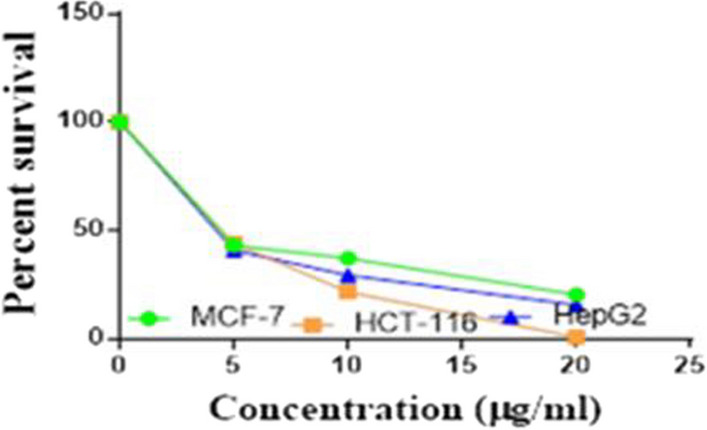
IC_50_ values of **16c**

Compounds **1**, **4a**,**b**, **7a**–**c**, **7k**, **l**, **9a**–**c**, **14a**, **15b**, **16a**–**c** were selected to be investigated against three human cancer cell lines MCF7, HepG2 and HCT116 cell lines using MTT assay using doxorubicin as the standard drug. Each point is the mean ± SD (standard deviation) of three independent experiments performed in triplicate, using the prism software program (integrated Graphpad software, version 3). Cytotoxicity was assessed at concentrations of 5, 10 and 20 µg/l and the IC_50_ values of the tested compounds compared to the reference drug were evaluated as shown in Table 1, 2 and 3. In addition, the percentage of the viable cells was measured and compared with the control (Figs. 5, 6, 7, 8, 9, 10, 11, 12, 13, 14, 15, 16, 17, 18, 19, 20). From the results presented in Table 1, compounds **7b** and **16c** strongest cytotoxic activity against MCF7 with IC_50_ = 3.864 and 3.805 µg/l, respectively, among the tested compounds compared to the doxorubicin (IC_50_ = 2.527 µg/l). Other compounds tested showed moderate to weak cytotoxic activity.

**Table 1:** The IC_50_ values (the drug concentrations that inhibited 50% of cell proliferation) of the compounds on MCF7 cell line

MCF7	20 µg/l	10 µg/l	5 µg/l	IC_50_/µg/l
**Doxo**	98.19	89.14	75.43	2.527
**1**	73.65	61.39	31.71	8.153
**4a**	84.71	74.99	35.61	6.454
**4b**	70.37	55.85	22.19	9.983
**7a**	66.12	45.66	30.13	11.17
**7b**	95.03	79.12	60.48	3.864
**7c**	92.48	69.00	40.12	6.276
**7k**	71.12	55.16	36.19	8.418
**7l**	97.26	52.28	40.19	7.227
**9a**	75.34	49.11	36.10	8.857
**9b**	72.13	51.19	33.82	9.161
**9c**	87.32	53.72	38.17	7.565
**14a**	77.19	48.62	25.14	9.963
**15b**	83.62	62.92	28.52	7.893
**16a**	77.25	57.34	55.09	4.385
**16b**	80.56	55.27	54.13	4.942
**16c**	79.61	62.79	56.81	3.805

**Table 2:** The IC_50_ values (the drug concentrations that inhibited 50% of cell proliferation) of the compounds on HepG2 cell line

HepG2	20 µg/l	10 µg/l	5 µg/l	IC_50_ µg/l
**Doxo**	91.13	68.59	53.46	4.749
**1**	65.34	53.18	43.17	7.818
**4a**	82.16	60.95	15.91	8.998
**4b**	76.43	43.81	38.14	9.155
**7a**	76.04	42.56	38.17	9.348
**7b**	85.16	69.08	55.11	4.250
**7c**	67.89	41.65	35.08	10.930
**7k**	90.19	88.59	41.84	5.521
**7l**	96.62	71.84	40.25	6.117
**9a**	79.17	49.16	13.11	10.570
**9b**	73.11	33.81	8.46	13.22
**9c**	86.55	50.63	42.88	7.257
**14a**	83.75	40.75	11.39	11.27
**15b**	78.66	75.98	49.48	4.641
**16a**	72.80	66.36	54.39	3.555
**16b**	50.18	42.54	18.01	17.650
**16c**	84.32	70.57	59.17	3.427

**Table 3:** The IC_50_ values (the drug concentrations that inhibited 50% of cell proliferation) of the compounds on HCT-116 cell line

HCT116	20 µg/l	10 µg/l	5 µg/l	IC_50_/µg/l
**Doxo**	95.26	77.18	62.14	3.641
**1**	71.08	52.09	16.23	10.81
**4a**	84.77	71.78	56.23	3.966
**4b**	66.23	21.33	7.31	15.73
**7a**	91.78	84.52	50.82	4.866
**7b**	91.94	70.12	67.87	2.487
**7c**	68.85	68.21	50.56	4.072
**7k**	62.92	46.55	39.48	10.25
**7l**	91.59	61.58	57.14	4.457
**9a**	65.98	55.47	34.38	9.109
**9b**	59.80	40.50	22.09	14.15
**9c**	89.80	67.13	59.74	3.778
**14a**	58.72	55.79	28.84	10.99
**15b**	84.20	66.87	26.10	7.754
**16a**	87.26	74.90	54.16	4.369
**16b**	85.28	52.73	23.18	9.174
**16c**	99.23	78.34	55.98	4.503

Furthermore, from screening the cytotoxic activity of the tested compounds against HepG2 cell line, we can infer that, compounds **7b**, **15b**, **16a** and **16c** showed higher potency against the HepG-2 cell line with IC_50_ = 4.250, 4.641, 3.555 and 3.427 µg/l, respectively compared to the reference drug (IC_50_ = 4.749 µg/l). The remaining tested compounds showed moderate to weak activity (Table 2).

Based on the results of the cytotoxic activity of the tested compounds against HCT116 (Table 3), compound **7b** exhibited a higher cytotoxic activity against the HCT116 (IC_50_ = 2.487 µg/l) compared to the doxorubicin (IC_50_ = 3.641 µg/l). Additionally, compounds **7c**, **16a** and **16c** exhibited high anticancer activity against HCT116 with IC_50_ values of 4.072, 4.369 and 4.503 µg/l, respectively. The other rested compounds showed moderate to low activity.

### Structure–activity relationship (SAR)

**Fig. 21:**
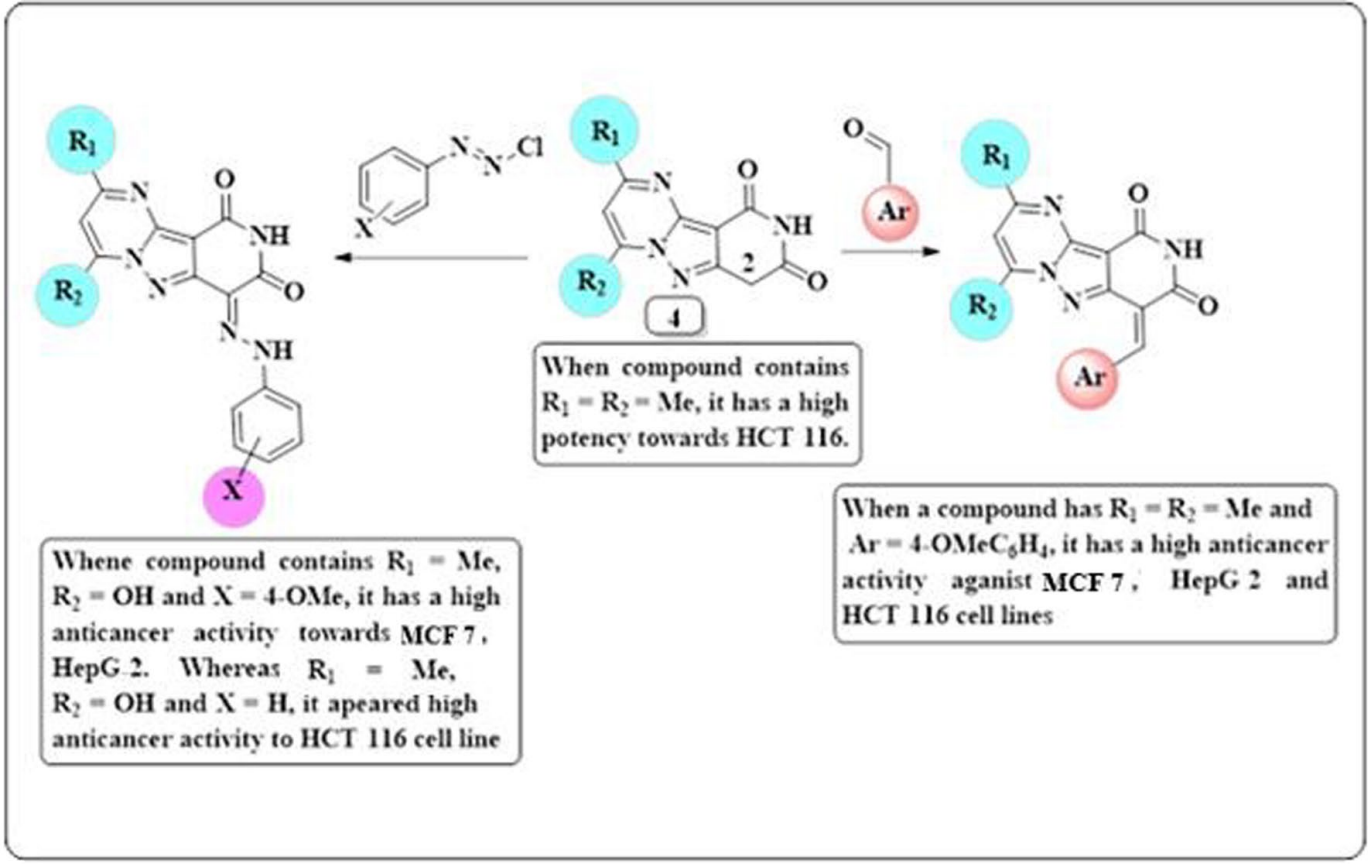
Structure activity relationship (SAR) of some synthesized compounds

The results obtained from the anticancer activity of some newly prepared compounds show the all tested compounds have antitumor activity against all the three cell lines (MCF7, HepG2 and HCT116) (Fig. 21).

Initially, parent compound **1** exhibited a moderate cytotoxicity (IC_50 =_ 7.818 µg/l) against the HepG2 cell line compared to doxorubicin (IC_50_ = 4.749 µg/l). When compound **1** was converted into a tricyclic ring system containing a pyrimidine ring as in compounds **4a**,**b**, the anticancer activity varies depending on the nature of the substituents present on the pyrimidine ring. Therefore, when the substituent of compound **4** is a methyl group like **4a**, the anticancer activity gradually increases towards HCT116 with an IC_50_ of 3.966 µg/l, equivalent to doxorubicin (IC_50_ = 3.641 µg/l). When adding another aryl group to 2nd position of compounds **4a**,**b**, the anticancer activity changes depending on the position of the substituents on the aryl group. Thus, the anticancer activity does not change when the aryl group is a phenyl ring. However, when the methoxy group was introduced as a donor group in the aryl moiety as in **7b**, the anticancer activity increased towards MCF7 (IC_50_ = 3.864 µg/l), HepG2 (IC_50_ = 4.250 µg/l) and HCT116 (IC_50_ = 2.487 µg/l) as shown in Tables 1, 2 and 3. On the other hand, when compounds **7a**,**b** contain a chlorine atom on the aryl group as in the case of **7c**, the anticancer activity was reduced in all three cell lines tested. Coupling of compounds **4a**,**b** with arenediazonium salts afforded arylhydrazo derivatives **9a**–**d** which, have different cytotoxicities depending on the nature of the substituent on the arylhydrazo moiety. Thus, when the arylhydrazo was a phenyl or tolyl group, it had low anticancer activity against all three cell lines, whereas for the aryl moieties containing a methoxy group as donating group like **9c**, the anticancer activity increased especially in the HCT116 cell line with IC_50_ = 3.778 µg/l. Furthermore, when compound **1** was condensed with a *β*-ketoester to form a tricyclic ring system containing a hydroxyl group as in **14a**,**b**, it appeared to have weak anticancer activity. However, when compounds **14a**,**b** have a methoxy group as in **15b**, the anticancer activity was increased against HepG2 with IC_50_ = 4.641 µg/l compared to doxorubicin (IC_50_ = 4.749 µg/l). Also, when compounds **14a**,**b** were coupled with arenediazonium salts to afford arylhydrazo derivatives **16a**–**c**, the anticancer activity was increased in the case of the arylhydrazo group with the methoxy group, as in the case of **16c** (Tables 1, 2, and 3).

### Enzyme inhibition assay

**Fig. 22:**
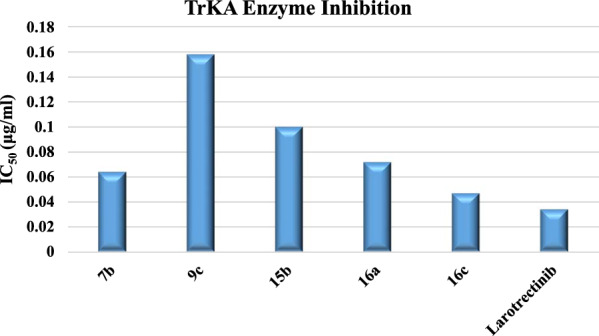
Enzyme inhibition of tested compounds

Compounds **7b**, **9c**, **15b**, **16a** and **16c** with the strongest anticancer activity were tested for tropomyosin kinase A receptor inhibitory activity by a kinase assay technique utilizing Larotrectinib as a positive control. The data listed in Table 4 and Fig. 22 demonstrate that compound **16c** has the strongest inhibitory effect among the tested compounds against to tropomyosin receptor kinase A (TrKA) with IC_50_ = 0.047 ± 0.0027 μg/ml compared to Larotrectinib with IC_50_ = 0.034 ± 0.0021 μg/ml using the HepG2 cancer cell line. While, compounds **7b** and **16a** have moderate activity anti-TrKA with IC_50_ = 0.064 ± 0.0037 and 0.072 ± 0.0042 μg/ml, respectively. In addition, compounds **9c** and **15b** have weak activity against TrKA with IC_50_ = 0.158 ± 0.0092 and 0.101 ± 0.0059 μg/ml, respectively. Therefore, compound **16c** can cause cancer cell line death by inhibiting the enzyme tropomyosin receptor kinase A, possibly because it contains a methoxy group as donating group.

**Table 4:** Inhibitory activity of compounds **7b**, **9c**, **15b**, **16a** and **16c** against tropomyosin receptor kinase A in *vitro* using kinase assay technique

Compound no	Tropomyosin receptor kinase A IC_50_ (μg/ml)
**7b**	0.064 ± 0.0037
**9c**	0.158 ± 0.0092
**15b**	0.101 ± 0.0059
**16a**	0.072 ± 0.0042
**16c**	0.047 ± 0.0027
**Larotrectinib**	0.034 ± 0.0021

### Cell cycle analysis

**Fig. 23:**
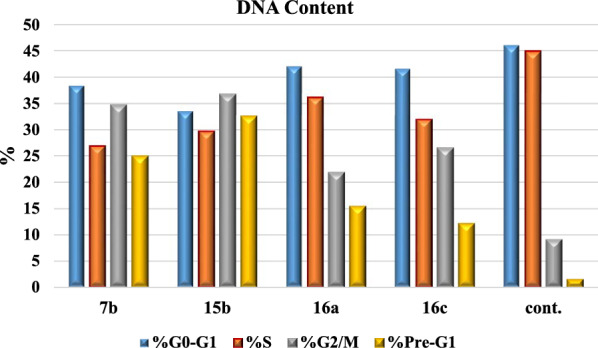
Cell cycle analysis of compounds **7b**, **15b**, **16a** and **16c**. Compounds **7b**, **15b**, **16a** and **16c** increased the ratio of cells at phases in the G2/M by about 4, 4, 2.5 and 3 times, respectively, and HepG2 cells were arrested in the cell cycle at G2/M phase by compounds **7b** and **15b**

**Fig. 24:**
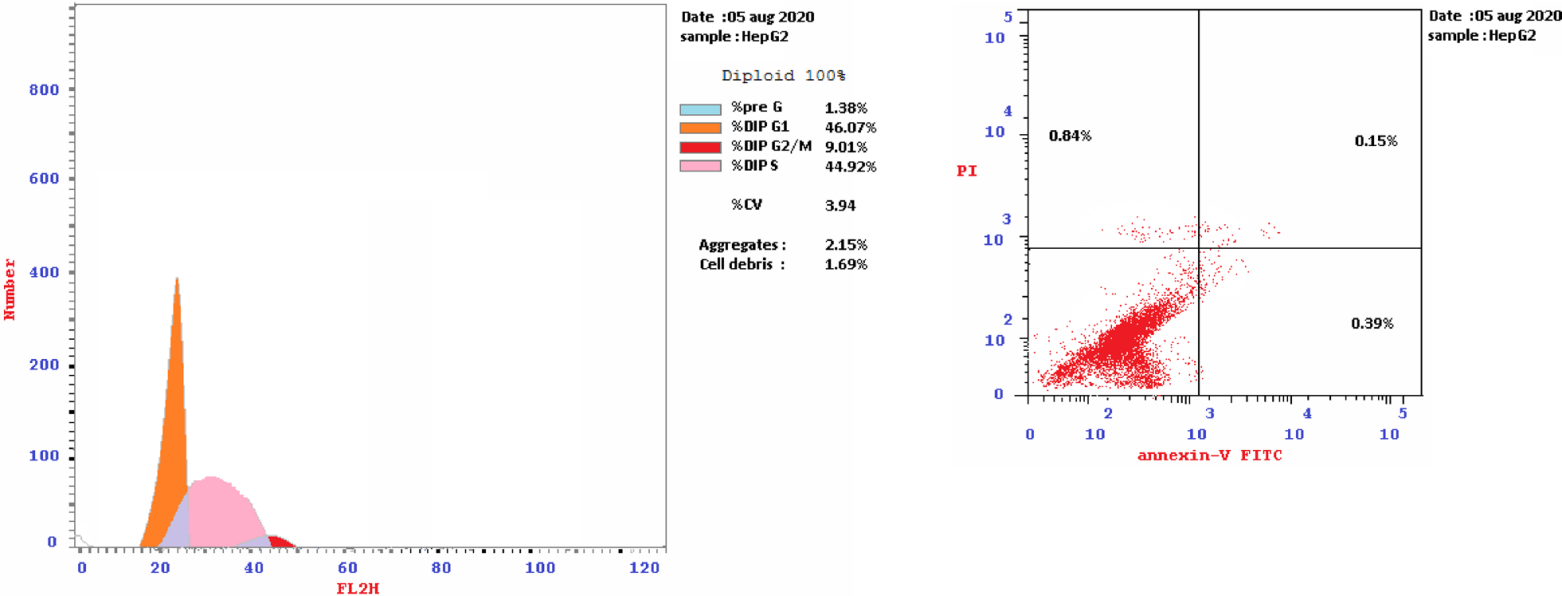
Cell cycle of control HepG-2

**Fig. 25:**
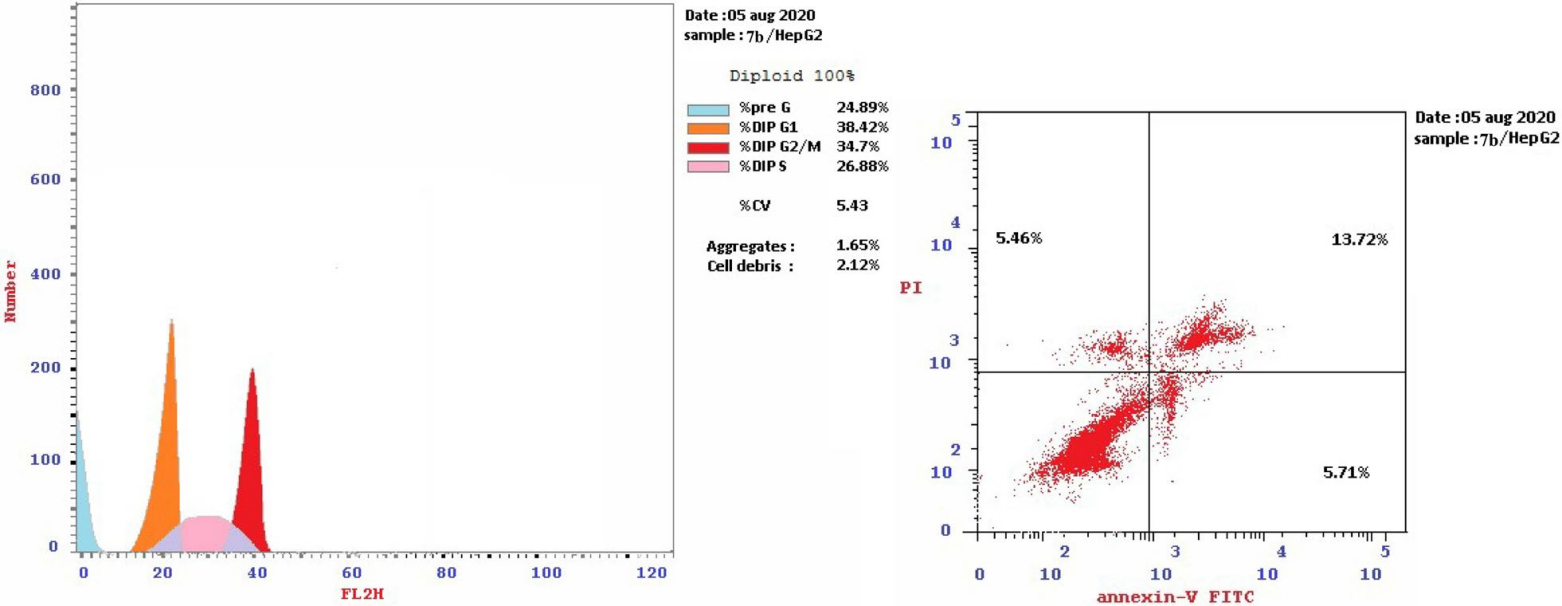
Cell cycle of compound 7b

**Fig. 26:**
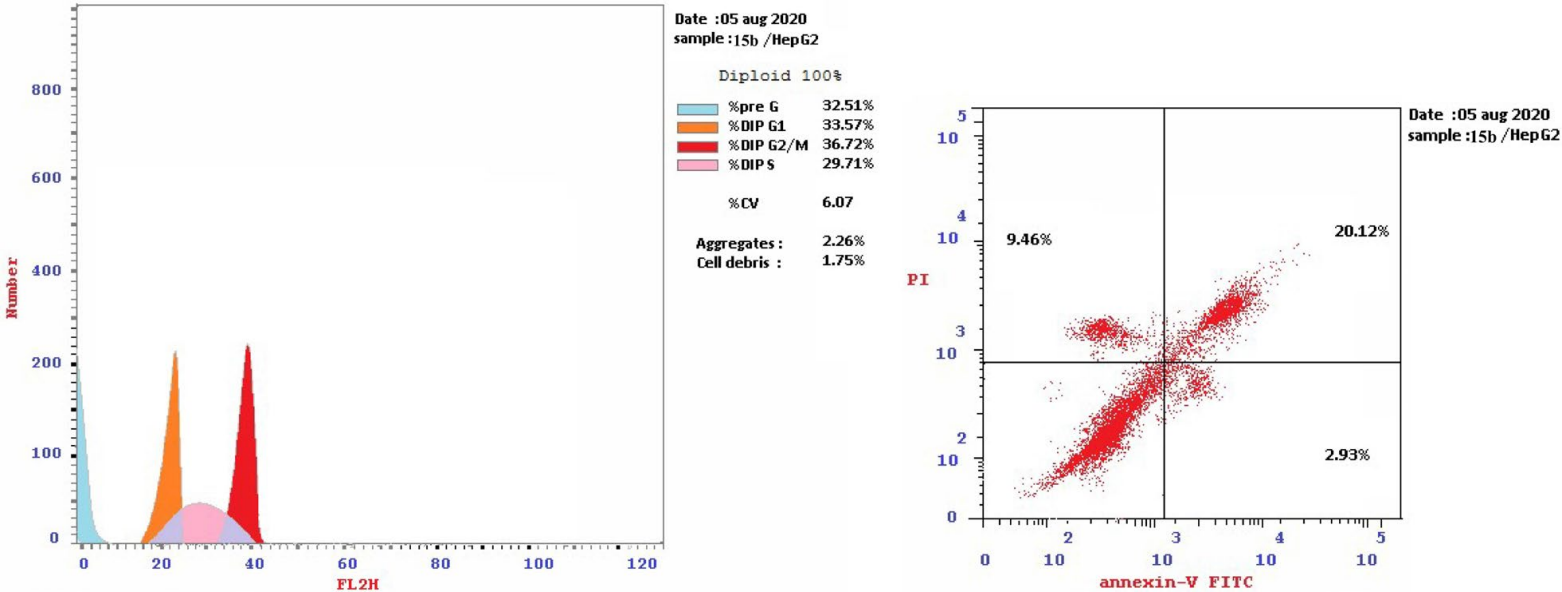
Cell cycle of compound 15b

**Fig. 27:**
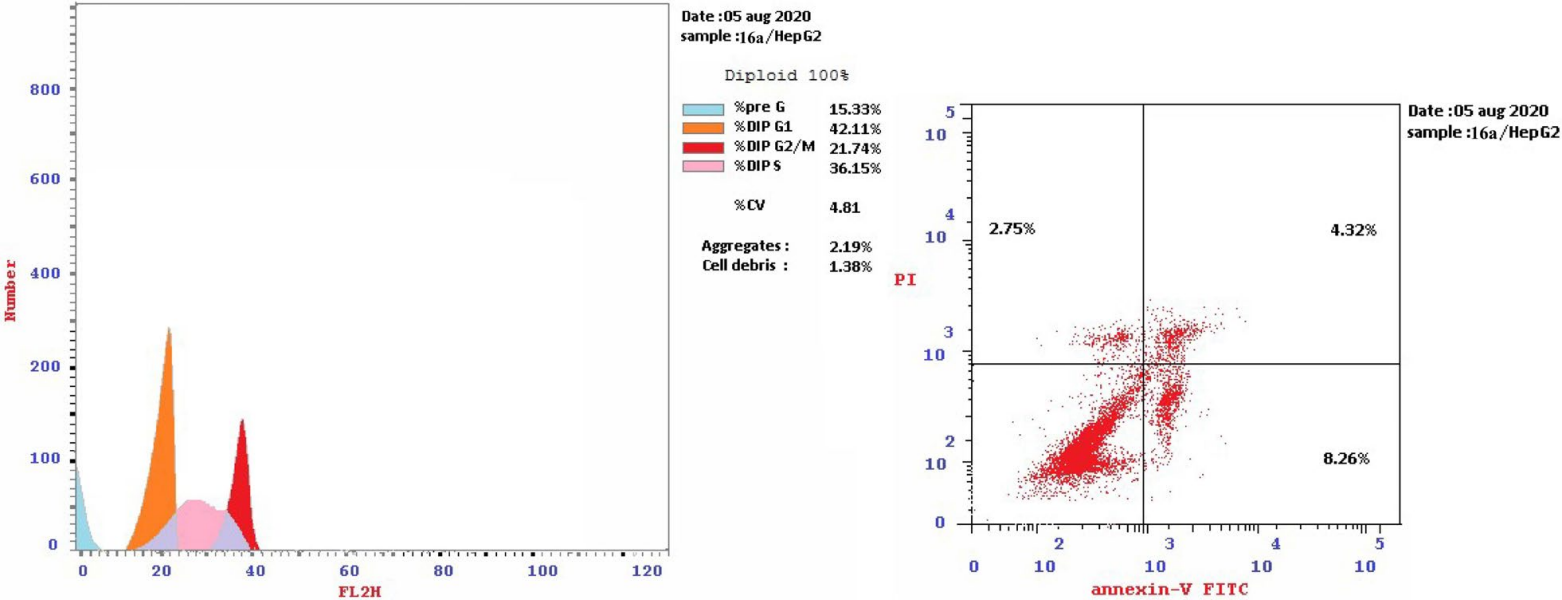
Cell cycle of compound 16a

**Fig. 28:**
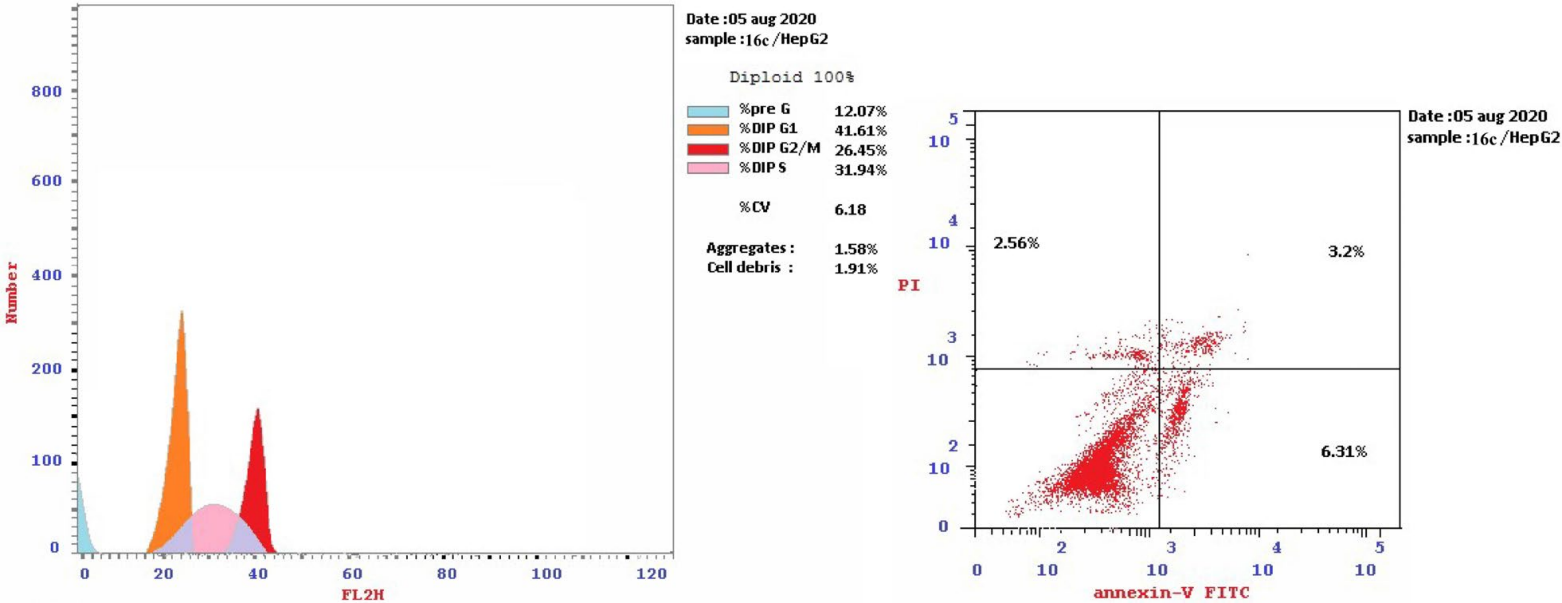
Cell cycle of compound 16c

For cell cycle analysis, stained DNA from HepG2 cancer cells was treated with compounds **7b**, **15b**, **16a** and **16c** that induce cancer cell death by inhibiting TrKA. From the results in Table 5, it can be seen that the proportion of cells at phase in the pre-G1 of compounds **7b**, **15b**, **16a** and **16c** increased the proportion of cells at phase in the G2/M by about 4, 4, 2.5 and 3 folds, respectively. Additionally, HepG2 cells were arrested in the cell cycle at G2/M phase by compounds **7b** and **15b** among the tested compounds **16a** and **16c** (Table 5 and Figs. 23, 24, 25, 26, 27, 28).

**Table 5:** Cell cycle analysis in HepG2 using compounds **7b**, **15b**, **16a** and **16c**

Compound no	Results DNA content				
Code	%G0-G1	%S	%G2/M	%Pre-G1	Comment
**7b**/**HepG2**	38.42	26.88	34.70	24.89	cell growth arrest@G2/M
**15b**/**HepG2**	33.57	29.71	36.72	32.51	cell growth arrest@G2/M
**16a**/**HepG2**	42.11	36.15	21.74	15.33	cell growth arrest@G2/M
**16c**/**HepG2**	41.61	31.94	26.45	12.07	cell growth arrest@G2/M
cont. **HepG2**	46.07	44.92	9.01	1.38	–

### Detection of apoptosis assay

**Fig. 29:**
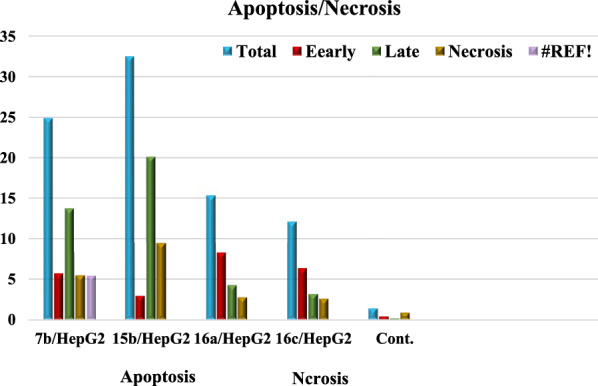
Apoptosis and necrosis of tested compounds **7b**, **15b**, **16a**, **16c** with control

Early and late apoptosis was determined after treatment of HepG2 cells with compounds **7b**, **15b**, **16a** and **16c** compared with untreated control cells. The late apoptosis rate increased by about 13, 20, 4 and 3 times, respectively, showing a higher efficiency than the early apoptosis ratio 5, 2, 8 and 6 times, respectively. Total apoptosis from treatment of HepG2 cells with compound **15b** showed the higher apoptotic induction efficiency compared with other tested compounds **7b**, **16a** and **16c** (Table 6 and Fig. 29).

**Table 6:** The effect of compounds **7b**, **15b**, **16a** and **16c** on HepG2 cell lines

Code	Apoptosis			Necrosis
	Total	Early	Late	
**7b**/**HepG2**	24.89	5.71	13.72	5.46
**15b**/**HepG2**	32.51	2.93	20.12	9.46
**16a**/**HepG2**	15.33	8.26	4.32	2.75
**16c**/**HepG2**	12.07	6.31	3.20	2.56
**cont.HepG2**	1.38	0.39	0.15	0.84

### Molecular docking study

**Fig. 30:**
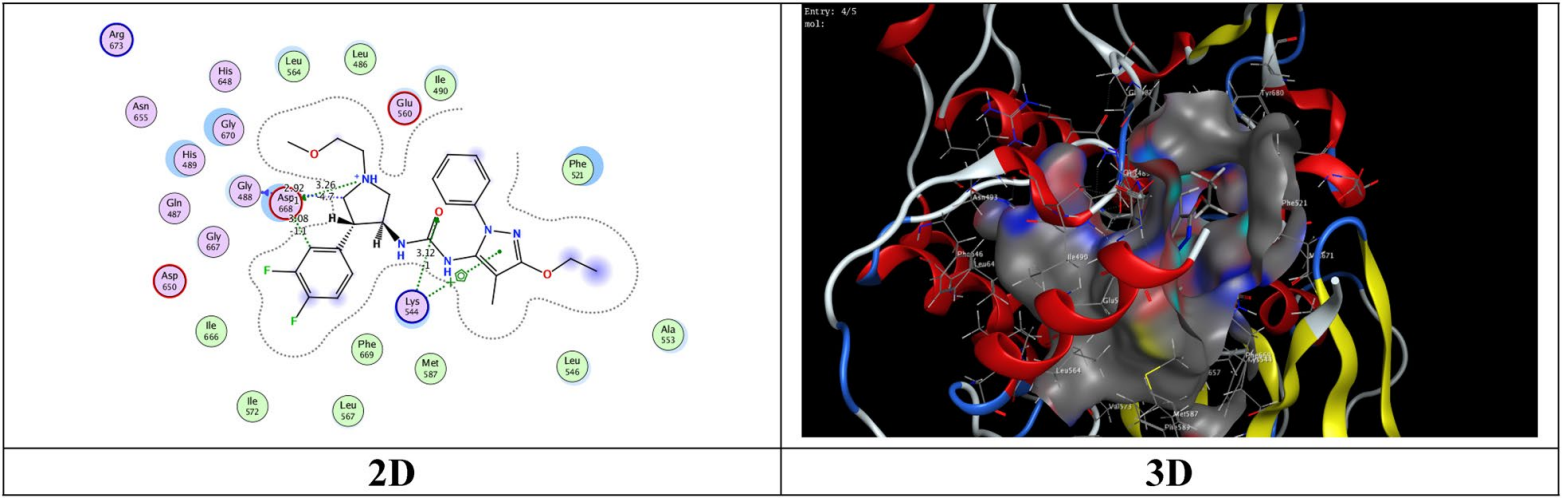
Interaction of 5H3Q with the active site in 2D and 3D

**Fig. 31:**
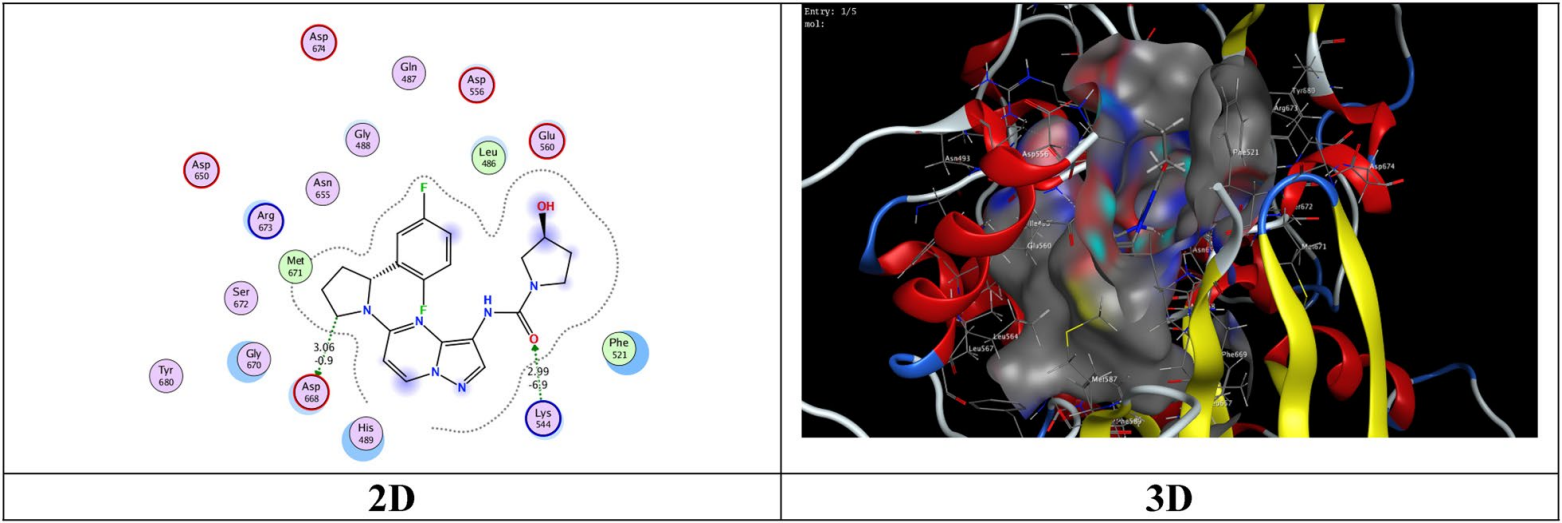
Interaction of larotrectinib with the active site of 5H3Q in 2D and 3D

**Fig. 32:**
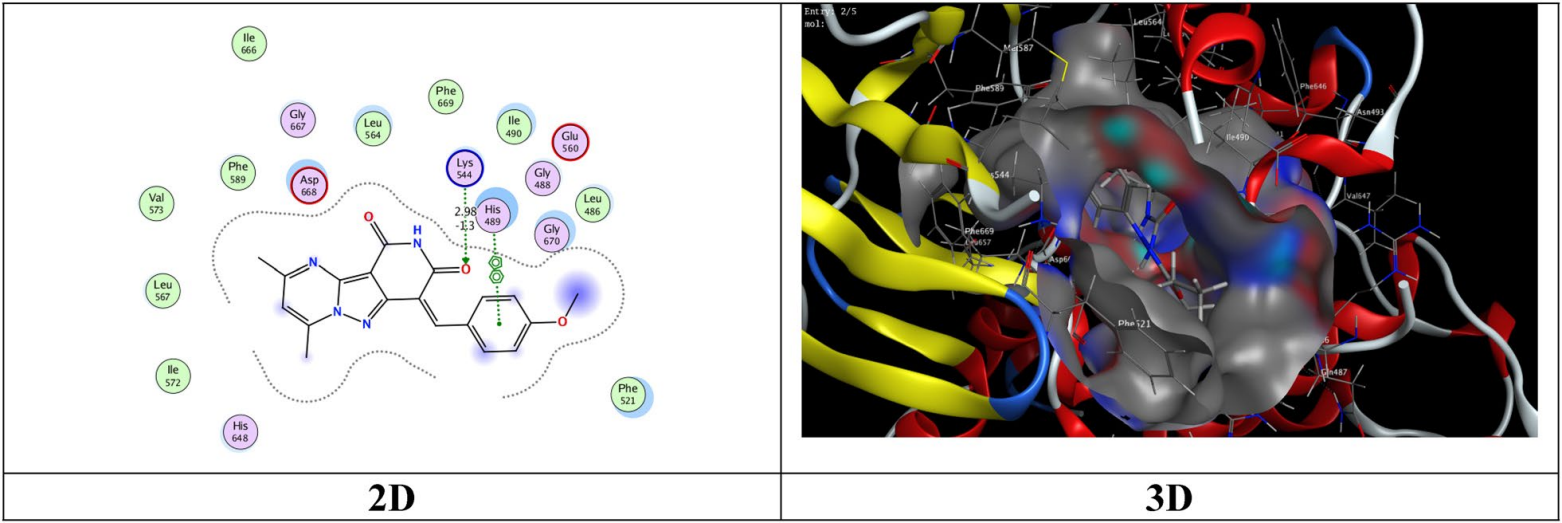
Interaction of **7b** with the active site of 5H3Q in 2D and 3D

**Fig. 33:**
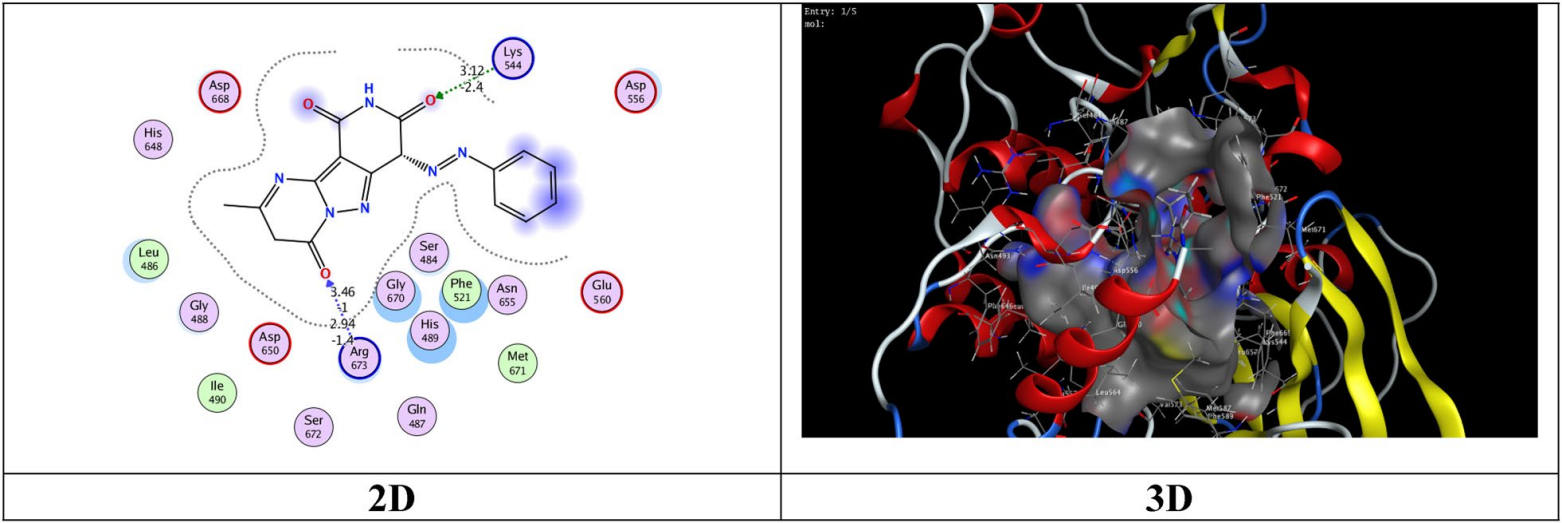
Interaction of **16a** with the active site of 5H3Q in 2D and 3D

**Fig. 34:**
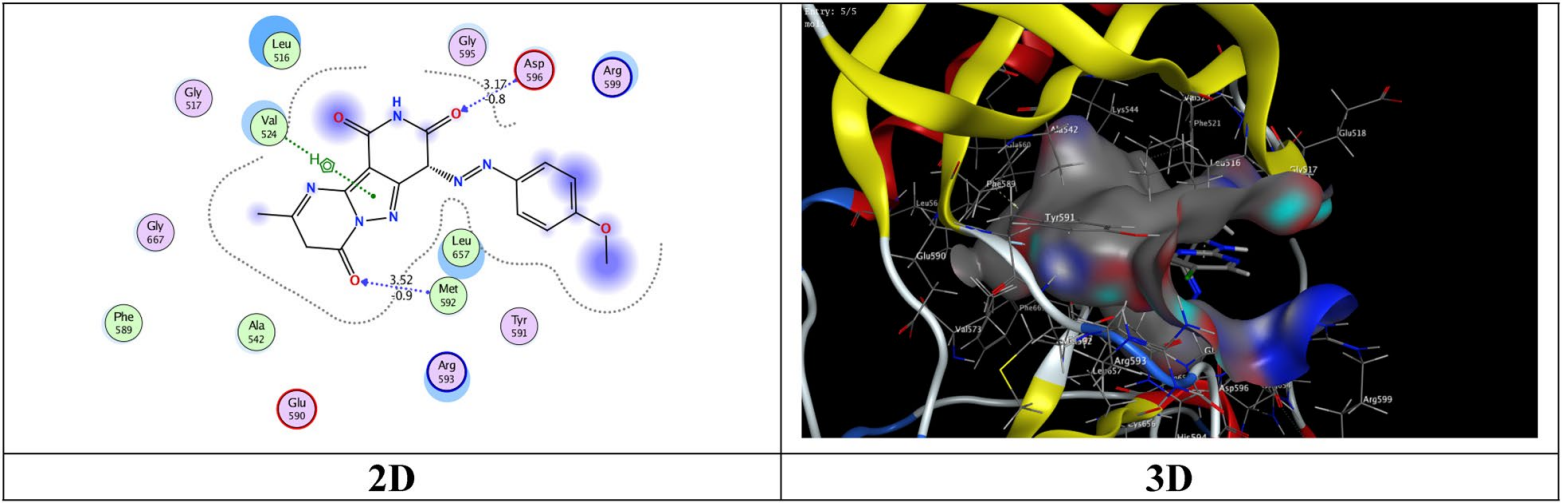
Interaction of **16c** with the active site of 5H3Q in 2D and 3D

The most potent inhibitory compounds **7b**, **16a** and **16c** as well as the standard drug Larotrectinib against TrKA were docked with the crystal structure of tropomyosin receptor kinase A (TrKA) (PDB: 5H3Q, Fig. 30) used the molecular operating environment docking (MOE) 2009 to find the exact binding pattern to the receptor. From the present studies, it was found that all the anchored compounds exhibited good binding energies ranging from − 7.3801 to − 6.5837 kcal mol^−1^ and displayed good fitness with the active site of the 5H3Q protein. Thus, the standard drug Larotrectinib exhibits two hydrogen bond interactions with bond length 2.99Ǻ and 3.06 Ǻ with amino acid residues Lys 544 and Asp 668, respectively and binding energy (S) = − 7.1325 kcal mol^−1^ (Fig. 31). Compound **7b** appears to have a hydrogen bond interaction with a bond length 2.98 Ǻ between the carbonyl function of the pyridine moiety and the amino acid residue Lys 544 as well as a cation-cation interaction between the 4-methoxyphenyl group and His 489 with S = − 7.3801 kcal mol^−1^ (Fig. 32). On the other hand, compound **16a** exhibits a binding energy of S = − 7.0296 kcal mol^−1^ and appears two hydrogen bond interactions with bond length equal to 3.12 and 3.46 Ǻ between the two carbonyl groups of each of pyridine and pyrimidine rings, respectively and the amino acid residues Lys 544 and Arg 673 (Fig. 33). Additionally, compound **16c** the most potent inhibitory activity against TrKA exhibits two hydrogen bond interactions, one between the carbonyl group of the pyrindine ring and Met 507 with bond length of 3.52 Ǻ and the other between the carbonyl group of the pyrimidine ring and Asp 596 with bond length equal to 3.17 Ǻ, as well as a cation-cation bond interaction between pyrazole ring and Val 524 with S = − 7.4667 kcal mol^−1^ (Fig. 34). All data presented from the molecular docking study for larotrectinib, **7b**, **16a**, and **16c** are listed in Table 7.

**Table 7:** Interactions of compounds **7b**, **16a**, **16c**, and **Larotrectinib** with 5H3Q enzyme

Compd. no	B. E. (S) Kcal/mol	Interaction groups	Interaction amino acids	length of hydrogen bonds Ǻ
**Larotrectinib**	− 7.1325	CO CH	Lys 544 Asp 668	2.99 Ǻ 3.06 Ǻ
**7b**	− 7.3801	CO Phenyl ring	Lys 544 His 489	2.98 Ǻ cation-cation
**16a**	− 7.0296	CO CO	Lys 544 Arg 673	3.12 Ǻ 3.46 Ǻ
**16c**	− 6.5837	CO CO Pyrazole ring	Met 587 Asp 596 Val 524	3.52 Ǻ 3.17 Ǻ cation-cation

### In silico ADME studies

In silico prediction of potential pharmacokinetic properties absorption, distribution, metabolism and excretion toxicity (ADME/T) properties calculated using Swiss ADME (http://www.swissadme.ch/) online tools are presented in Table 8. Some physical properties such as absorption, distribution, metabolism, excretion and toxicity are important for any oral drug. Lipinski`s rule of five (RO5), posits that the lipophilicity and solubility are more essential properties than other properties, and rule states that most “drug-like” molecules have log P ≤ 5, molecular weight ≤ 500, number of hydrogen bond acceptors ≤ 10, and number of hydrogen bond donors ≤ 5. Compounds that violate more than one of these rules may have bioavailability problems. According to this rule, the compounds **4a**, **7a**–**c**, **9c**, **15b**, **16a**, **16b** and **16c** have violated all parameters of Lipinski’s rule of five. The results listed in Table 9 show that all the compounds have TPSA values and compounds **4a**, and **7a**–**c** have optimal topological polar surface area (TPSA) of 76.36, 76.36, 85.59, and 76.36 Ǻ^2^, respectively. This means that compounds **4a**, **7a**, **7b** and **7c** are better able of permeate cell membranes and adhere to RO5 and are well absorbed through the gastrointestinal tract. In silico predictions of toxicological properties were determined using the Osiris property explorer program (http://www.prperty explorer-cheminfo.org) online tools is presented in Table 9. In the toxicological profile of the compounds, **9c** and **16c** may exhibit medium tumorigenicity, but compounds **7b**, **9c**, **15b**, **16a** **and** **16c** are high risk in the reproductive system is expected. Additionally, all compounds have no irritant effects. Compounds **4a**, **7a**, and **7c** did not cause the toxicity problems mentioned in the software used in this study. All of the compounds studied have positive drug-likeness values, meaning that they all contained fragments commonly found in commercial drugs (Table 9).

**Table 8:** Important pharmacokinetic parameters for bioavailability of compounds **4a**, **7a**, **7b**, **7c**, **9c**, **15b**, **16a**, **16b** and **16c**

Compd. no	M. Wt g/mol	LogP	TPSA	GI abs	HBA	HBD	nRotb	Violations
**4a**	230.22	0.63	76.36 Ǻ^2^	high	4	1	0	0
**7a**	318.33	2.21	76.36 Ǻ2	high	4	1	0	0
**7b**	348.36	2.22	85.59 Ǻ^2^	high	5	1	2	0
**7c**	352.77	2.78	76.36 Ǻ2	high	4	1	1	0
**9c**	364.36	1.88	113.74 Ǻ^2^	high	6	2	3	0
**15b**	350.33	1.68	105.82 Ǻ^2^	high	6	2	2	0
**16a**	336.30	1.34	124.74 Ǻ^2^	high	6	3	2	0
**16b**	350.33	1.67	124.74 Ǻ2	high	6	3	2	0
**16c**	366.33	1.30	133.97 Ǻ^2^	high	7	3	3	0

**Table 9:** Important toxicity predication of compounds **4a**, **7a**, **7b**, **7c**, **9c**, **15b**, **16a**, **16b** and **16c**

Compd. no	Mutag	Tum	Irr	Repd	Drug likeness	Drug Score
**4a**	No	No	No	No	6.35	0.98
**7a**	No	No	No	No	5.04	0.94
**7b**	No	No	No	high	6.26	0.55
**7c**	No	No	No	No	6.77	0.91
**9c**	No	medium	No	high	5.44	0.43
**15b**	No	No	No	high	6.27	0.55
**16a**	No	high	No	high	5.56	0.33
**16b**	No	No	No	high	5.41	0.54
**16c**	No	medium	No	high	5,46	0.43

*Mutag* Mutagenicity, *Tum* Tumorigenicity, *Irr* Irritating effects, *Repd* Reproductive effects

**Fig. 35:**
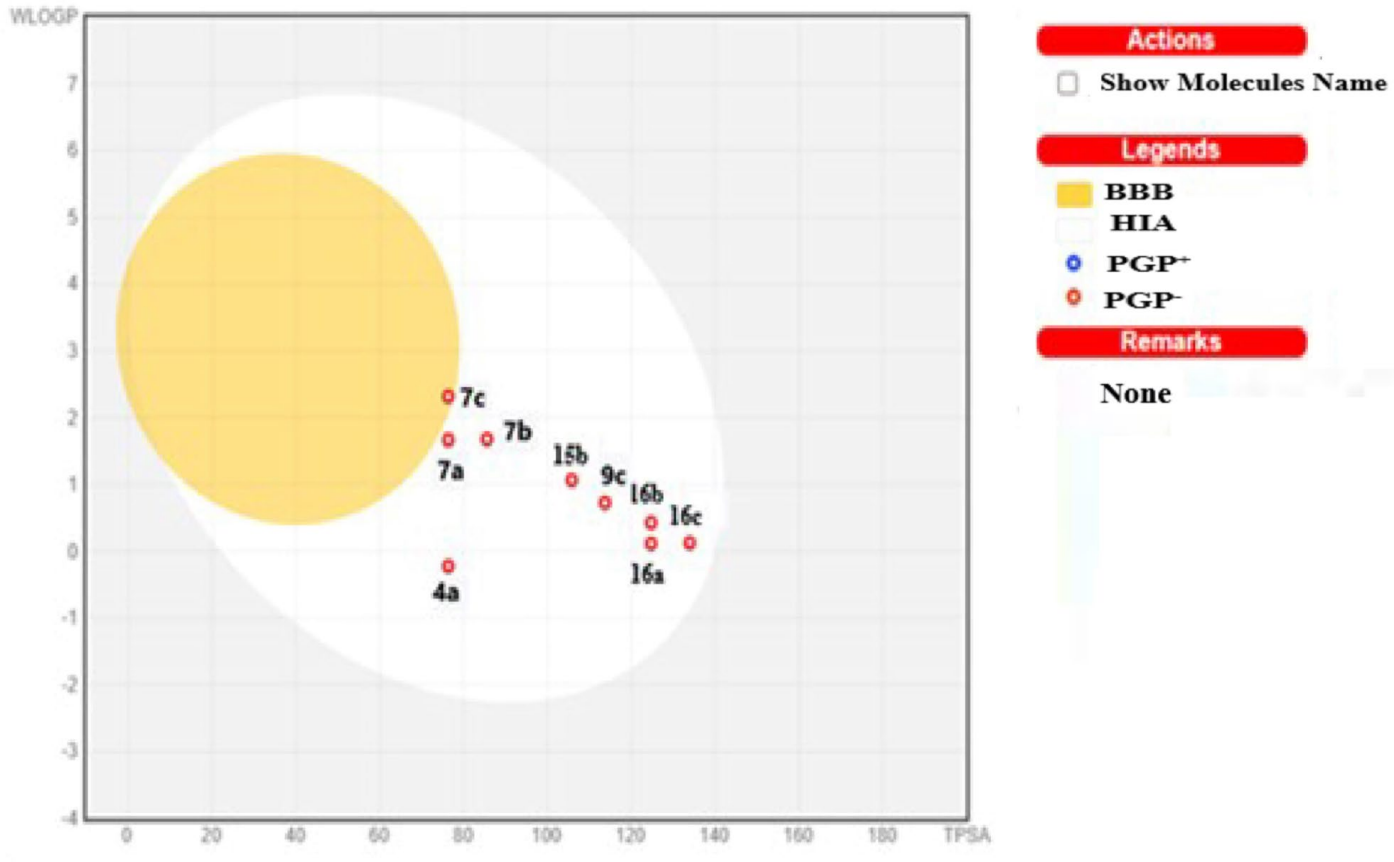
Boiled-egg depicts gastrointestinal absorption and brain penetration of compounds **4a**, **7a**–**c**, **9c**, **15b** and **16a**–**c**

Swissadme helps us provide information on poorly and highly absorbed drugs to model passive intestinal absorption through the human intestinal tract. Graphical prediction of intestinal absorption and blood–brain barrier permeation of the most potent anticancer activity compounds **4a**, **7a**, **7b**, **7c**, **9c**, **15b**, **16a**, **16b** and **16c** against MCF7, HepG2 and HCT116 are shown in Fig. 35. Boiled egg diagram showing the bioavailability property space for wlog P and TPSA [white area means that intestinal absorption; The yellow area means it has entered the brain well, the intestinal are well absorbed; and the gray area means the intestinal have poor absorption]. This provides a simple visual cue to profile new compounds for their oral absorption. All compounds studied were found in the white region. Additionally, PGP^+^ (substrate) and PGP^−^ (non-substrate) are denoted by blue and red dots for molecules predicated to be CNS efflux or not efflux by P-glycoprotein, respectively. Therefore, all the studied compounds **4a**, **7a**, **7b**, **7c**, **9c**, **15b**, **16a**, **16b** and **16c** are not substrates of P-gp (PGP-), hence, we can say that these compounds have good bioavailability. In this series, compound **7c** gave BBB and a low TPSA of 76.36. This suggests that the molecule can be absorbed very easily through the gastrointestinal tract and preferentially acts as a hydrophobic agent and can be easily transported across the blood–brain barrier.

## Conclusion

In this study, a novel series of pyrido[4ʹ,3ʹ:3,4]pyrazolo[1,5-*a*]pyrimidine derivatives were synthesized. The anticancer activity of these compounds was tested on MCF7, HepG2 and HCT116 cell lines in comparison to doxorubicin. The results showed that some of the synthesized compounds have significant cytotoxic activity. Compound **7b** exhibited high and broad spectrum anticancer activity against all cell lines tested. TrKA inhibition assays on **7b**, **9c**, **15b**, **16a** and **16c** showed a decrease in TrKA expression with IC_50_ values below 0.2 μg/ml. The most potent anticancer targets were examined for their effects on cell cycle distribution and apoptosis induction. The results revealed that **7b** and **15b** induced arrest at the G2/M phase of the cell cycle in HepG2 cells among the other tested compounds. Furthermore, docking studies revealed that **7b**, **16a** and **16**c bind with high affinity to the active site of TrKA. In addition, compounds **7b**, **15b**, **16a** and **16c** appear to be well absorbed from the gastrointestinal tract. These results suggest that these compounds may be a promising tools for the production of more potent anticancer agents.

### Supplementary Information

**Additional file 1: Figure S1.** Mass spectrum of compound 2. **Figure S2.** IR spectrum of compound 2. **Figure S3.** ^1^H NMR spectrum of compound 2. **Figure S4.** Mass spectrum of compound 4a. **Figure S5.** IR spectrum of compound 4a. **Figure S6.** ^1^H NMR spectrum of compound 4a. **Figure S7.** ^13^C NMR spectrum of compound 4a. **Figure S8.** Mass spectrum of compound 4b. **Figure S9.** ^1^H NMR spectrum of compound 4b. **Figure S10.** ^1^H NMR spectrum of compound 7a. **Figure S11.** ^13^C NMR spectrum of compound 7a. **Figure S12.** ^1^H NMR spectrum of compound 7b. **Figure S13.** ^1^H NMR spectrum of compound 7f. **Figure S14.** ^13^C NMR spectrum of compound 7f. **Figure S15.** IR spectrum of compound 7g. **Figure S16.** ^1^H NMR spectrum of compound 7g. **Figure S17.** ^1^H NMR spectrum of compound 7h. **Figure S18.** IR spectrum of compound 7i. **Figure S19.** ^1^HNMR spectrum of compound 7i. **Figure S20.** IR spectrum of compound 7j. **Figure S21.** ^1^H NMR spectrum of compound 7k. **Figure S22.** ^13^C NMR spectrum of compound 7k. **Figure S23.** ^1^HNMR spectrum of compound 7m. **Figure S24.** IR spectrum of compound 7n. **Figure S25.** ^1^HNMR spectrum of compound 7n. **Figure S26.** IR spectrum of compound 7o. **Figure S27.** ^1^HNMR spectrum of compound 7o. **Figure S28.** IR spectrum of compound 7p. **Figure S29.** ^1^HNMR spectrum of compound 7p. **Figure S30.** ^13^C NMR spectrum of compound 7q. **Figure S31.** ^1^H NMR spectrum of compound 7r. **Figure S32.** ^1^H NMR spectrum of compound 7s. **Figure S33.** ^1^H NMR spectrum of compound 7t. **Figure S34.** IR spectrum of compound 9a. **Figure S35.** IR spectrum of compound 9b. **Figure S36.** Mass spectrum of compound 9b. **Figure S37.** MS spectrum of compound 9c. **Figure S38.** IR spectrum of compound 9d. **Figure S39.** IR spectrum of compound 11a. **Figure S40.** ^1^H NMR spectrum of compound 11a. **Figure S41.** ^1^H NMR spectrum of compound 11b. **Figure S42.** ^1^H NMR spectrum of compound 11c. **Figure S43.** Mass spectrum of compound 11d. **Figure S44.** Mass spectrum of compound 14a. **Figure S45.** IR spectrum of compound 14a. **Figure S46.** Mass spectrum of compound 14b. **Figure S47.** IR spectrum of compound 14b. **Figure S48.** ^1^H NMR spectrum of compound 14b. **Figure S49.** ^1^H NMR spectrum of compound 14b (D_2_O). **Figure S50.** ^13^C NMR spectrum of compound 15a. **Figure S51.** ^1^H NMR spectrum of compound 15b. **Figure S52.** IR spectrum of compound 15c. **Figure S53.** ^1^H NMR spectrum of compound 15c. **Figure S54.** ^13^C NMR spectrum of compound 15e. **Figure S55.** ^1^H NMR spectrum of compound 15f. **Figure S56.** MS spectrum of compound 16a. **Figure S57.** IR spectrum of compound 16b. **Figure S58.** ^1^H NMR spectrum of compound 16b. **Figure S59.** MS spectrum of compound 16c. **Figure S60.** IR spectrum of compound 16d. **Figure S61.** ^1^H NMR spectrum of compound 16d. **Figure S62.** IR spectrum of compound 16f. **Figure S63.** ^13^C NMR spectrum of compound 16g. Biological methods for MTT assay, Tropomyosm receptor kinase A inhibitory assay and Annexin-VFITC apoptosis assay were discussed in details in ESI.

## Data Availability

The datasets used and/or analyzed during the present study available from the electronic additional file.

## References

[1] Amatu A, Sartore-Bianchi A, Bencardino K, Pizzutilo GE, Tosi F, Siena S (2019). Tropomyosin receptor kinase (TRK) biology and the role of NTRK gene fusions in cancer. Ann Oncol.

[2] Hechtman JF (2022). NTRK insights: best practices for pathologists. Mod Pathol.

[3] Jin W (2020). Cancers. Roles of TrkC signaling in the regulation of tumorigenicity and metastasis of cancer. Cancers.

[4] Yakes FM, Chen J, Tan J, Yamaguchi K, Shi Y, Yu P, Qian F, Chu F, Bentzien F, Cancilla B, Orf J, You A, Laird AD, Engst S, Lee L, Lesch J, Chou YC, Joly AH (2011). Cabozantinib (XL184), a novel MET and VEGFR2 inhibitor, simultaneously suppresses metastasis, angiogenesis, and tumor growth. Mol Cancer Ther.

[5] Patwardhan PP, Ivy KS, Musi E, de Stanchina E, Schwartz GK (2016). Significant blockade of multiple receptor tyrosine kinases by MGCD516 (Sitravatinib), a novel small molecule inhibitor, shows potent anti-tumor activity in preclinical models of sarcoma. Oncotarget.

[6] Smith BD, Kaufman MD, Leary CB, Turner BA, Wise SC, Ahn YM, Booth RJ, Caldwell TM, Ensinger CL, Hood MM, Lu WP, Patt TW, Patt WC, Rutkoski TJ, Samarakoon T, Telikepalli H, Vogeti L, Vogeti S, Yates KM, Chun L, Stewart LJ, Clare M, Flynn DL (2015). Altiratinib inhibits tumor growth, invasion, angiogenesis, and microenvironment-mediated drug resistance via balanced inhibition of MET, TIE2, and VEGFR2. Mol Cancer Ther.

[7] Smith BD, Leary CB, Turner BA, Kaufman MD, Wise SC, Rendueles MERG, Fagin JA, Flynn DL (2015). Altiratinib is a potent inhibitor of TRK kinases and is efficacious in TRK-fusion driven cancer models. Cancer Res.

[8] Cocco E, Scaltriti M, Drilon A (2018). NTRK fusion-positive cancers and TRK inhibitor therapy Nat. Rev Clin Oncol.

[9] Drilon A, Nagasubramanian R, Blake JF, Ku N, Tuch BB, Ebata K, Smith S, Lauriault V, Kolakowski GR, Brandhuber BJ, Larsen PD, Bouhana KS, Winski SL, Hamor R, Wu WI, Parker A, Morales TH, Sullivan FX, DeWolf WE, Wollenberg LA, Gordon PR, Douglas-Lindsay DN, Scaltriti M, Benayed R, Raj S, Hanusch B, Schram AM, Jonsson P, Berger MF, Hechtman JF, Taylor BS, Andrews S, Rothenberg SM, Hyman DM (2017). A next-generation TRK kinase inhibitor overcomes acquired resistance to prior TRK kinase inhibition in patients with TRK fusion–positive solid tumors. Cancer Discov.

[10] Yan W, Lakkaniga NR, Carlomagno F, Santoro M, McDonald NQ, Lv F, Gunaganti N, Frett B, Li HY (2019). Insights into current tropomyosin receptor kinase (TRK) inhibitors: development and clinical application. J Med Chem.

[11] Vaishnavi A, Le AT, Doebele RC (2015). TRKing down an old oncogene in a new era of targeted therapy. Cancer Discov.

[12] Ricciuti B, Genova C, Crino L, Libra M, Leonardi GC (2019). Antitumor activity of larotrectinib in tumors harboring NTRK gene fusions: a short review on the current evidence. Onco Targets and Therapy.

[13] FDA.gov (2018). FDA approves larotrectinib for solid tumors with NTRK gene fusions. http://www.fda.gov/drug/fda-approves-larotrectinib-solid-tumors-ntrk-gene-fusions.

[14] Berger S, Martens UM, Bochum S (2019). In small molecules in oncology.

[15] FDA.gov (2019). FDA approves entrectinib for NTRK solid tumors and ROS-1 NSCLC. http://www.fda.gov/drugs/resources-information-approved-drugs/fda-approves-entrectinib-ntrk-solid-tumors-and-ros-1-nsxlc.

[16] Dar AC, Shokat KM (2011). The evolution of protein kinase inhibitors from antagonists to agonists of cellular signaling. Annu Rev Biochem.

[17] Gouda MA, Berghot MA, Shoeib AI, Khalil AM (2010). Synthesis and antimicrobial of new anthraquinone derivatives incorporating pyrazole moiety. Eur J Med Chem.

[18] Gopalsamy A, Yang H, Ellingboe JW, Tsou HR, Zhang N, Honores E, Powell D, Miranda M, McGinnis JP, Rabindran SK (2005). Pyrazolo[1,5-*a*]pyrimidin-7-yl phenyl amides as novel antiproliferative agents: Exploration of core and headpiece structure–activity relationships. Bioorg Med Chem Lett.

[19] Ahmed OM, Mohamed MA, Ahmed RR, Ahmed SA (2009). Synthesis and antitumor activities of some new pyridines and pyrazolo[1,5-*a*]pyrimidines. Eur J Med Chem.

[20] Hassan AS, Hafez TS, Osman SAM, Ali MM (2015). Synthesis and in vitro cytotoxic activity of novel pyrazolo[1,5-a]pyrimidines and related Schiff bases Turk. J Chem.

[21] Paruch K, Dwyer MP, Alvarez C, Brown C, Chen TY, Doll RJ, Keertikar K, Knutson C, McKittrick B, Rivera J, Rossman R, Tucker G, Fischmann T, Hruza A, Madison V, Nomeir AA, Wang Y, Kirschmeier P, Lees E, Parry D, Sqambellone N, Seqhezzi W, Schultz L, Shanahan F, Wiswell D, Xu X, Zhou Q, James RA, Paradkar VM, Park H, Rokosz LR, Stauffer TM, Guzi T (2010). Discovery of dinaciclib (SCH 727965): a potent and selective inhibitor of cyclin-dependent kinases. ACS Med Chem Lett.

[22] Metwally NH, Badawy MA, Okpy DS (2015). Synthesis and anticancer activity of some new thiopyrano[2,3-*d*]thiazoles incorporating pyrazole moiety Chem. Pharm Bull.

[23] Metwally NH, Abdelrazek FM, Eldaly SM (2016). Synthesis and anticancer activity of some new heterocyclic compounds based on 1-cyanoacetyl-3,5-dimethylpyrazole Res. Chem Intermed.

[24] Metwally NH, Dee EA (2018). Synthesis, assessment on human breast, liver and colon cell lines and molecular modeling study using novel pyrazolo[4,3-*c*]pyridine derivatives. Bioorg Chem.

[25] Metwally NH, Abdelrazek FM, Eldaly SM (2018). Synthesis, molecular docking, and biological evaluation of some novel bis-heterocyclic compounds based *N, *N-([1,1`biphenyldiyl])bis(2-cyanoacetamide) as potential anticancer agents. J Heter Chem.

[26] Metwally NH, Radwan IT, El-Serwy WS, Mohamed MA (2019). Design, synthesis, DNA assessment and molecular docking study of novel 2-(pyridine-2-ylimino)thiazolidin-4-one derivatives as potent antifungal agents. Bioorg Chem.

[27] Metwally NH, Mohamed SM, Ragb EA (2019). Design, synthesis, anticancer evaluation, molecular docking and cell cycleanalysis of 3-methyl-4,7-dihydropyrazolo[1,5-*a*]pyrimidine derivatives as potent histone lysine demethylases (KDM) inhibitors and apoptosis inducers. Bioorg Chem.

[28] Metwally NH, Mohamed MS (2019). New imidazolone derivatives comprising a benzoate or sulfonamide moiety as anti-inflammatory and antibacterial inhibitors: design, synthesis, selective COX-2. DHFR and molecular-modeling study Bioorg Chem.

[29] Metwally NH, Abdallah SO, Abdel Mohsen MM (2020). Design, green one-pot synthesis and molecular docking study of novel *N, *N-bis(cyanoacetyl)hydrazines and bis-coumarins as effective inhibitors of DNA gyrase and topoisomerase IV. Bioorg Chem.

[30] Metwally NH, Mohamed MS, Deeb EA (2021). Synthesis, anticancer evaluation, DK2 inhibition, and apoptotic activity assessment with molecular docking modeling of new class of pyrazolo[1,5-*a*]pyrimidines. Res Chem Intermed.

[31] Metwally NH, Abd-Elmoety AS (2022). Novel fluorinated pyrazolo[1,5-*a*]pyrimidines: In a way from synthesis and docking studies to biological evaluation. J Mol Struct.

[32] Metwally NH, Badawy MA, Okpy DS (2022). Synthesis, biological evaluation of novel thiopyrano[2,3-*d*]thiazoles incorporating arylsulfonate moiety as potential inhibitors of tubulin polymerization, and molecular modeling studiesJ Mol. Struct.

[33] Metwally NH, El-Desoky EA. Novel thiopyrano[2,3-*d*]thiazole-pyrazole hybrids as potential nonsulfonamide human carbonic anhydrase IX and XII inhibitors: design, synthesis, and biochemical studies. ACS Omega, 2023 in press. 10.1021/acsomega.2c06954 PMC9933482 36816682

[34] Sato T (1959). Reaction of hydrazine hydrate and phenylhydrazine with malononitrile. J Org Chem.

[35] Mosmann T, Immunol J (1983). Rapid colorimetric assay for cellular growth and survival: application to proliferation and cytotoxicity assays. Methods.

[36] Denizot F, Lang R, Immunol J (1986). Rapid colorimetric assay for cell growth and survival. Modifications to the tetrazolium dye procedure giving improved sensitivity and reliability. Methods.

[37] Lipinski CA, Lombardo F, Dominy BW, Feeney PJ (1997). Experimental and computational approaches toestimate solubility and permeability in drug discovery and development settings. Adv Drug Deliv Rev.

[38] Daina A, Zoete V (2016). A BOILED-Egg to predict gastrointestinal absorption and brain penetration of small molecules. Chem Med Chem.

[39] Metwally NH, Deeb EA (2018). Aminopyrazolo[4,3-c]pyridine-4,6-dione as a precursor for novel pyrazolo[4,5,1-*ij*][1,6]naphtyridines and pyrido[4`,3`:3,4]pyrazolo[1,5-*a*]. Synth Commun.

