# Supplementary material for: Synthesis, anticancer evaluation, molecular docking and ADME study of novel pyrido[4ʹ,3ʹ:3,4]pyrazolo[1,5-a]pyrimidines as potential tropomyosin receptor kinase A (TrKA) inhibitors
Source: BMC Chem. 2024 Apr 6;18(1):68. doi: 10.1186/s13065-024-01166-7 (PMC10999085; doi:10.1186/s13065-024-01166-7)
Supplement: Supplementary file 1 — Additional file 1: Figure S1. Mass spectrum of compound 2. Figure S2. IR spectrum of compound 2. Figure S3. 1H NMR spectrum of compound 2. Figure S4. Mass spectrum of compound 4a. Figure S5. IR spectrum of compound 4a. Figure S6. 1H NMR spectrum of compound 4a. Figure S7. 13C NMR spectrum of compound 4a. Figure S8. Mass spectrum of compound 4b. Figure S9. 1H NMR spectrum of compound 4b. Figure S10. 1H NMR spectrum of compound 7a. Figure S11. 13C NMR spectrum of compound 7a. Figure S12. 1H NMR spectrum of compound 7b. Figure S13. 1H NMR spectrum of compound 7f. Figure S14. 13C NMR spectrum of compound 7f. Figure S15. IR spectrum of compound 7g. Figure S16. 1H NMR spectrum of compound 7g. Figure S17. 1H NMR spectrum of compound 7h. Figure S18. IR spectrum of compound 7i. Figure S19. 1HNMR spectrum of compound 7i. Figure S20. IR spectrum of compound 7j. Figure S21. 1H NMR spectrum of compound 7k. Figure S22. 13C NMR spectrum of compound 7k. Figure S23. 1HNMR spectrum of compound 7m. Figure S24. IR spectrum of compound 7n. Figure S25. 1HNMR spectrum of compound 7n. Figure S26. IR spectrum of compound 7o. Figure S27. 1HNMR spectrum of compound 7o. Figure S28. IR spectrum of compound 7p. Figure S29. 1HNMR spectrum of compound 7p. Figure S30. 13C NMR spectrum of compound 7q. Figure S31. 1H NMR spectrum of compound 7r. Figure S32. 1H NMR spectrum of compound 7s. Figure S33. 1H NMR spectrum of compound 7t. Figure S34. IR spectrum of compound 9a. Figure S35. IR spectrum of compound 9b. Figure S36. Mass spectrum of compound 9b. Figure S37. MS spectrum of compound 9c. Figure S38. IR spectrum of compound 9d. Figure S39. IR spectrum of compound 11a. Figure S40. 1H NMR spectrum of compound 11a. Figure S41. 1H NMR spectrum of compound 11b. Figure S42. 1H NMR spectrum of compound 11c. Figure S43. Mass spectrum of compound 11d. Figure S44. Mass spectrum of compound 14a. Figure S45. IR spectrum of compound 14a. Figure S46. Mass spectrum of compound 14b. Figure S47. IR spectrum of comp [file 13065_2024_1166_MOESM1_ESM.docx]

**Journal of BMC Chemistry**

**Additional file 1**

**Synthesis, anticancer evaluation, molecular docking and ADME study of novel pyrido[4`,3`:3,4]pyrazolo[1,5-*a*]pyrimidines as potential tropomyosin receptor kinase A (TrKA) inhibitors**

Nadia Hanafy Metwally*, Emad Abdullah Deeb, Ibrahim Walid Hasani

Chemistry Department, Faculty of Science, Cairo University, Giza, 12613, Egypt

*Email: mnadia@sci.cu.edu.eg

**Biological Methods:**

MTT assay

Cell lines were used to measure the cell growth effects of the compounds using the MTT assay. This colorimetric assay is depend on the conversion of the yellow tetrazolium bromide (MTT) to a purple formazan derivative by mitochondrial succinate dehydrogenase in living cells. Cell lines were cultured in RPMI-1640 medium supplemented with 10% fetal bovine serum. The antibiotics added were 100 units/ml penicillin and 100 µg/ml streptomycin at 37 ^o^C in a 5% CO_2_ incubator. Cell lines were seeded in a 96-well plates at a density of 1.0x10^4^ cells/well and cultured at 37 ^o^C for 48 h under 5% CO_2_. After incubation, the cells were treated with different concentrations of compounds and incubated for 24 hr. After 24 hr of drug treatment, 20 µl of MTT solution at 5 mg/ml was added to each well and incubated for 4 hr. 100 µl of dimethyl sulfoxide (*DMSO*) was added into each well to dissolve the purple formazan formed. The colorimetric assay was measured and recorded at absorbance of 570 nm using a plate reader (EXL 800, USA). Relative cell viability (%) was calculated as (A570 of treated sample/A570 of untreated sample) x 100 [36, 37].

**Tropomyosin receptor kinase A (TrKA) inhibitory assay**

The TrkA assay Kit is designed to measure TrkA activity for screening and profiling applications using Kinase-Glo® MAX as a detection reagent. The TrkA Assay Kit comes in a convenient 96-well format, with enough purified recombinant TrkA enzyme, TrkA substrate, ATP and kinase assay buffer for 100 enzyme reactions.

The assay protocol: all samples and controls should be tested in duplicate. Dissolve 5x Kinase Assay Buffer 1, ATP, and PTK Substrate Poly (Glu:Tyr 4:1) (10 mg/ml). 2). The master mixture (25 μl per well) was prepared by N wells x (6 μl 5x Kinase Assay Buffer 1 + 1 μl ATP (500 μM) + 1 μl PTK Substrate Poly (Glu:Tyr 4:1) (10mg/ml) + 17 μl water). Add 5 μl of Inhibitor solution of each well labeled as “Test Inhibitor”. For the “Positive Control" and “Blank”, add 5 μl of the same solution without inhibitor (Inhibitor buffer). Prepare 3 ml of 1x Kinase assay buffer 1 by mixing 600 μl of 5x Kinase assay buffer 1 with 2400 μl water 3 ml of 1x Kinase assay buffer is sufficient for 100 reactions. To the wells designated as "Blank", add 20 μl of 1x Kinase assay buffer 1and thaw TrkA enzyme on ice. Upon first thaw, briefly spin tube containing enzyme to recover full content of the tube. Calculate the amount of TrkA required for the assay and dilute enzyme to 5 ng/μl with 1x Kinase assay buffer. Store remaining undiluted enzyme in aliquots at -80°C. Then initiate reaction by adding 20 μl of diluted TrkA enzyme to the wells designated “Positive Control” and "Test Inhibitor Control". Incubate at 30°C for 45minutes and thaw Kinase-Glo Max reagent. After the 45 minute reaction, add 50 μl of Kinase-Glo Max reagent to each well. Cover plate with aluminum foil and incubate the plate at room temperature for 15 minutes. Immediately read sample in a luminometer or microtiter-plate capable of reading chemiluminescence. “Blank” value is subtracted from all readings.

**In-vitro cell cycle analysis**

HepG-2 cells were pre-cultured in 25 cm^2^ cell culture flasks; RPMI-1640 medium was used. Tested compounds **7b**, **15b, 16a** and **16c** were dissolved separately in the required medium and used in the cell treatment at their IC_50_. After overnight treatment the cells were collected and fixed gently with 70% (v/v) ethanol in PBS, incubated overnight at 4 ^o^C, and resuspended in PBS containing 40 µg/ml propidium iodide (PI), 0.1 mg/ml RNase and 0.1% (v/v) Triton X-100 in the dark room at after 30 min at 37 ^o^C, the cell cycle analysis was performed using a flow cytometer, equipped with an argon ion laser at a wavelength of 488 nm (Becton-Dickinson, San Josa, CA, USA) and the percentage of cells at G1, S and G2/M was also calculated.

**Annexin V-FITC apoptosis assay**

As mentioned above, HepG-2 cells were collected and incubated separately with compounds **7b**, **15b, 16a** and **16c** for 48 hr. Cells were then collected and washed twice consecutively with PBS and centrifuged. After that, the cells were treated with Annexin V-FITC and propidium iodide (PI) using an apoptosis detection kit (BD Biosciences, San Jose, CA) according to the manufacturer's instructions. The biding of Annexin V-FITC and PI was analyzed by ﬂow cytometer.

**Molecular docking study**

Molecular docking studies were performed using program “Molecular Operating Environment (MOE) 2009.10. Protein structure was downloaded from the PDB data bank (http://www.rcsb.org/PDB codes: 5H3Q). The protein was prepared by removing the ligand molecule and water from the enzyme active site. Then, add hydrogen atoms to the proteins and minimized keeping all the heavy atoms fixed until reached the values of RMS gradient to 0.01 kcal mol^-1^, and RMS distance to 0.1 Ǻ using MOE. Building the ligands by the MOE builder interface and subjecting the structures to energy minimization using MMFF94x force field and computing the partial charges using the same force field. Finally, docking calculations were carried out using the Alpha triangle placement method and pose prioritization was by the London dG scoring method.

**Spectral analysis:**


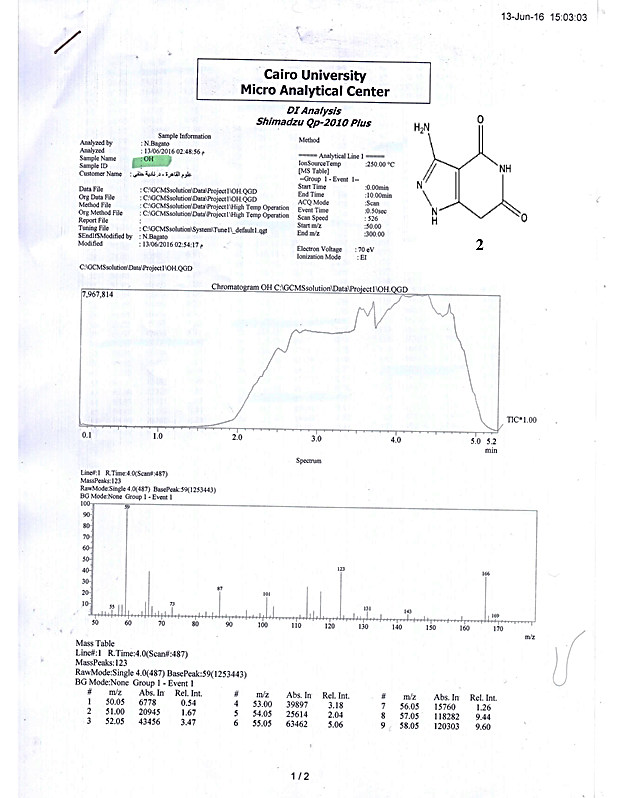


Figure (S1): Mass spectrum of compound **2**


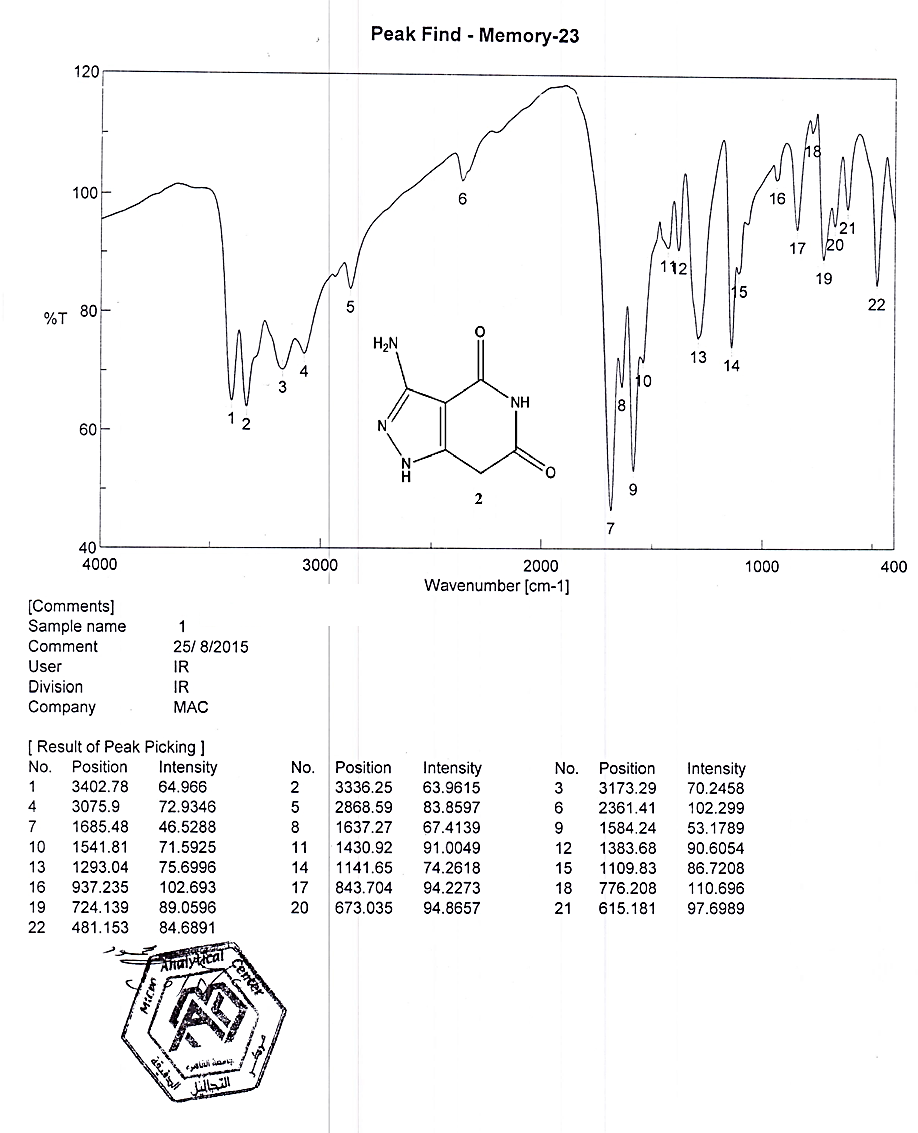


Figure (S2): IR spectrum of compound **2**


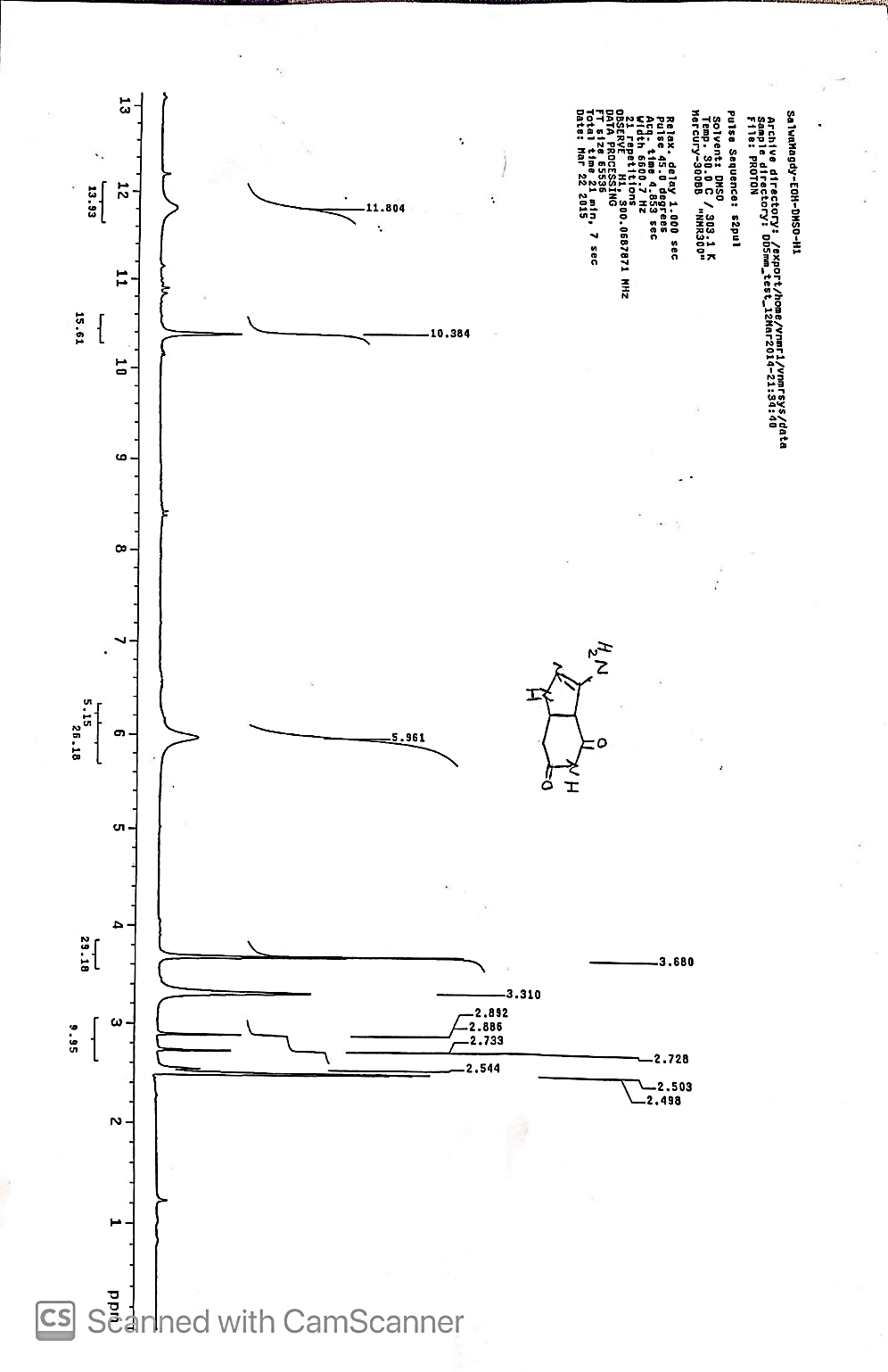


Figure (S3): ^1^H NMR spectrum of compound **2**


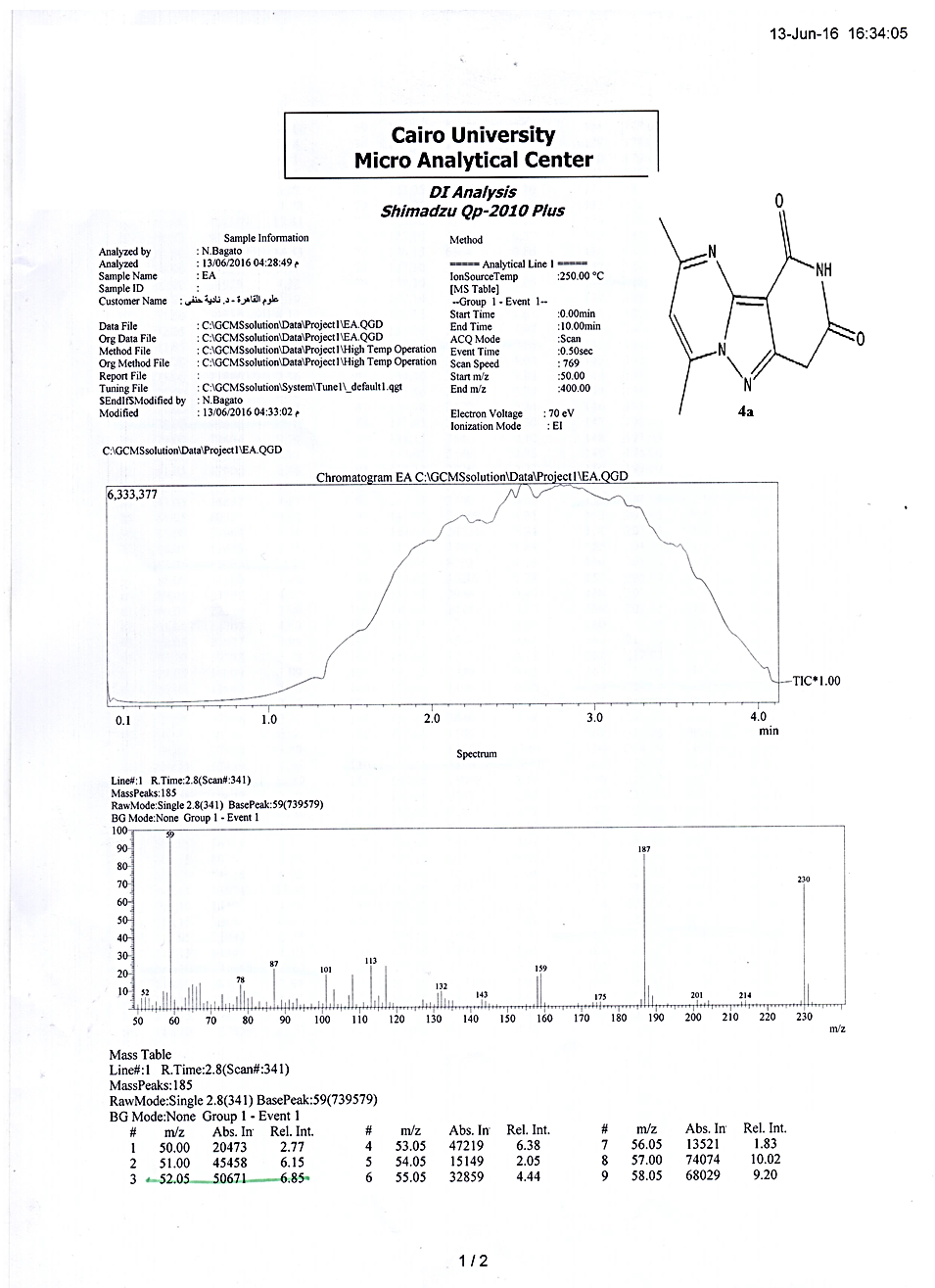


Figure (S4): Mass spectrum of compound **4a**


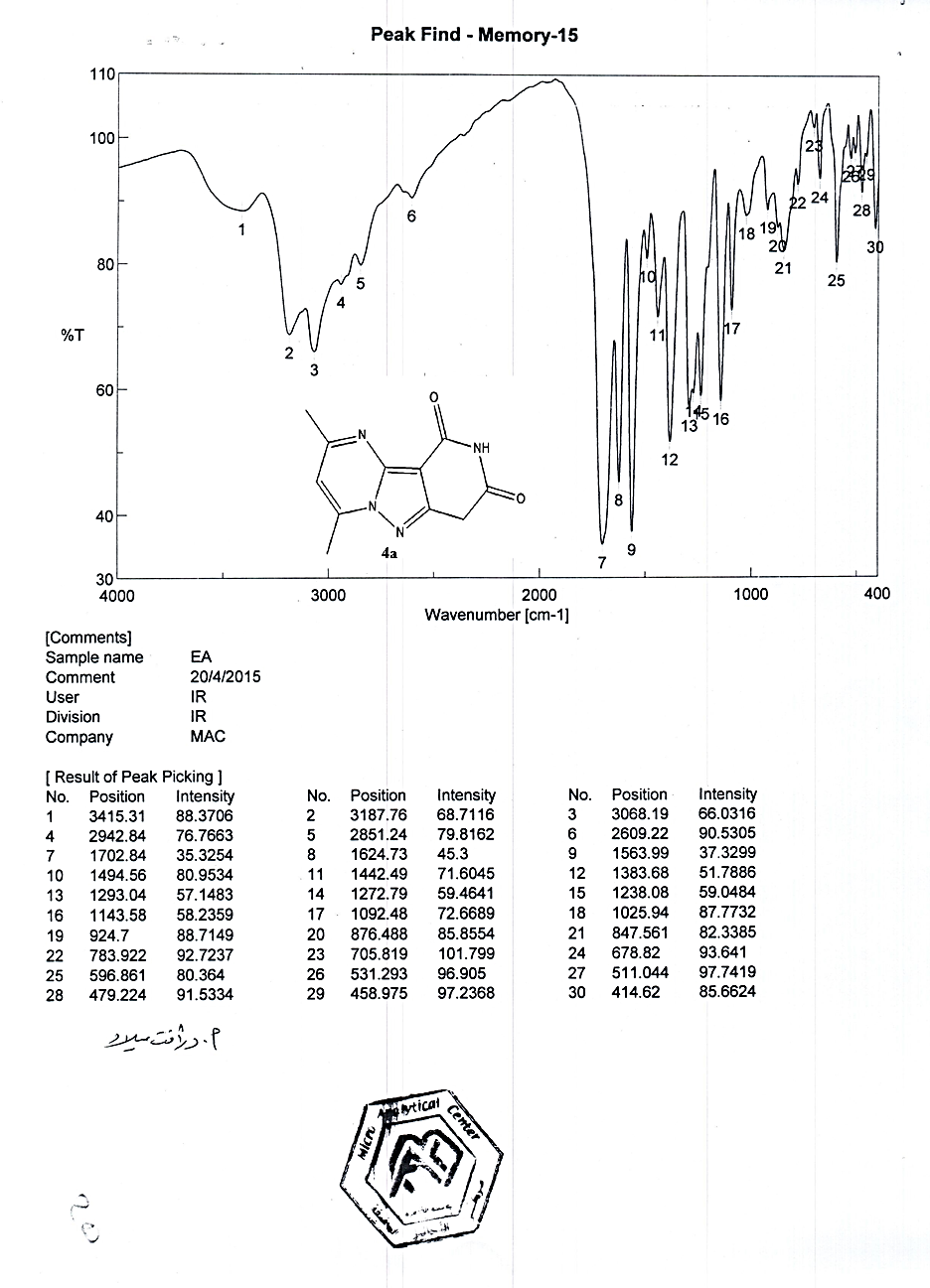


Figure (S5): IR spectrum of compound **4a**


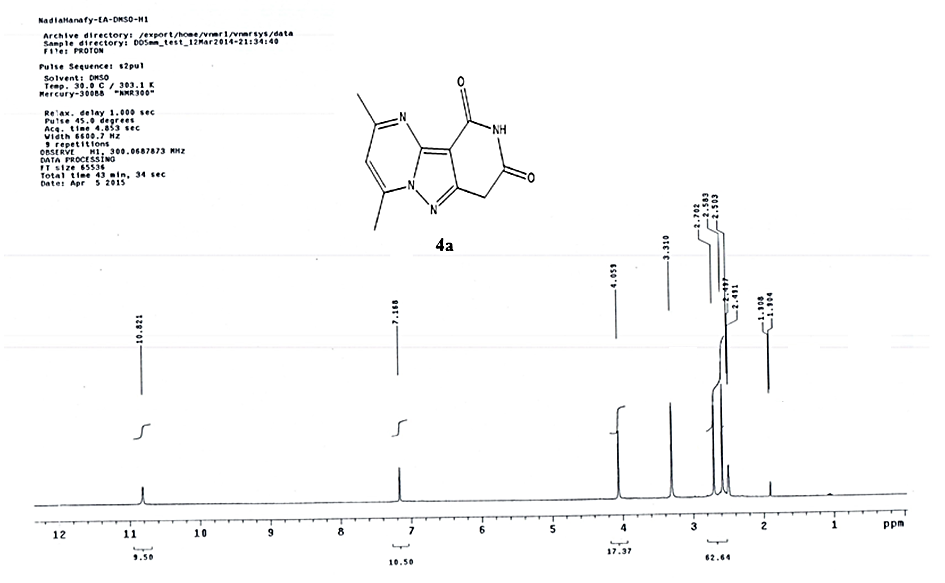


Figure (S6): ^1^H NMR spectrum of compound **4a**


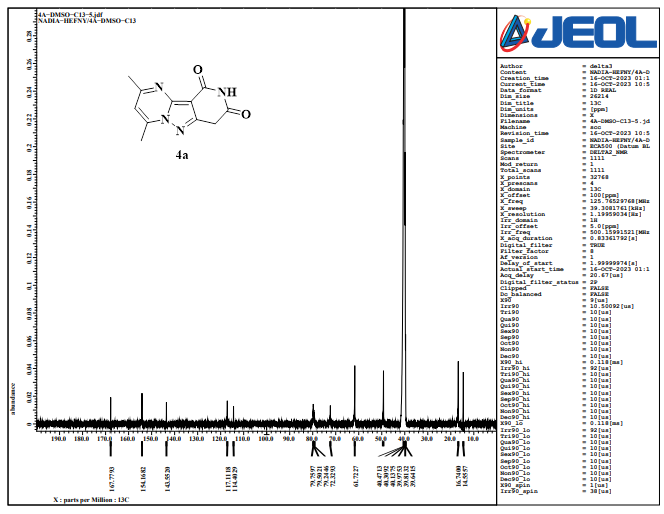


Figure (S7): ^13^C NMR spectrum of compound **4a**


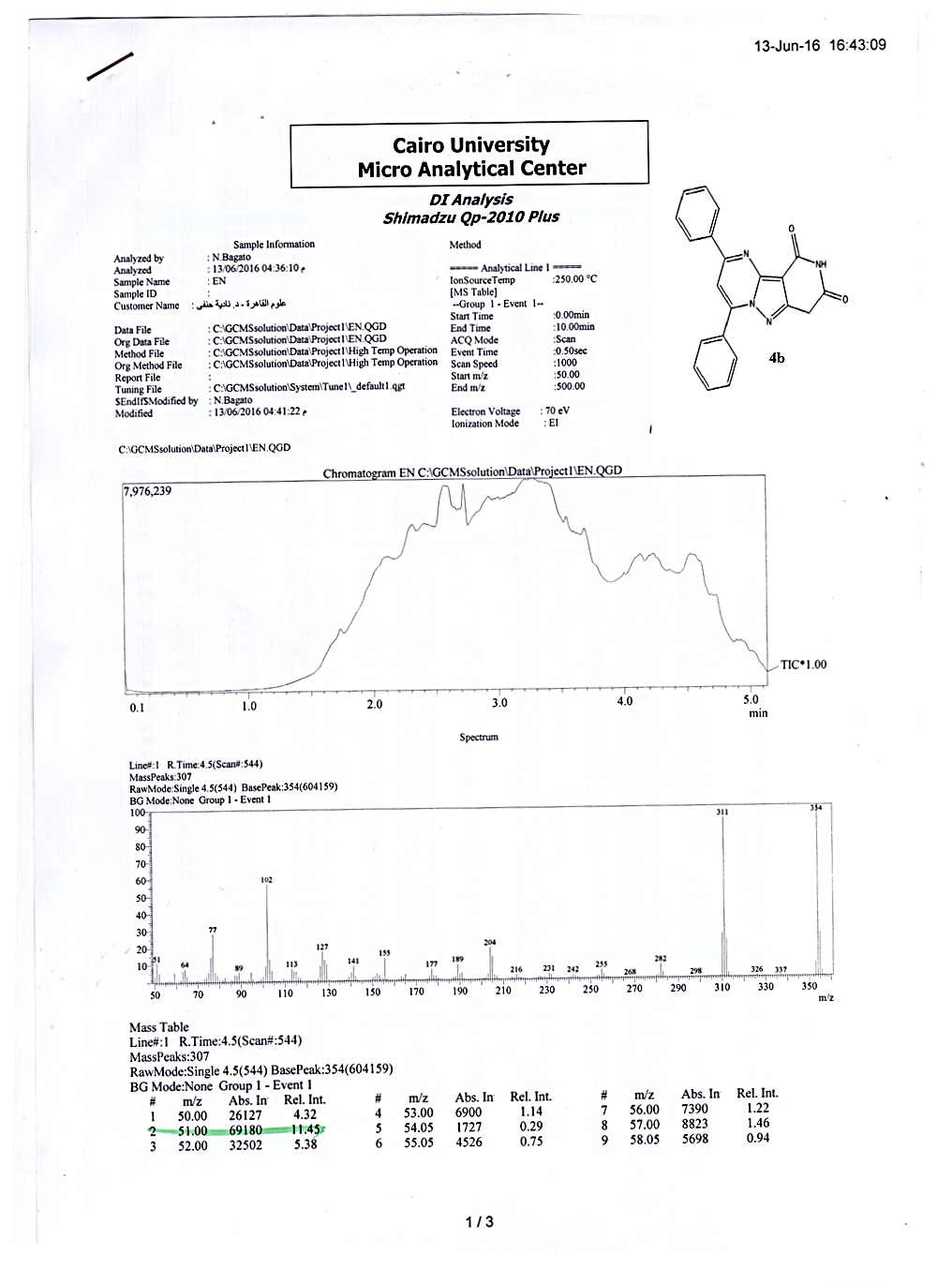


Figure (S8): Mass spectrum of compound **4b**


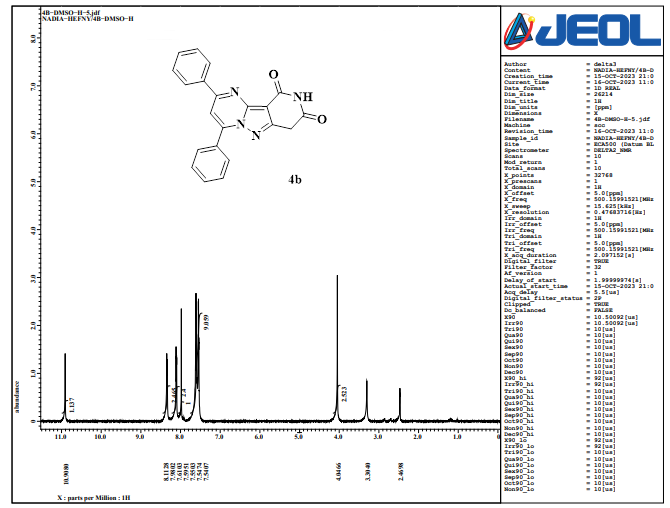


Figure (S9): ^1^H NMR spectrum of compound **4b**


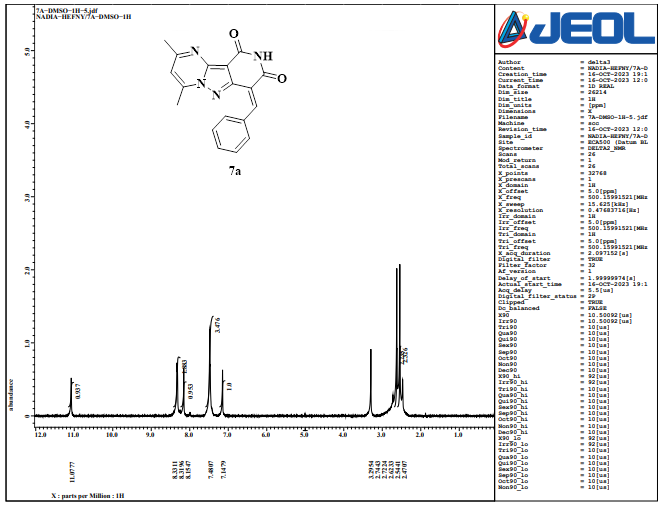


Figure (S10): ^1^H NMR spectrum of compound **7a**


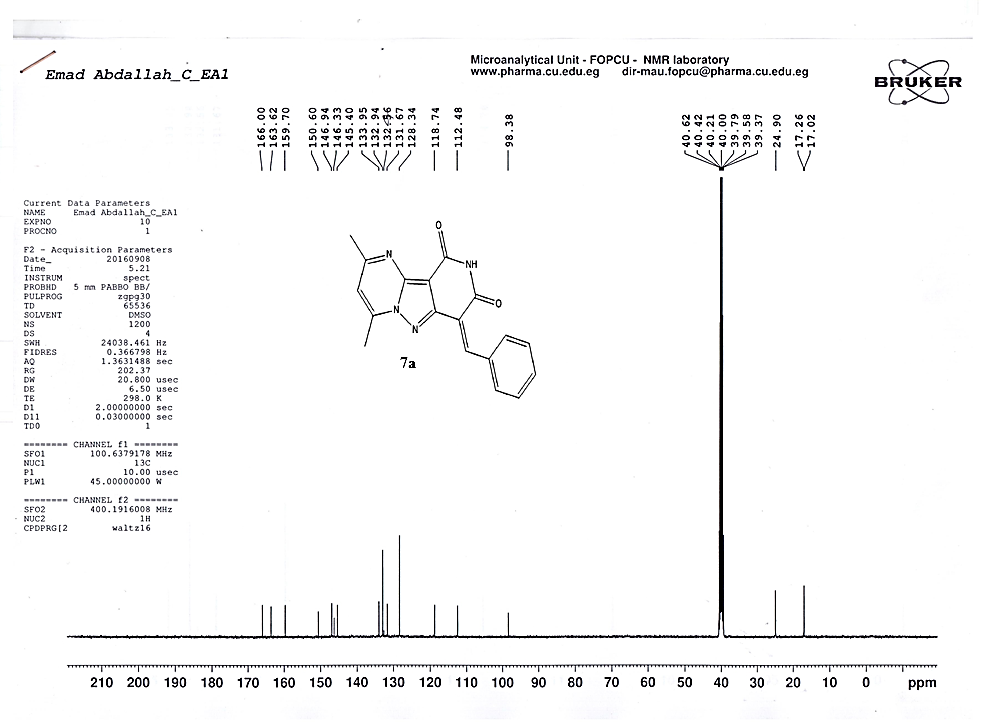


Figure (S11): ^13^C NMR spectrum of compound **7a**


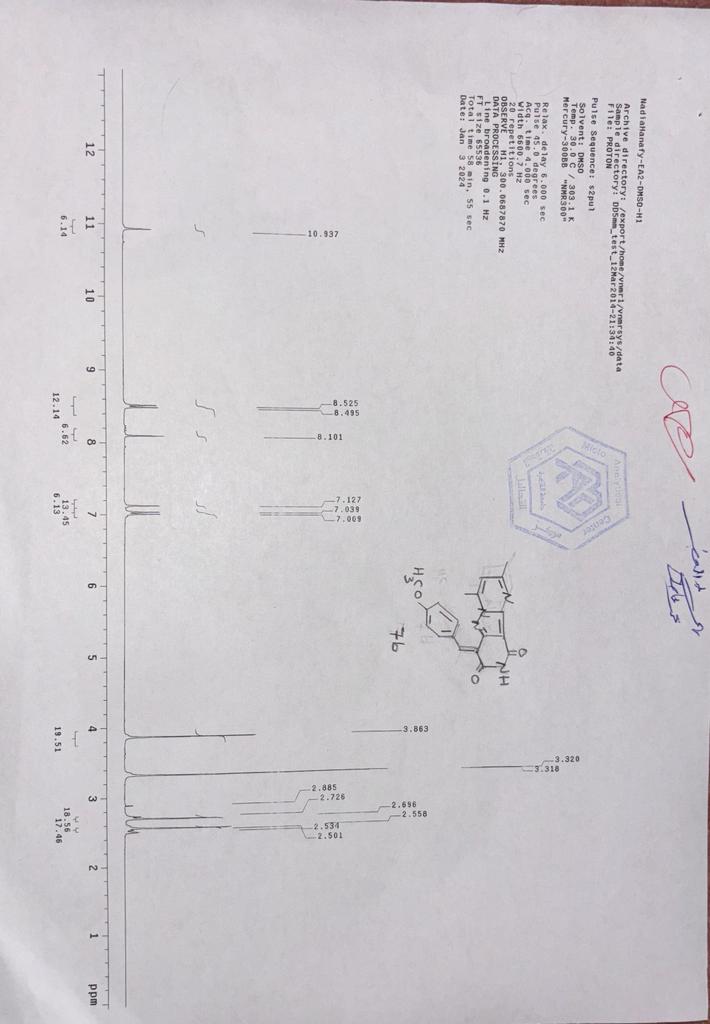


Figure (S12): ^1^H NMR spectrum of compound **7b**


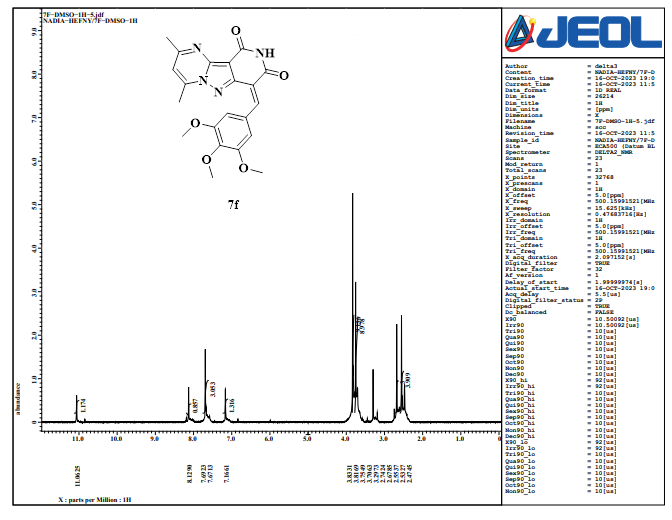


Figure (S13): ^1^H NMR spectrum of compound **7f**


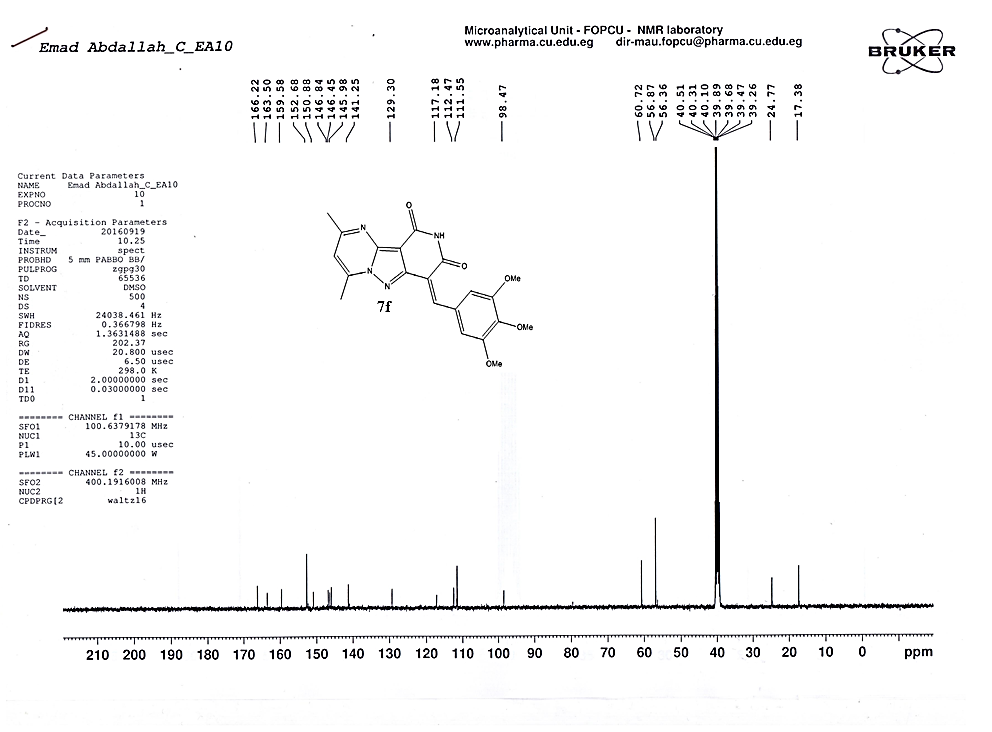


Figure (S14): ^13^C NMR spectrum of compound **7f**


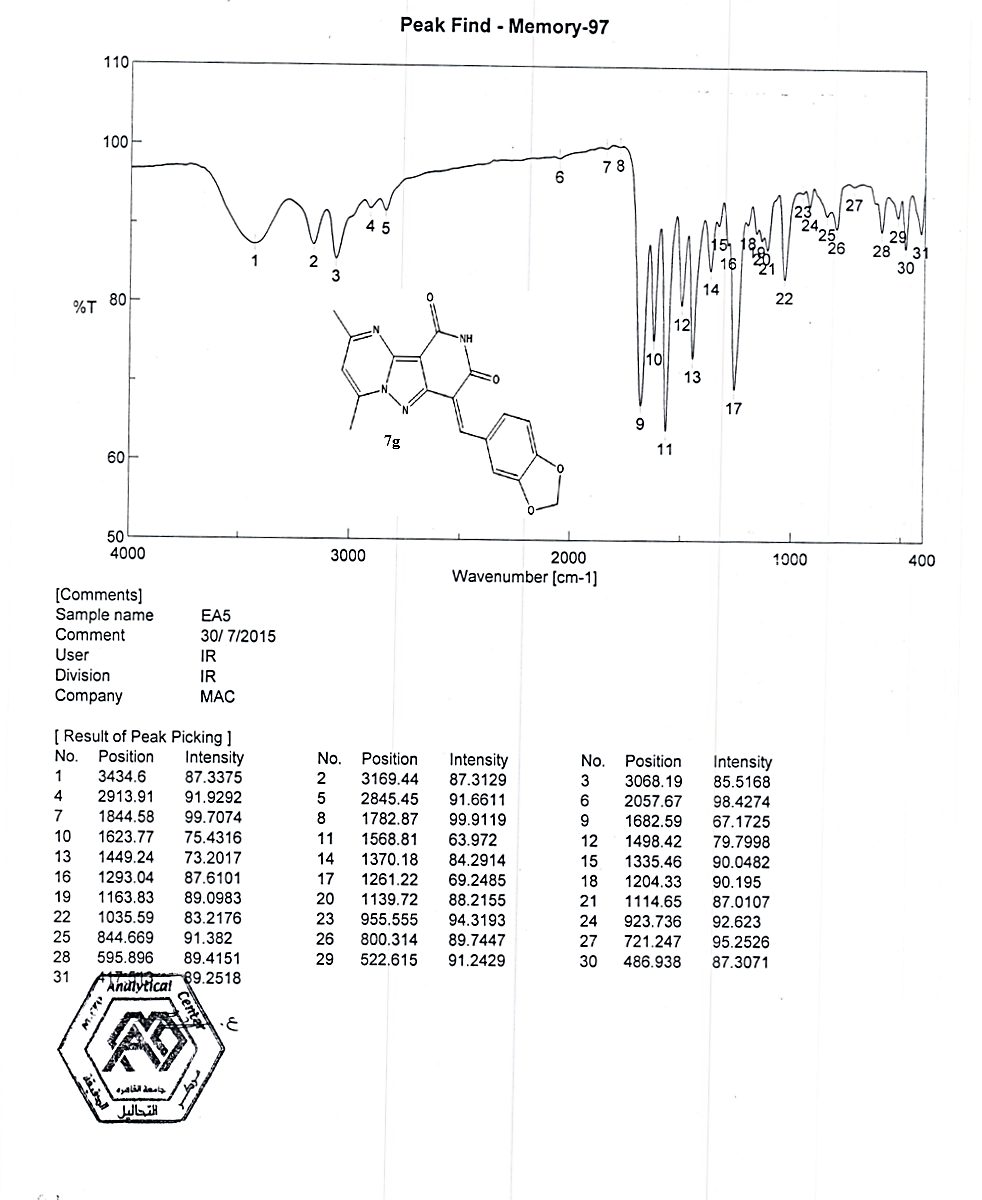


Figure (S15): IR spectrum of compound **7g**


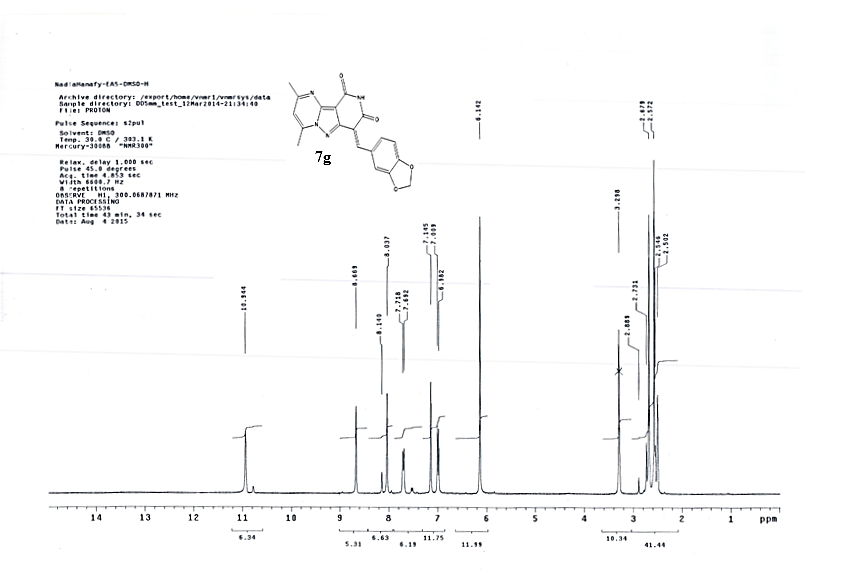


Figure (S16): ^1^H NMR spectrum of compound **7g**


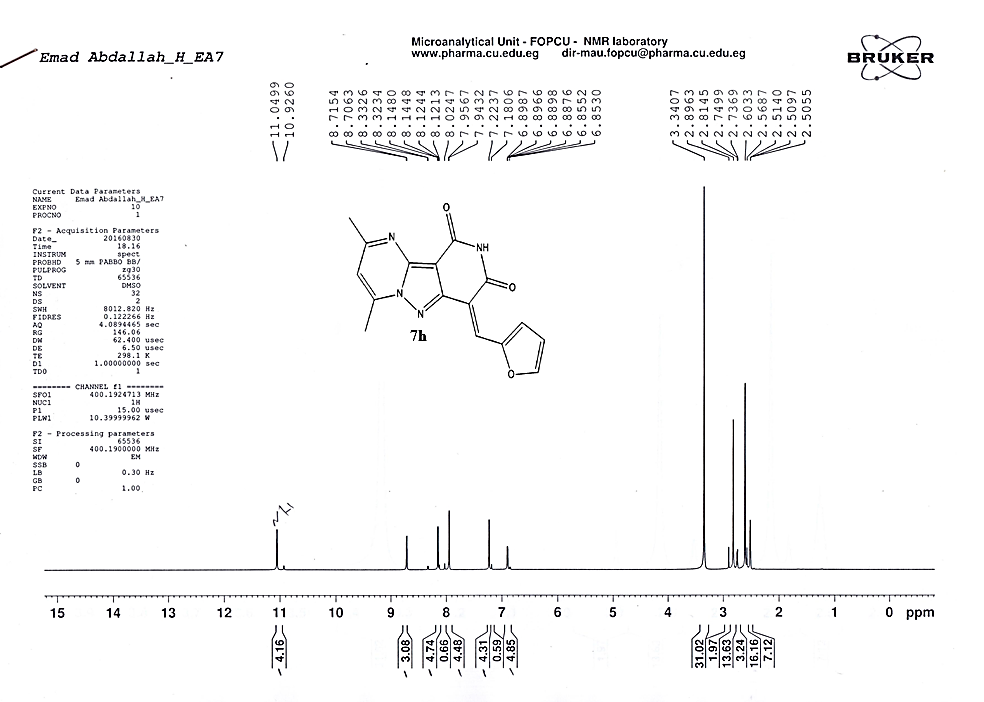


Figure (S17): ^1^H NMR spectrum of compound **7h**


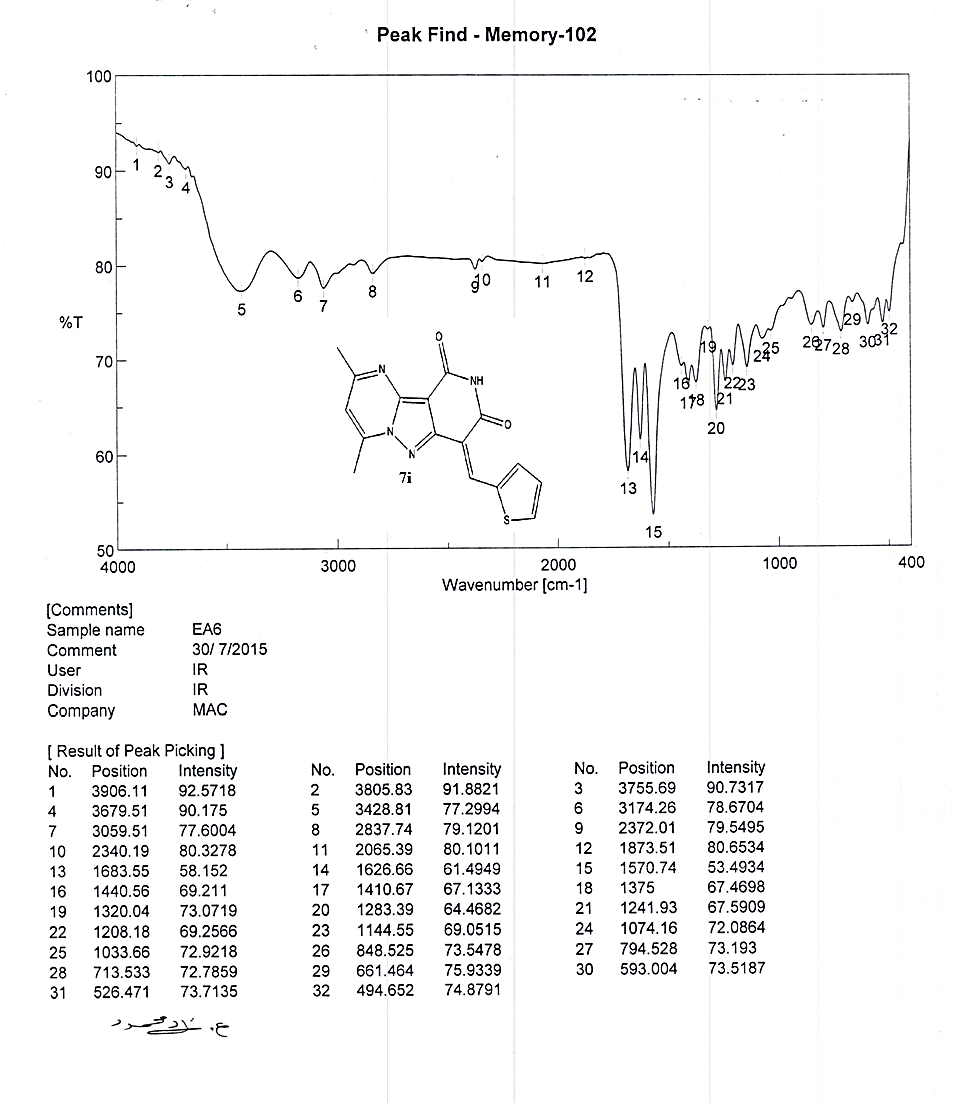


Figure (S18): IR spectrum of compound **7i**


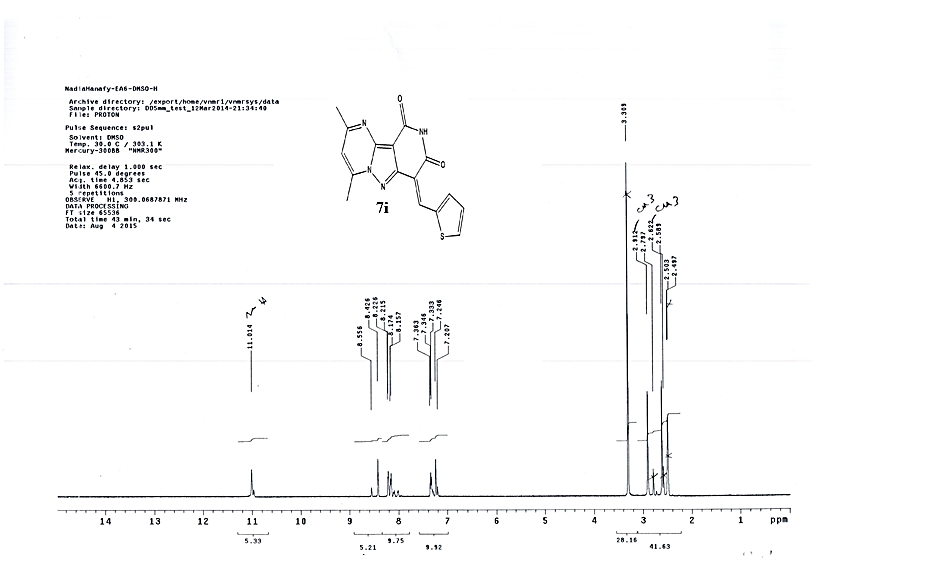


Figure (S19): ^1^H NMR spectrum of compound **7i**


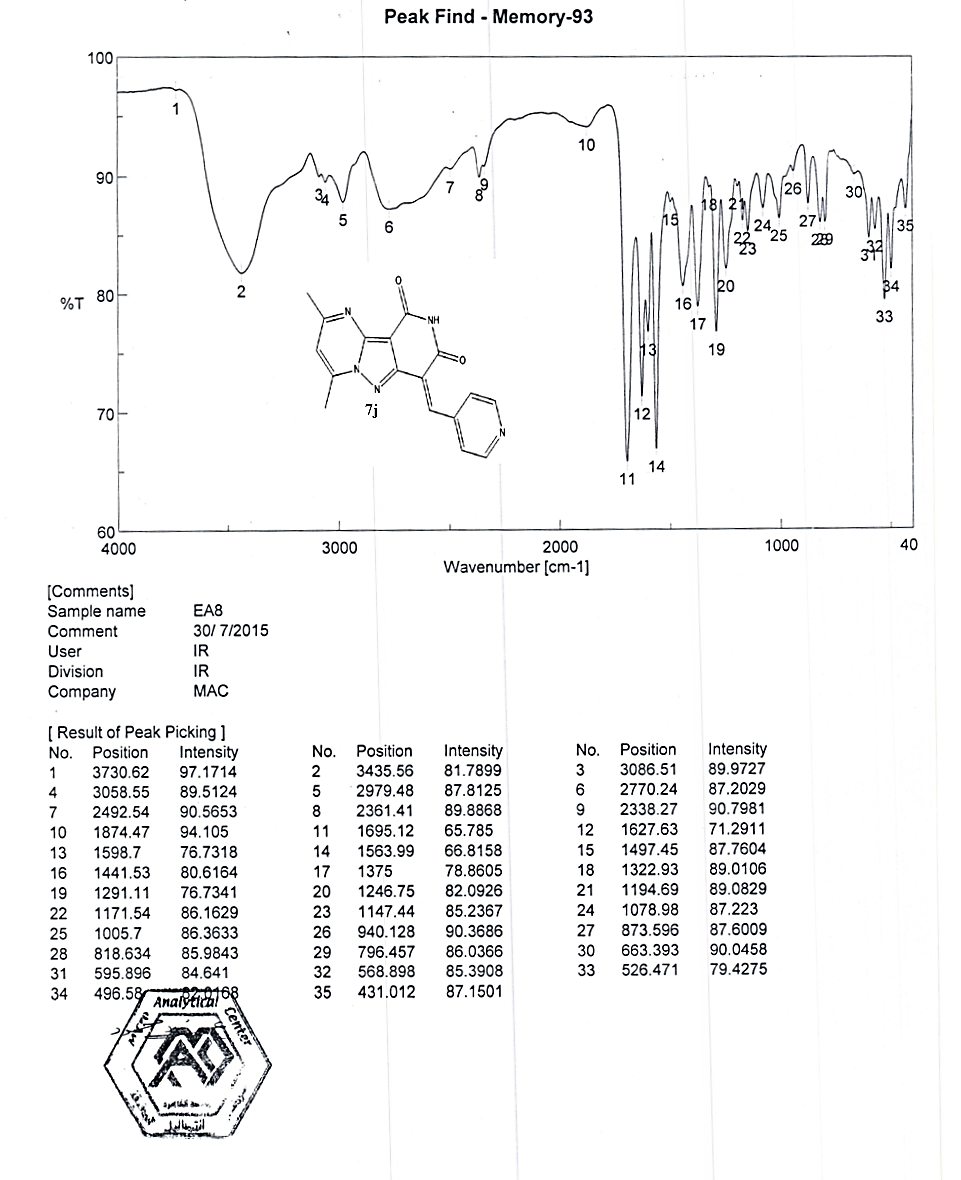


Figure (S20): IR spectrum of compound **7j**

**
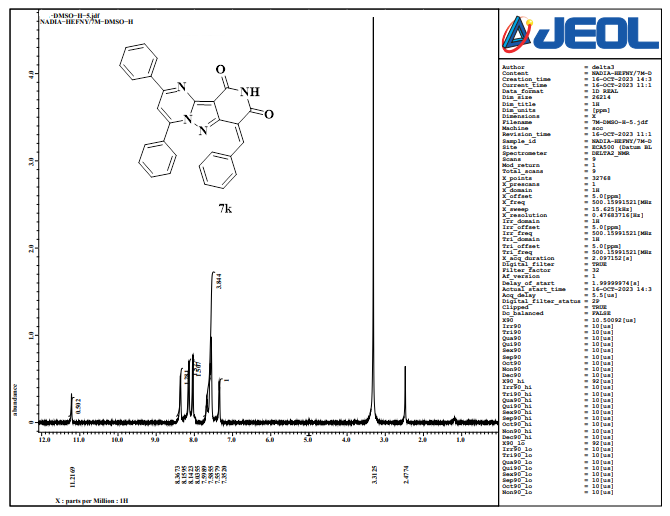
**

Figure (S21): ^1^H NMR spectrum of compound **7k**


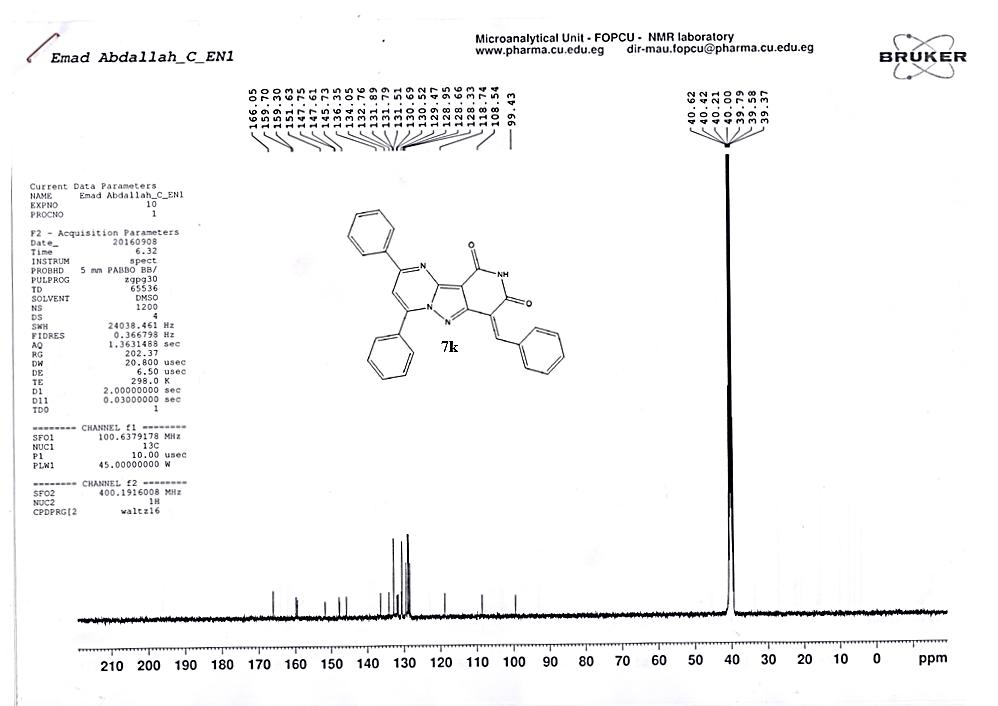


Figure (S22): ^13^C NMR spectrum of compound **7k**


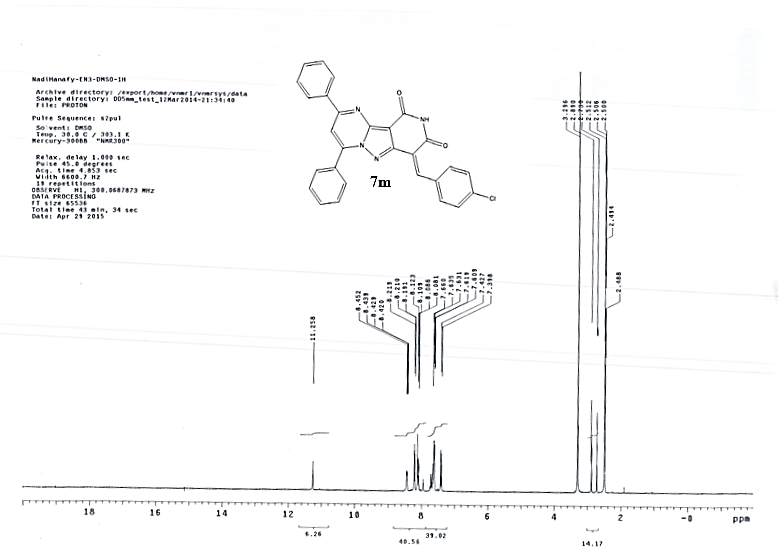


Figure (S23): ^1^H NMR spectrum of compound **7m**


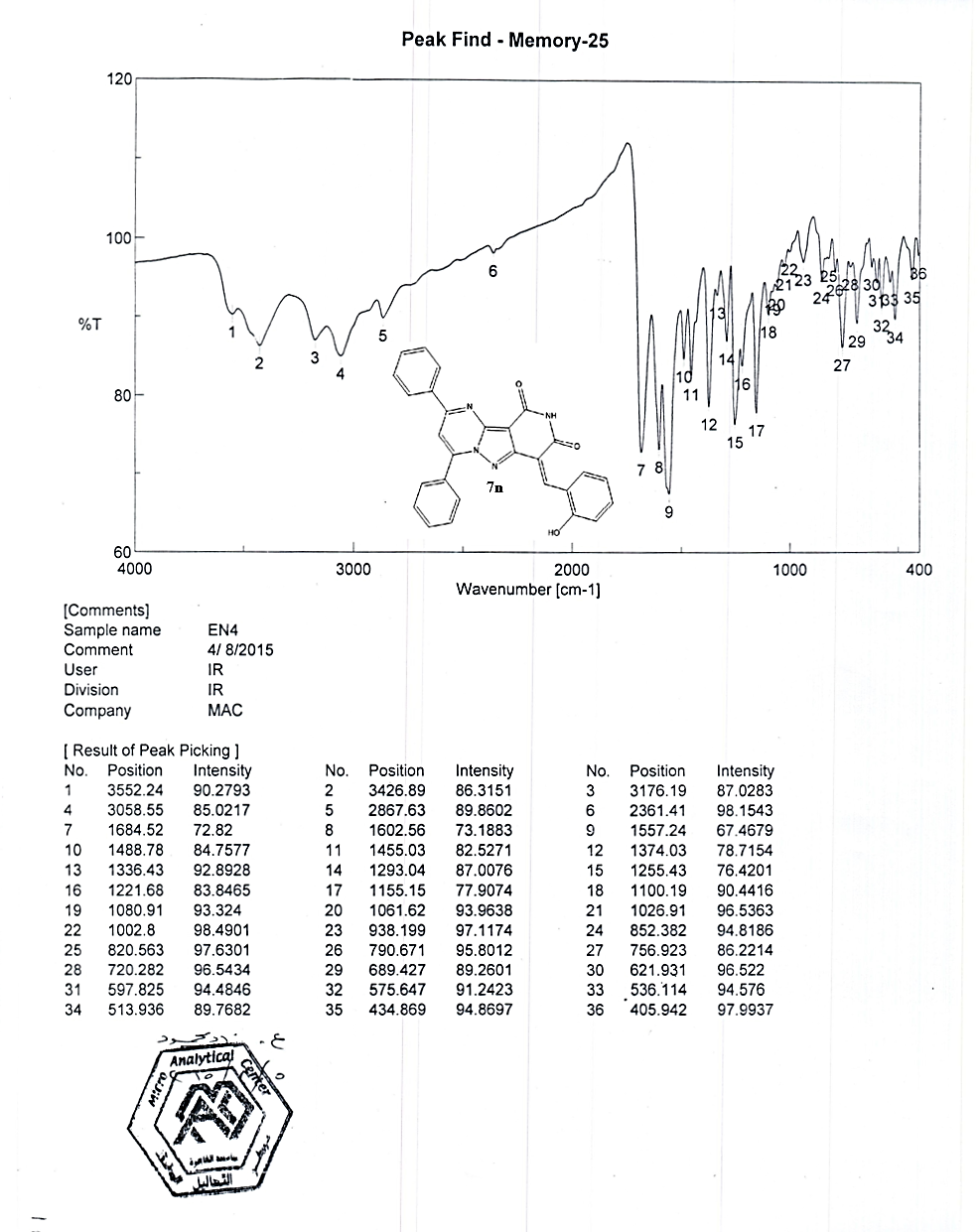


Figure (S24): IR spectrum of compound **7n**


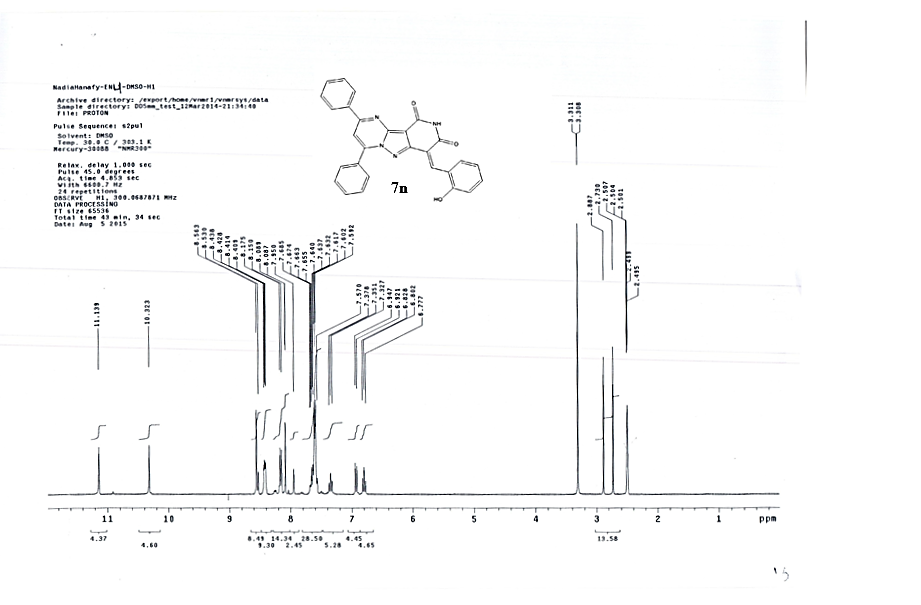


Figure (S25): ^1^H NMR spectrum of compound **7n**


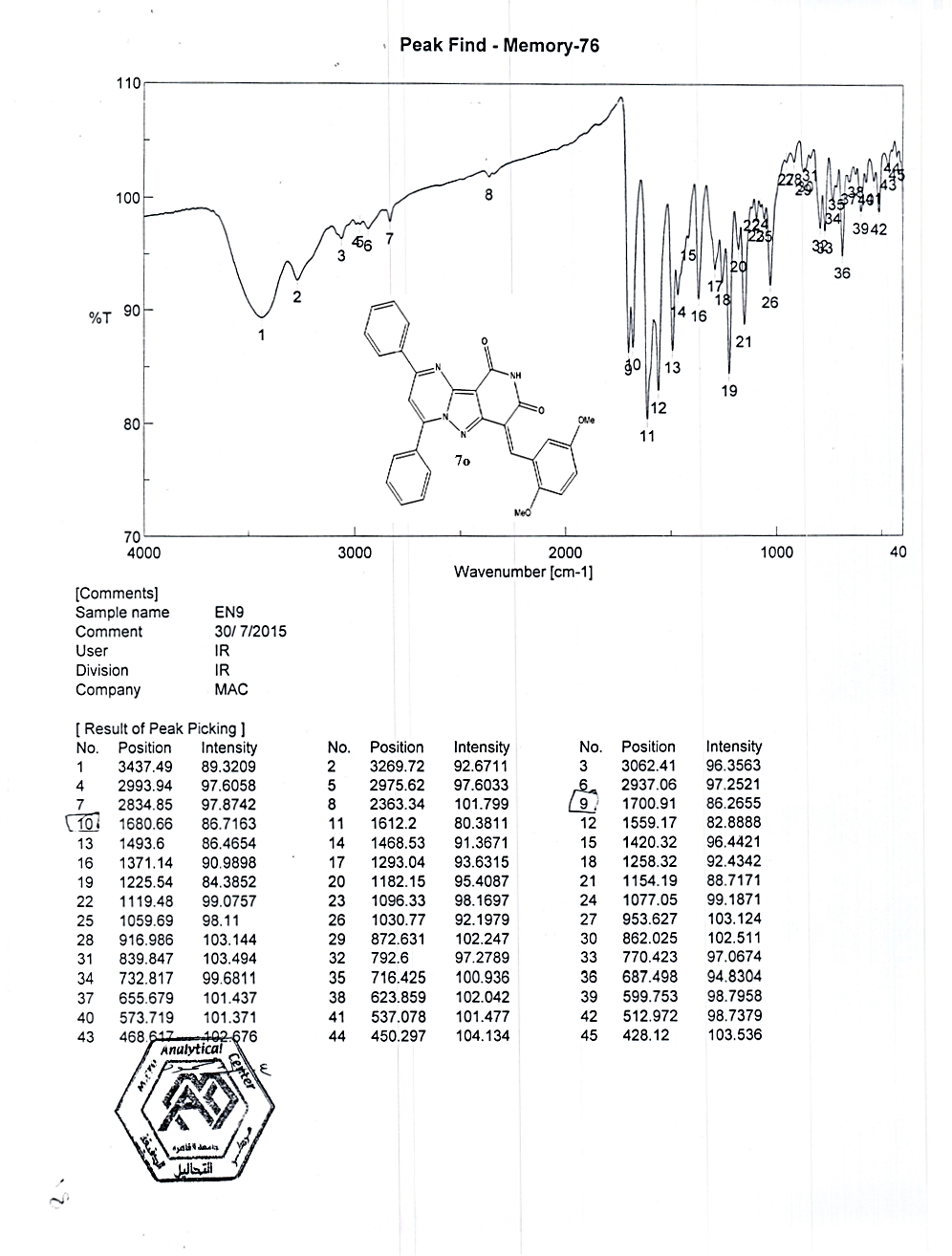


Figure (S26): IR spectrum of compound **7o**


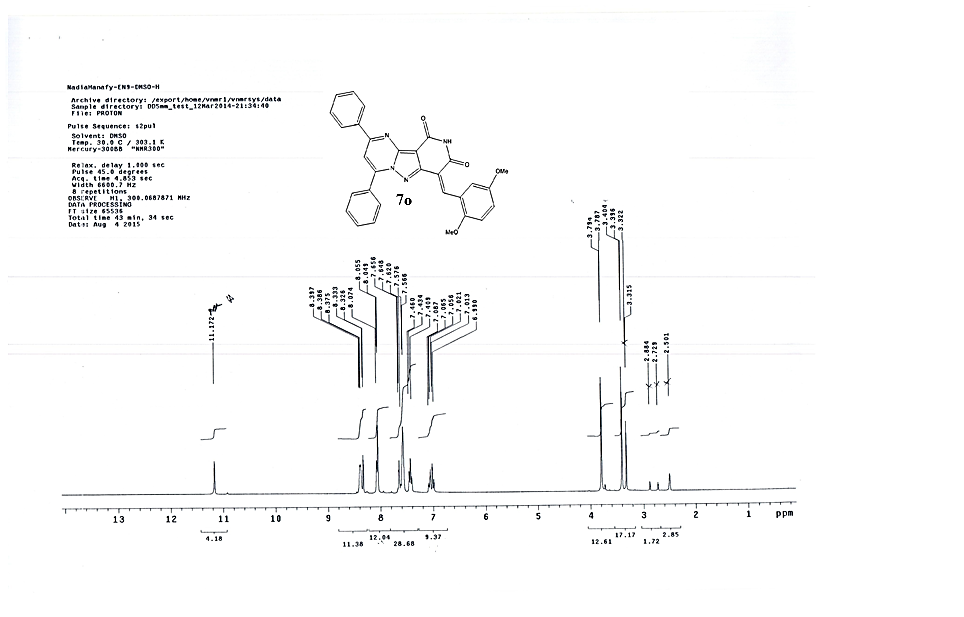


Figure (S27): ^1^H NMR spectrum of compound **7o**


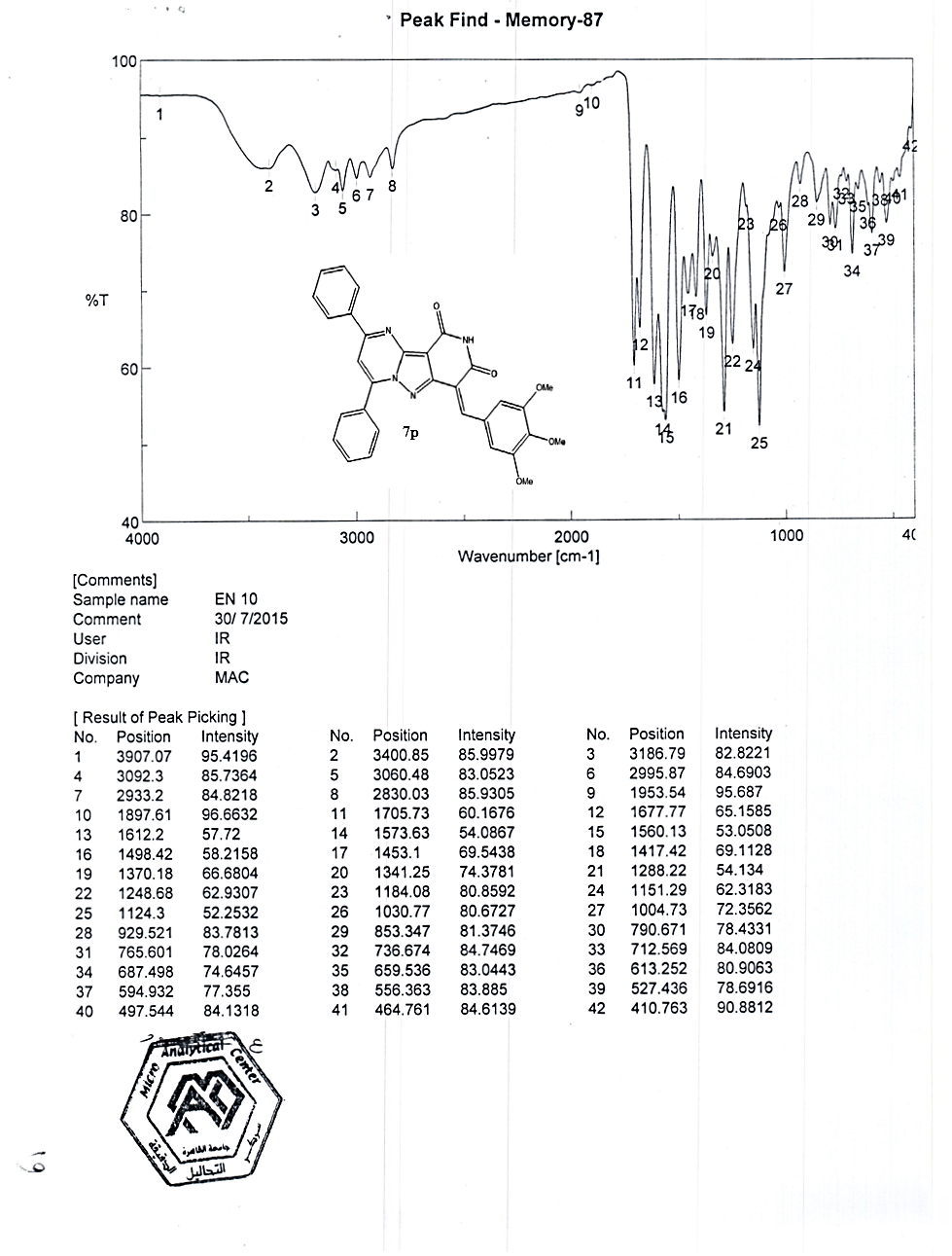


Figure (S28): IR spectrum of compound **7p**


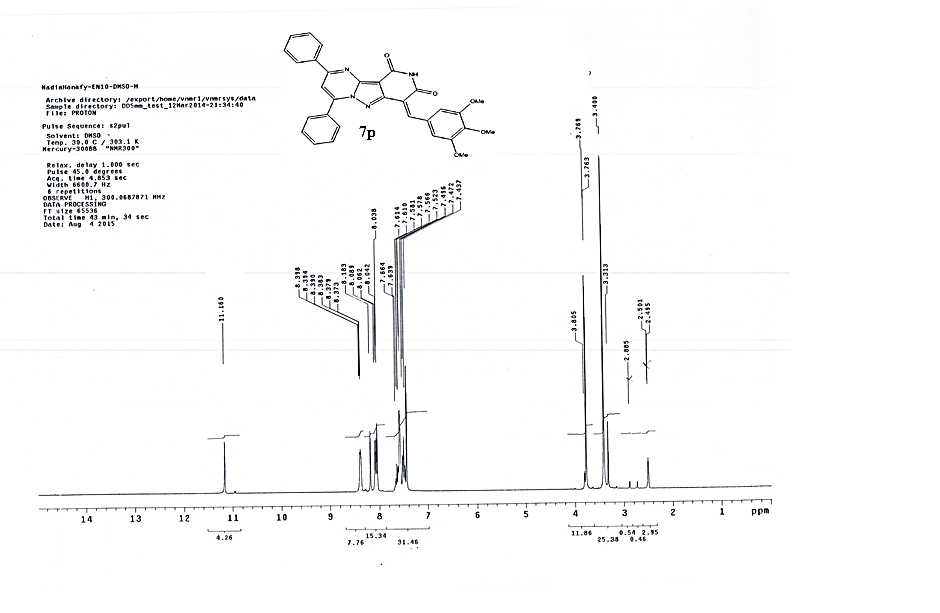


Figure (S29): ^1^H NMR spectrum of compound **7p**


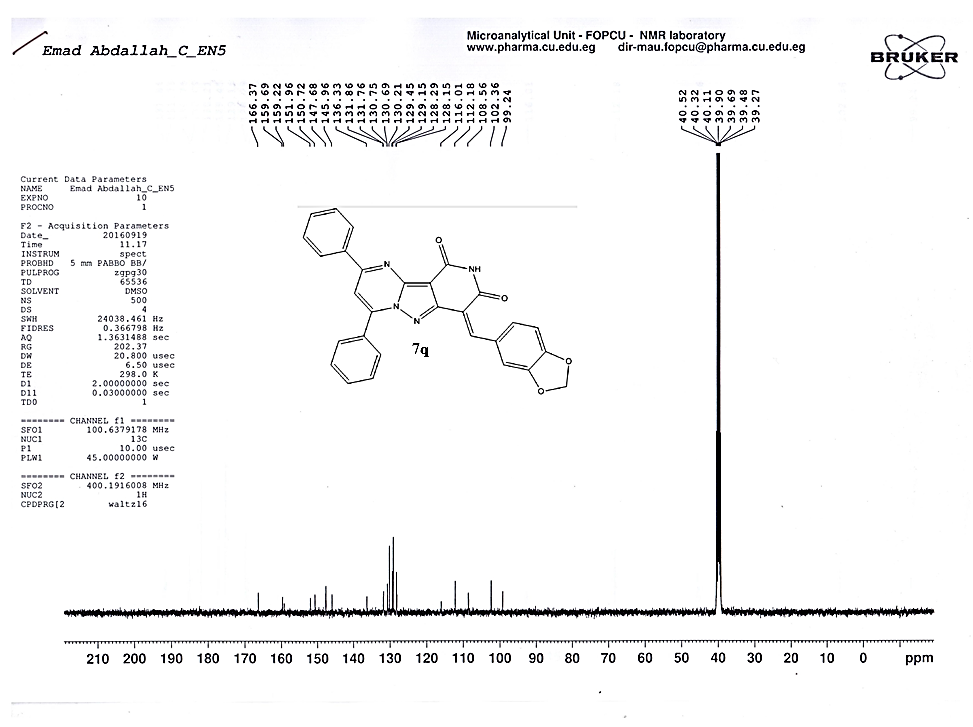


Figure (S30): ^13^C NMR spectrum of compound **7q**


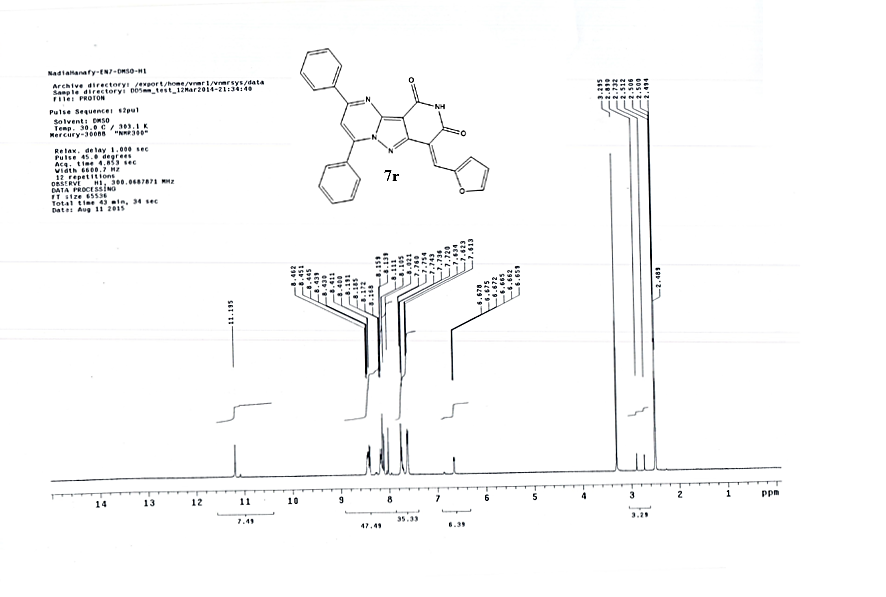


Figure (S31): ^1^H NMR spectrum of compound **7r**


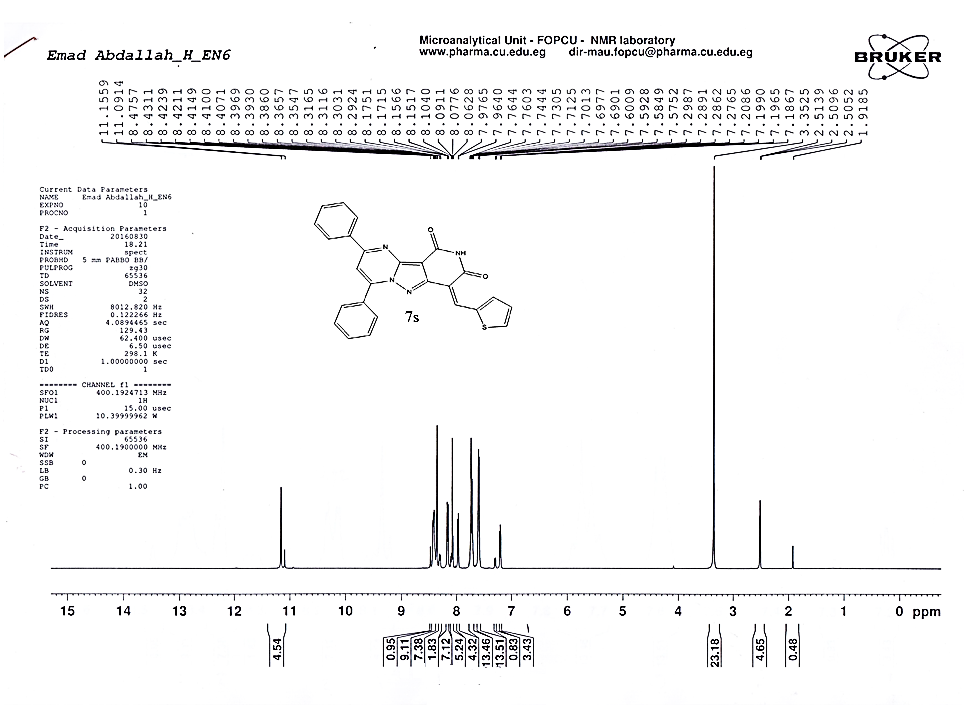


Figure (S32): ^1^H NMR spectrum of compound **7s**


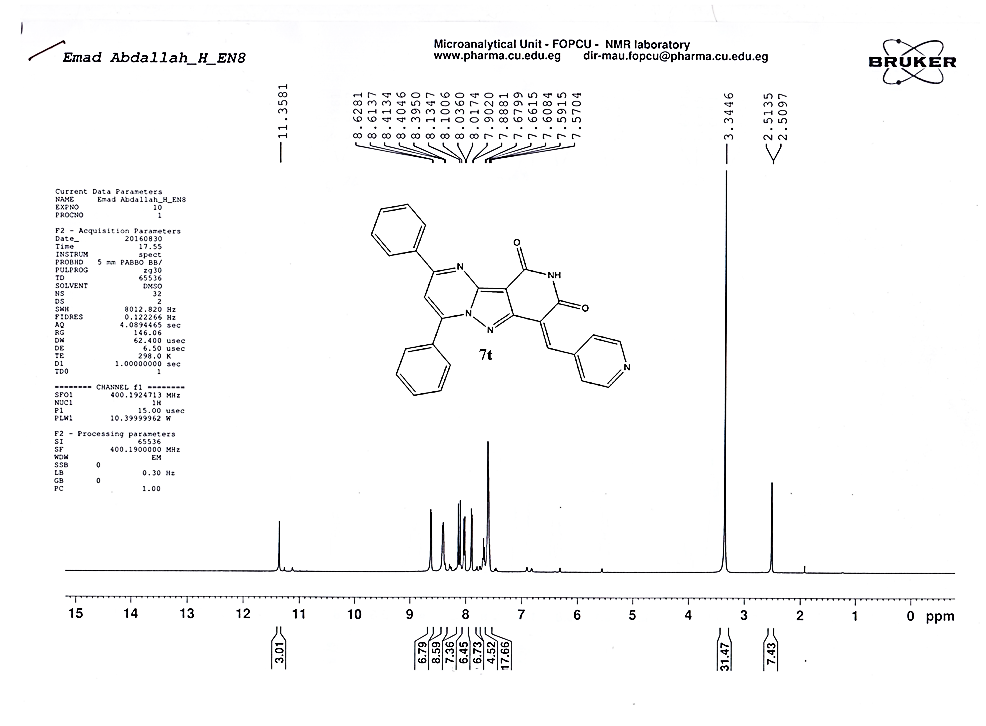


Figure (S33): ^1^H NMR spectrum of compound **7t**


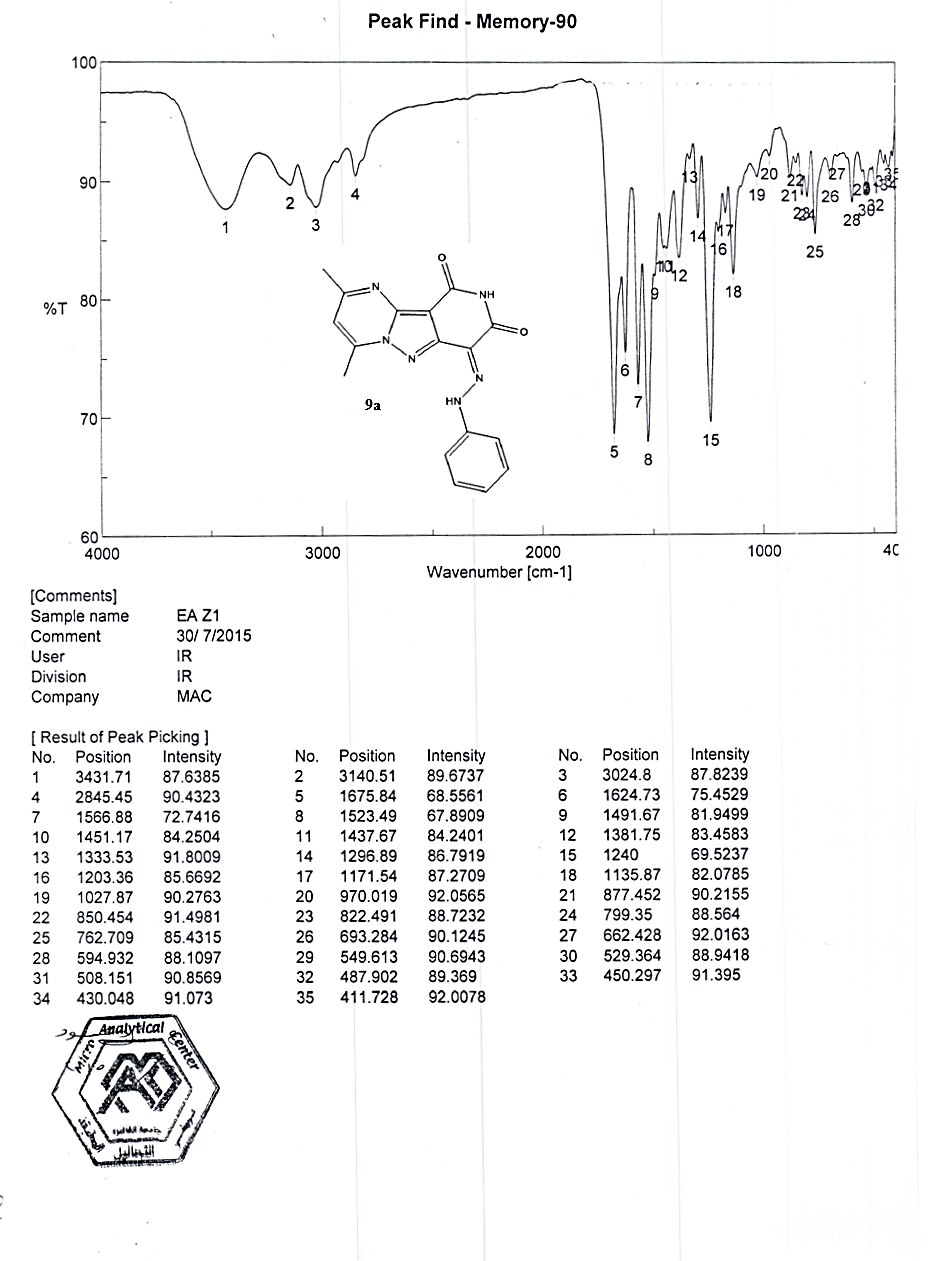


Figure (S34): IR spectrum of compound **9a**


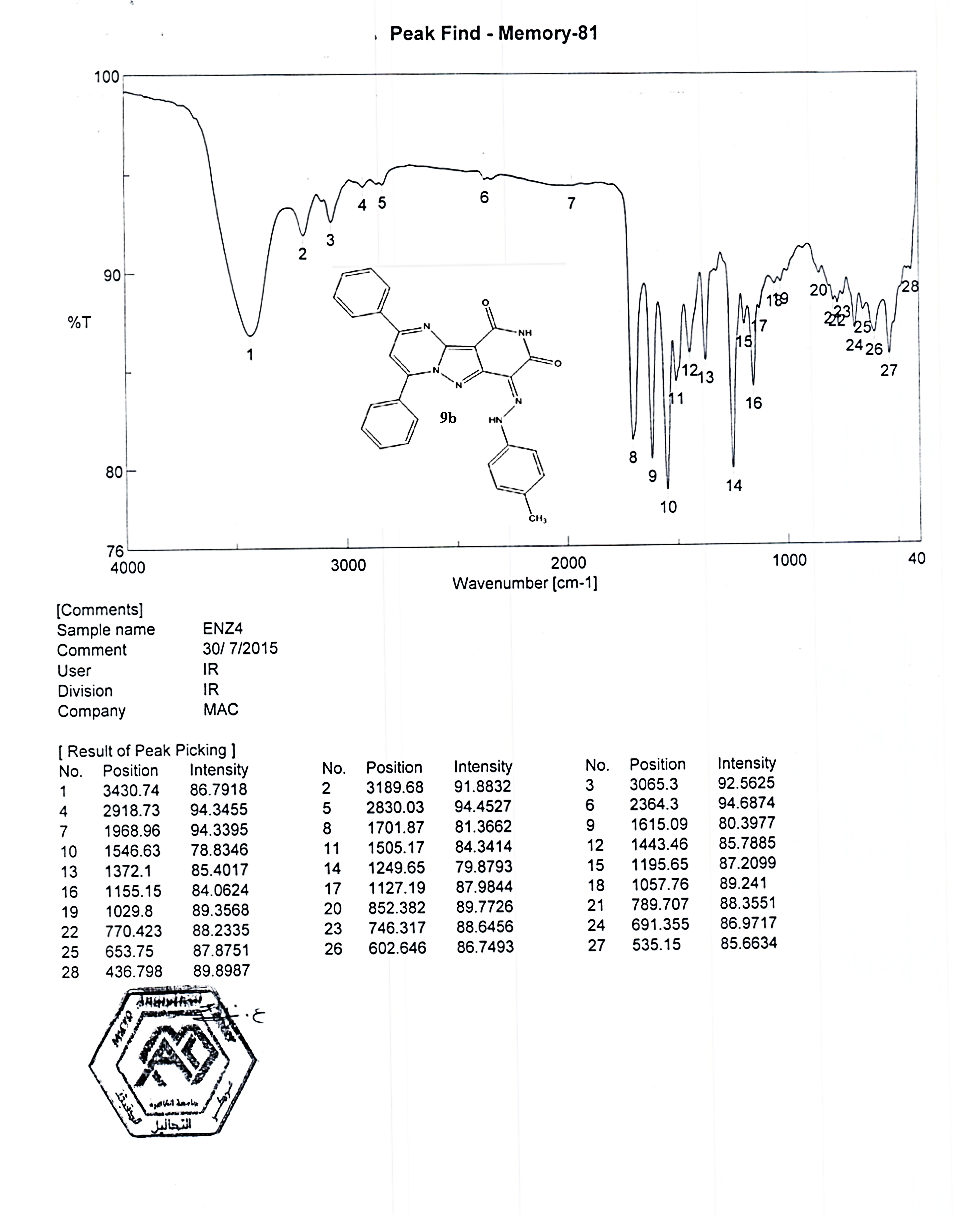


Figure (S35): IR spectrum of compound **9b**


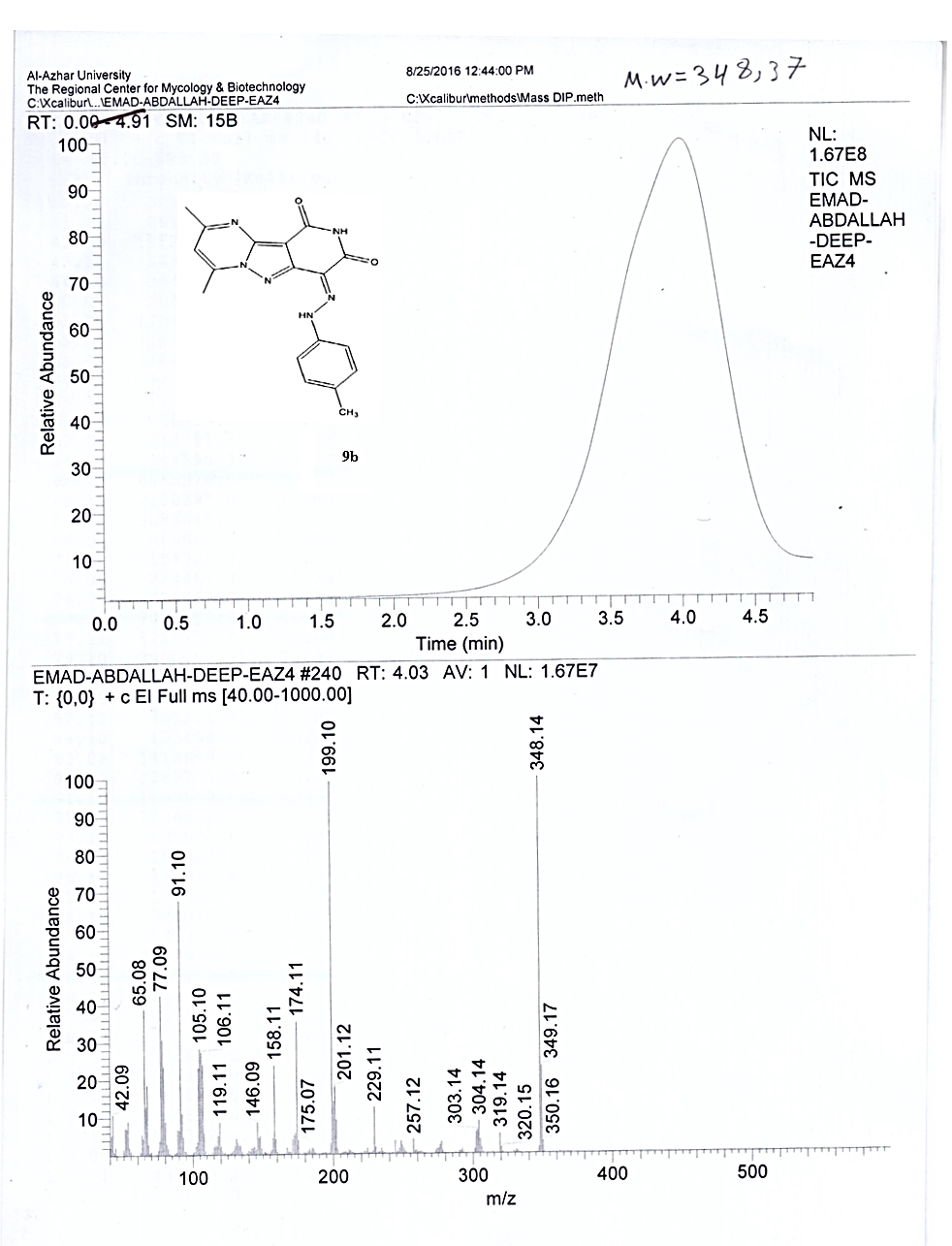


Figure (S36): Mass spectrum of compound **9b**


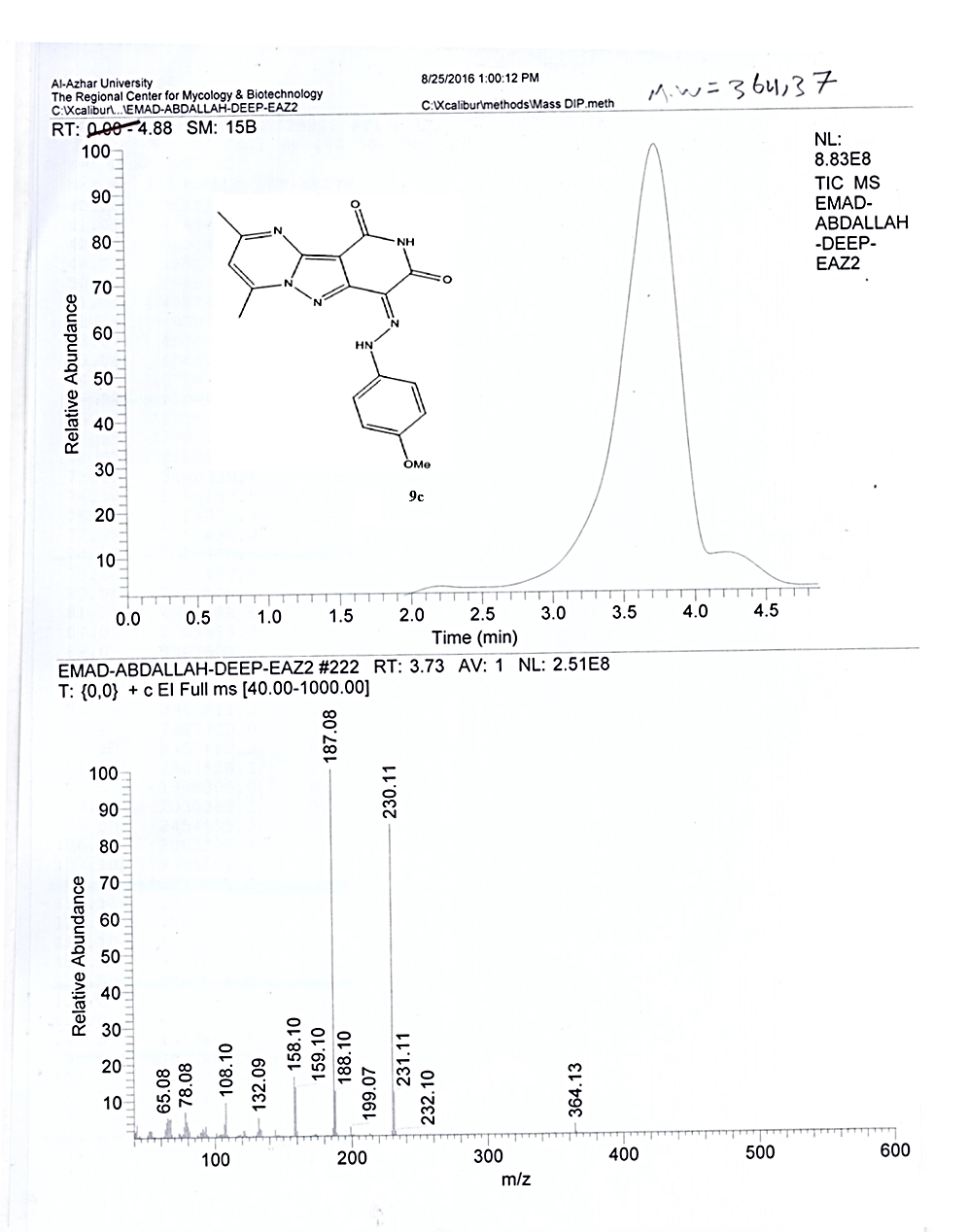


Figure (S37): Mass spectrum of compound **9c**


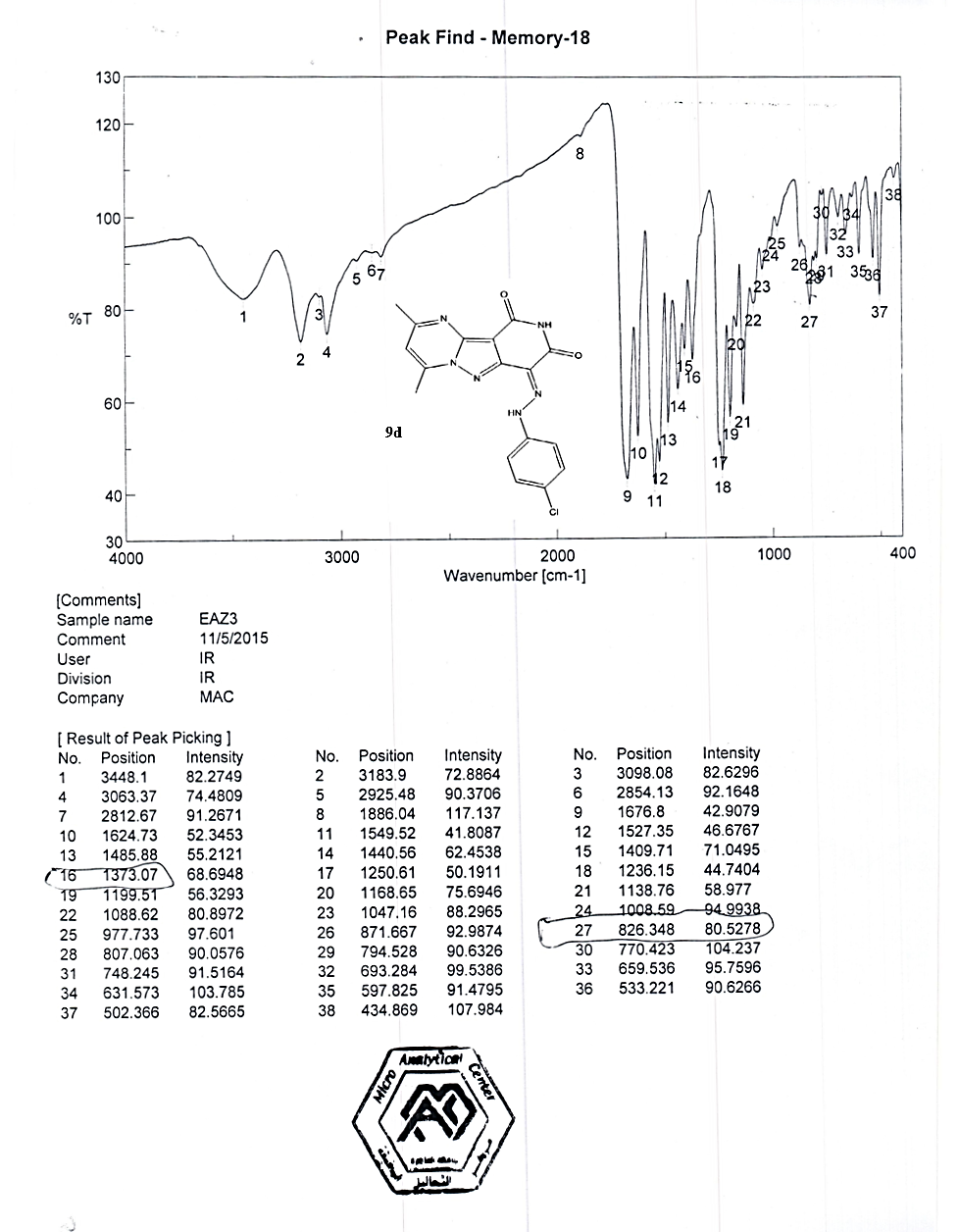


Figure (S38): IR spectrum of compound **9d**


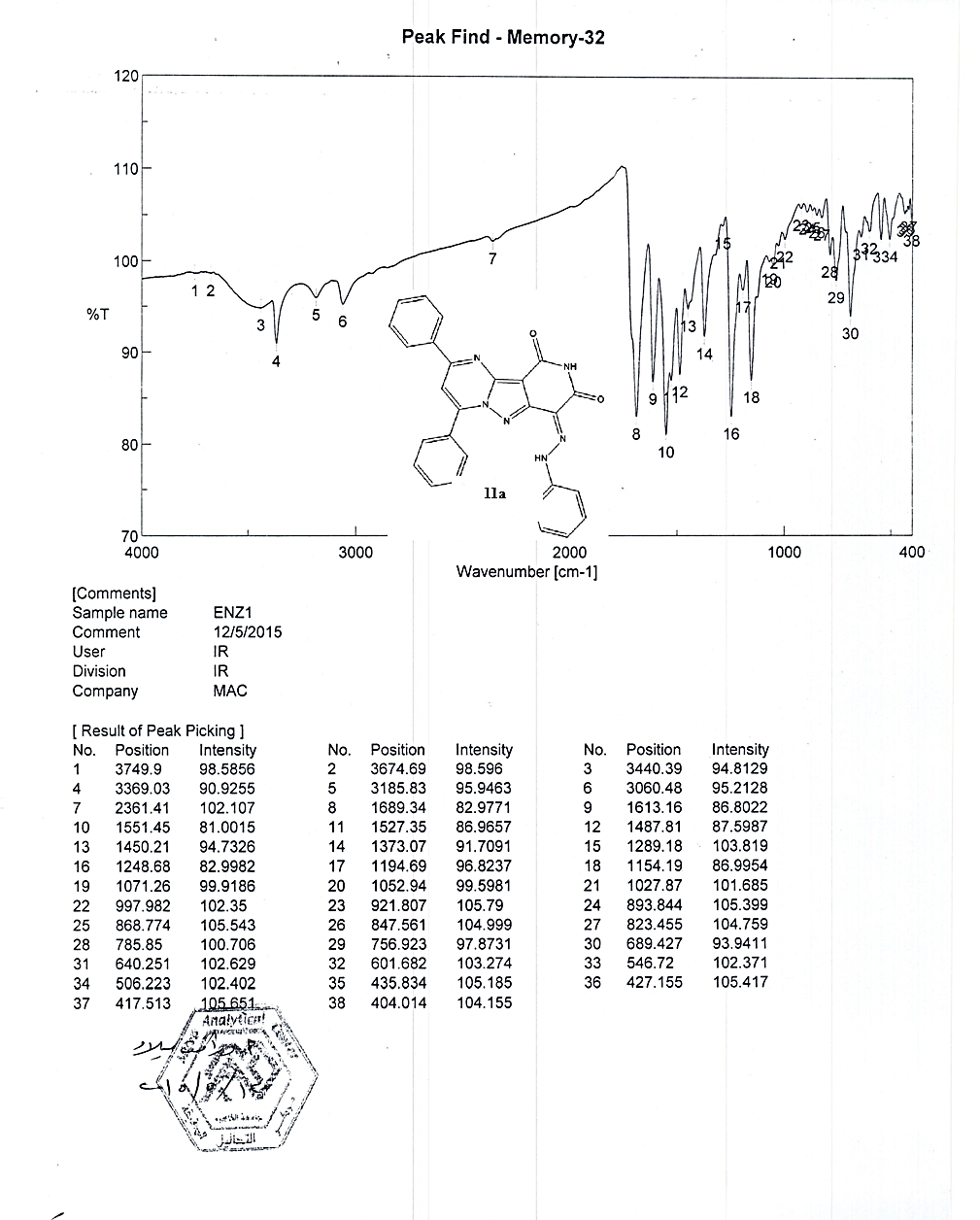


Figure (S39): IR spectrum of compound **11a**

**
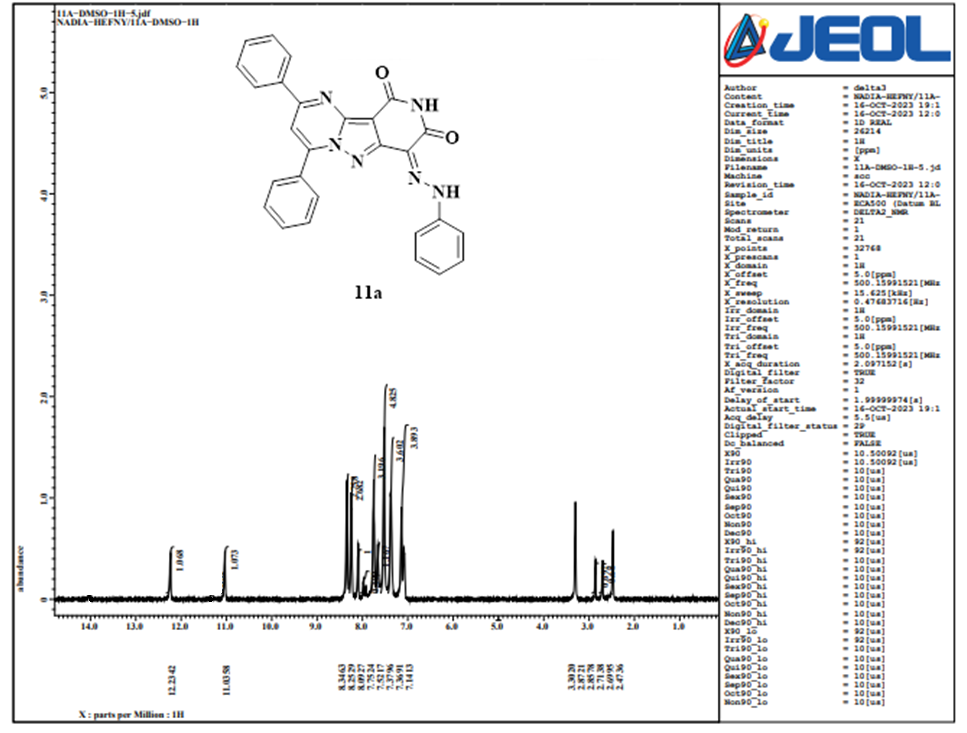
**

Figure (S40): ^1^H NMR spectrum of compound **11a**


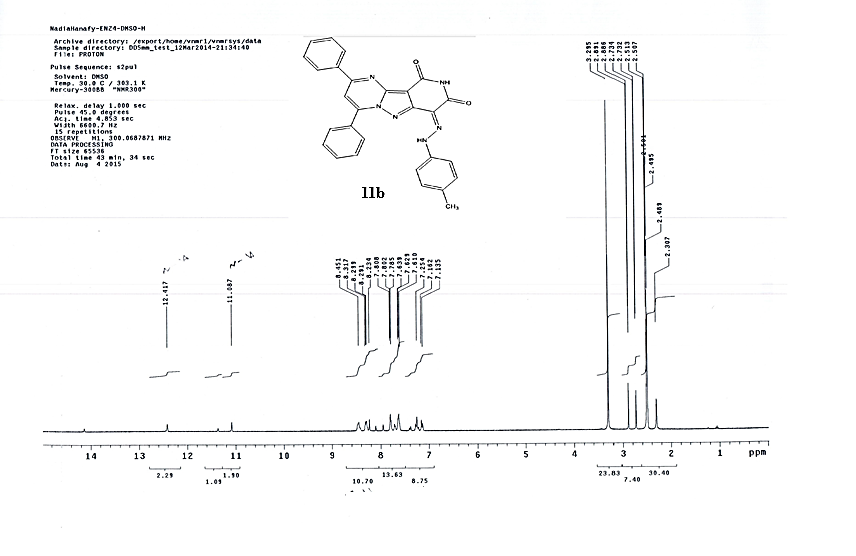


Figure (S41): ^1^H NMR spectrum of compound **11b**

Figure (S42): ^1^H NMR spectrum of compound **11c**


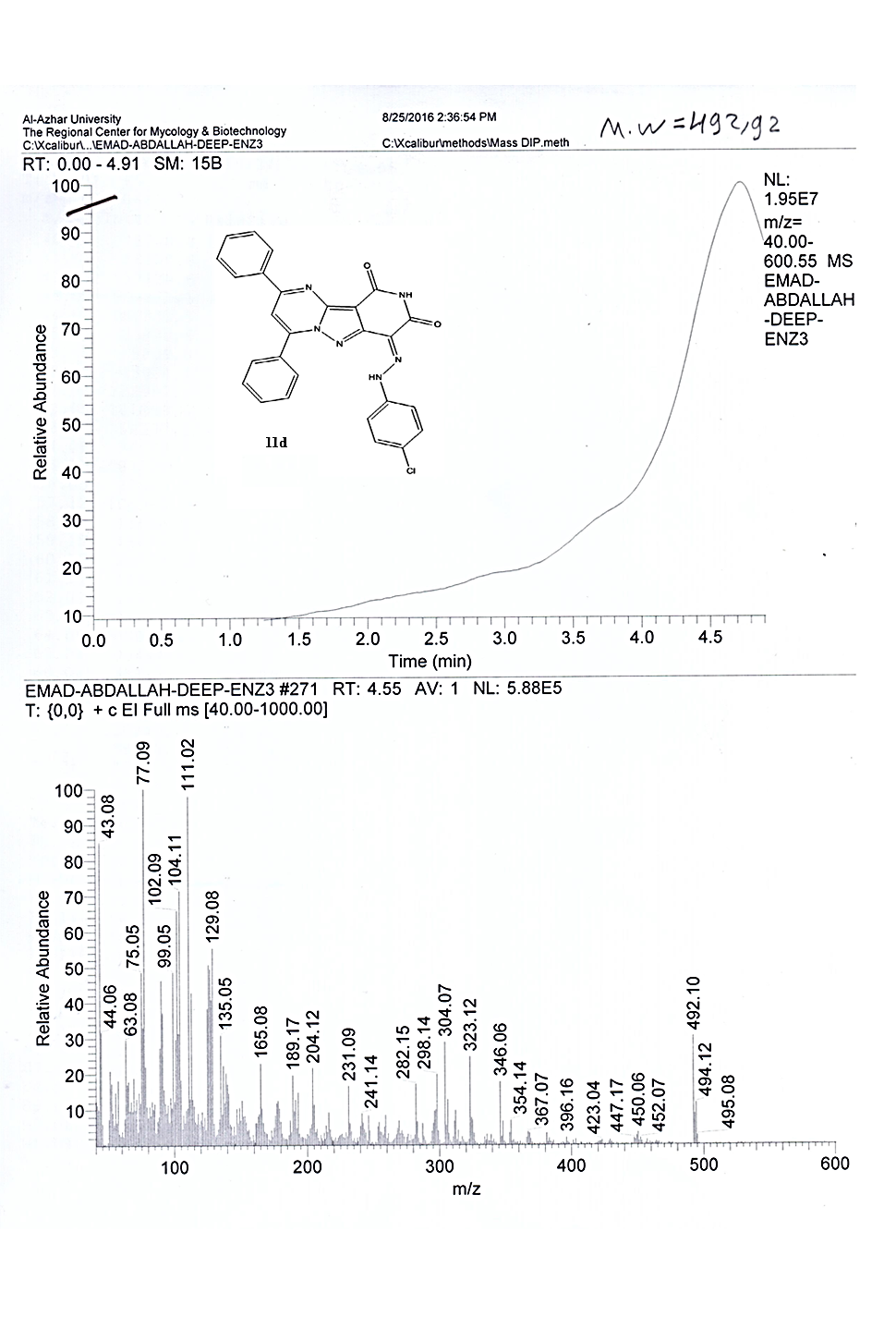


Figure (S43): Mass spectrum of compound **11d**


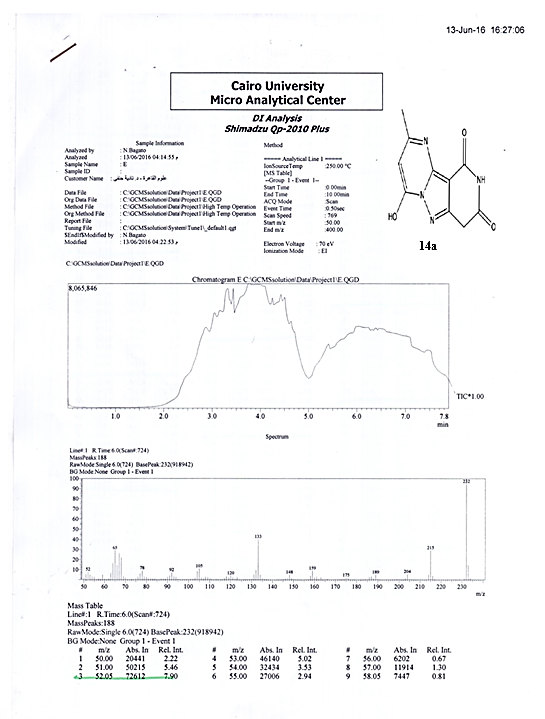


Figure (S44): Mass spectrum of compound **14a**


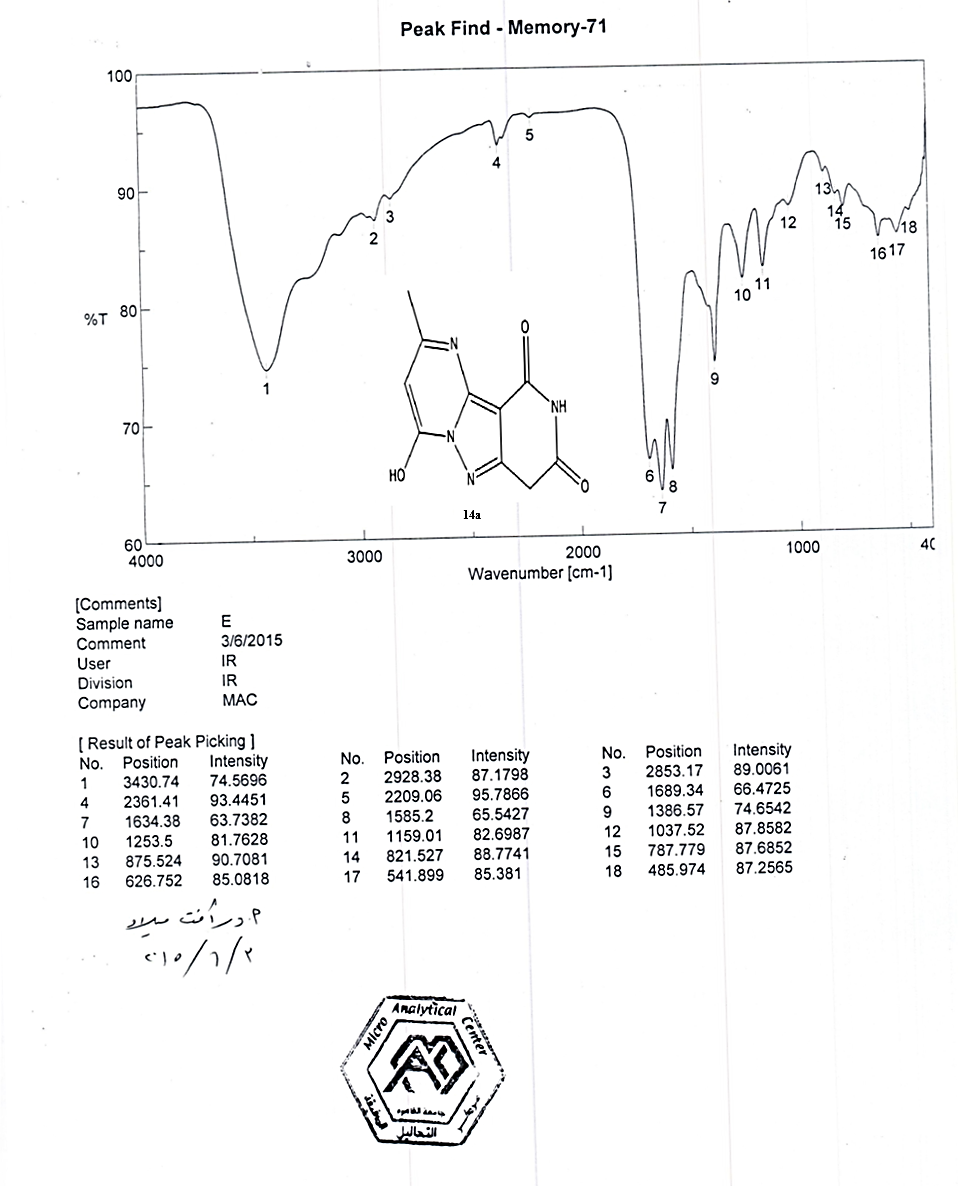


Figure (S45): IR spectrum of compound **14a**


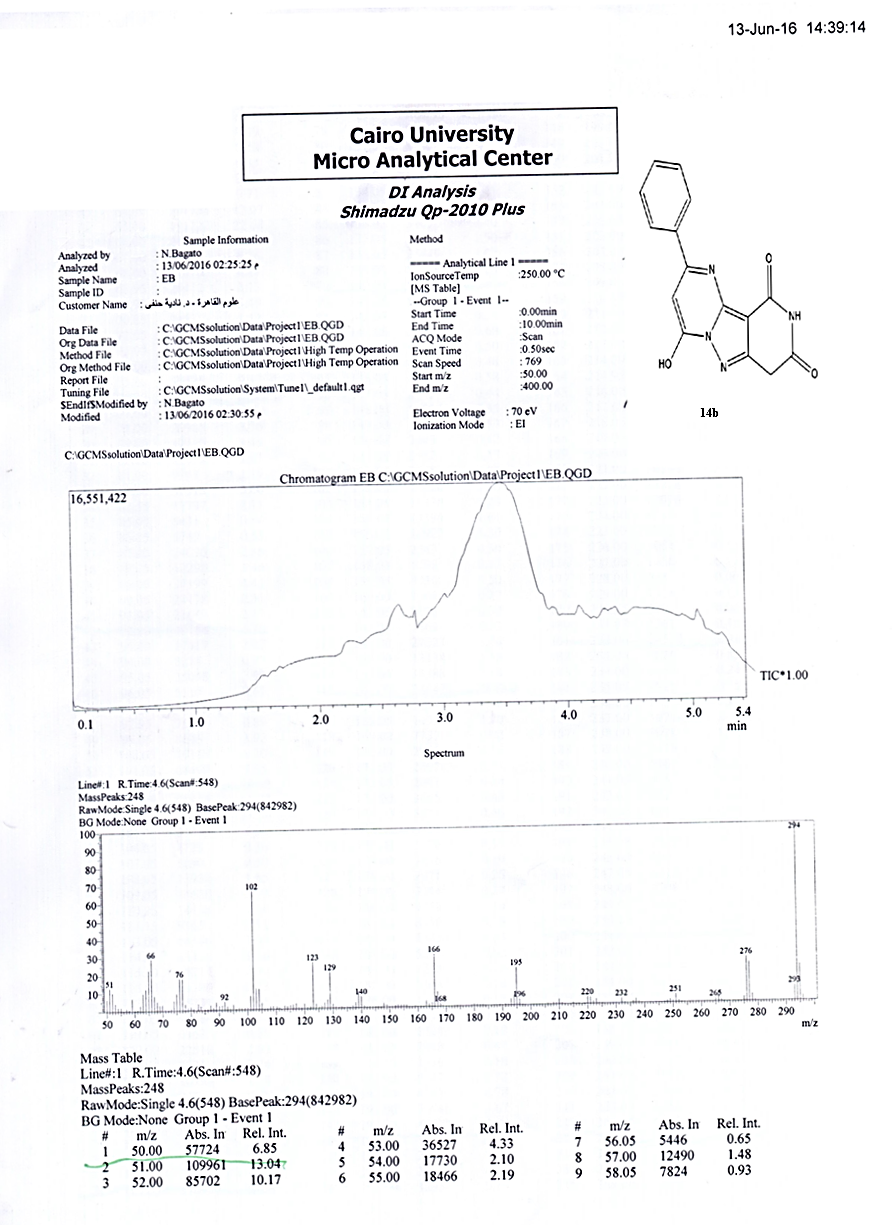


Figure (S46): Mass spectrum of compound **14b**


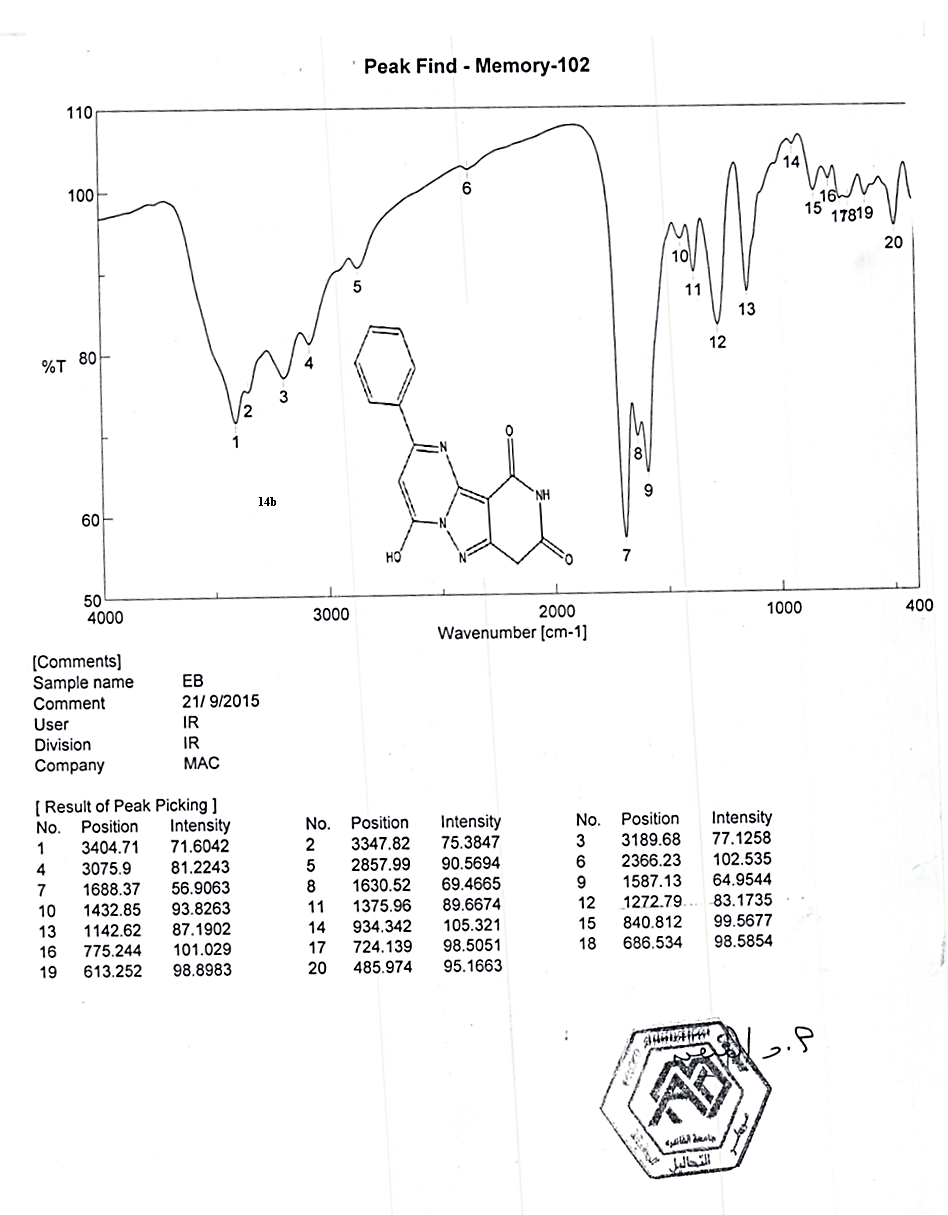


Figure (S47): IR spectrum of compound **14b**


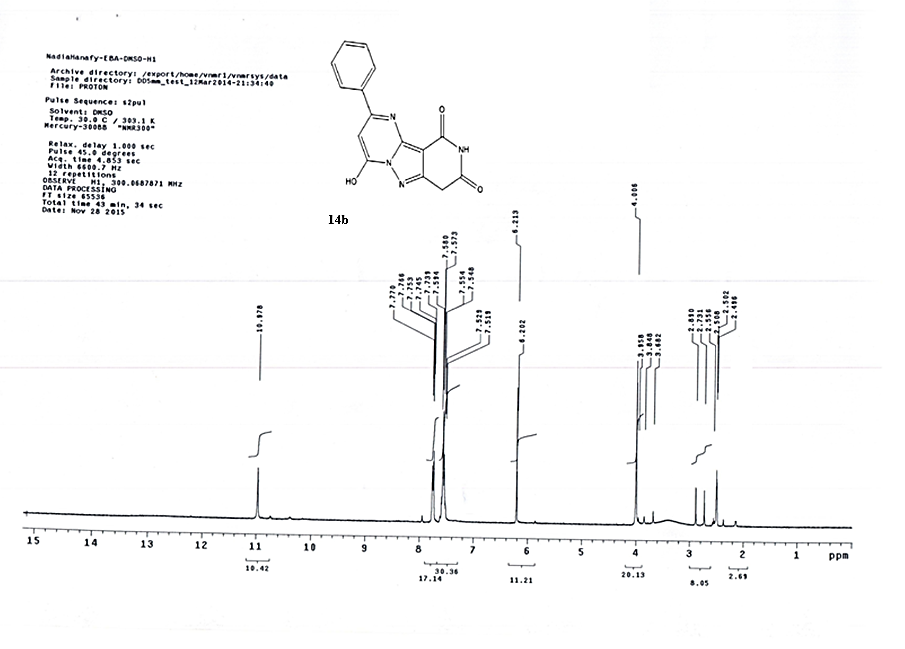


Figure (S48): ^1^H NMR spectrum of compound **14b**


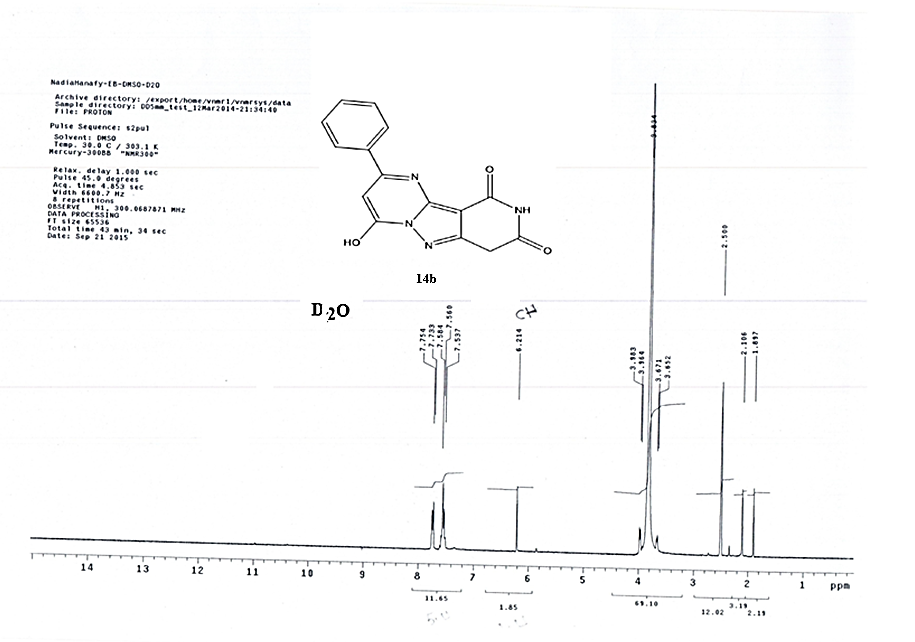


Figure (S49): ^1^H NMR spectrum of compound **14b (D_2_O)**


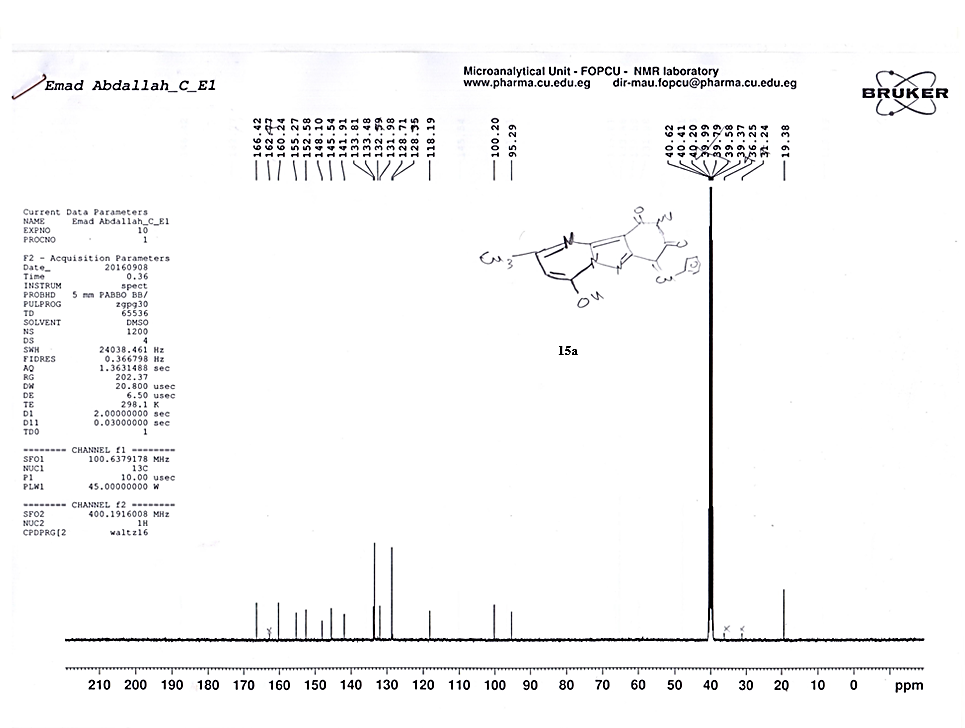


Figure (S50): ^13^C NMR spectrum of compound **15a**


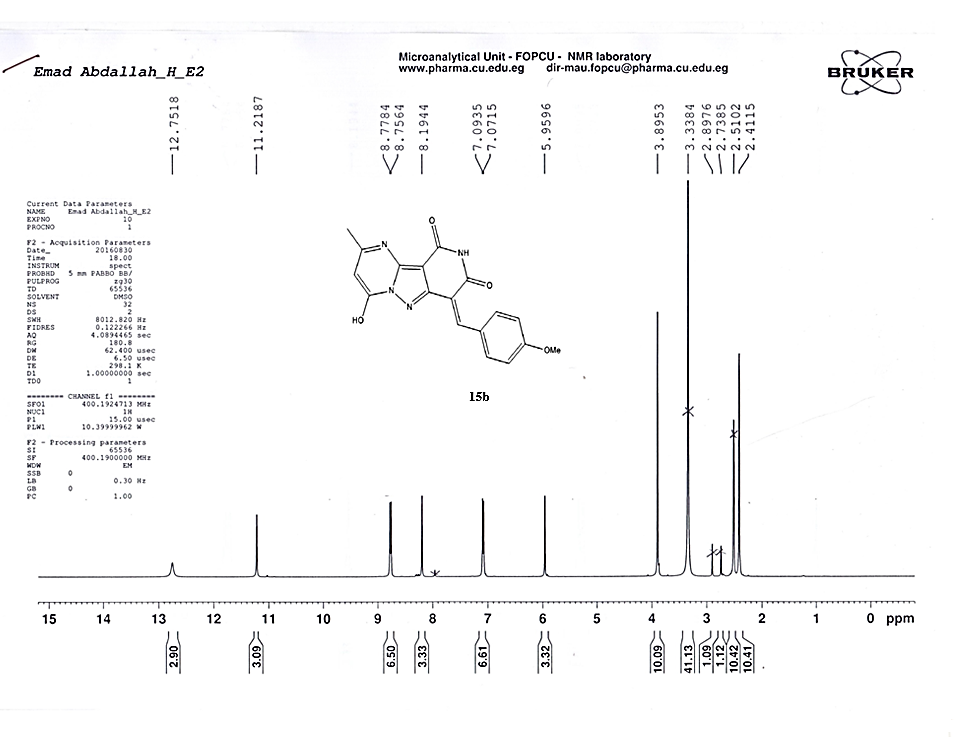


Figure (S51): ^1^H NMR spectrum of compound **15b**


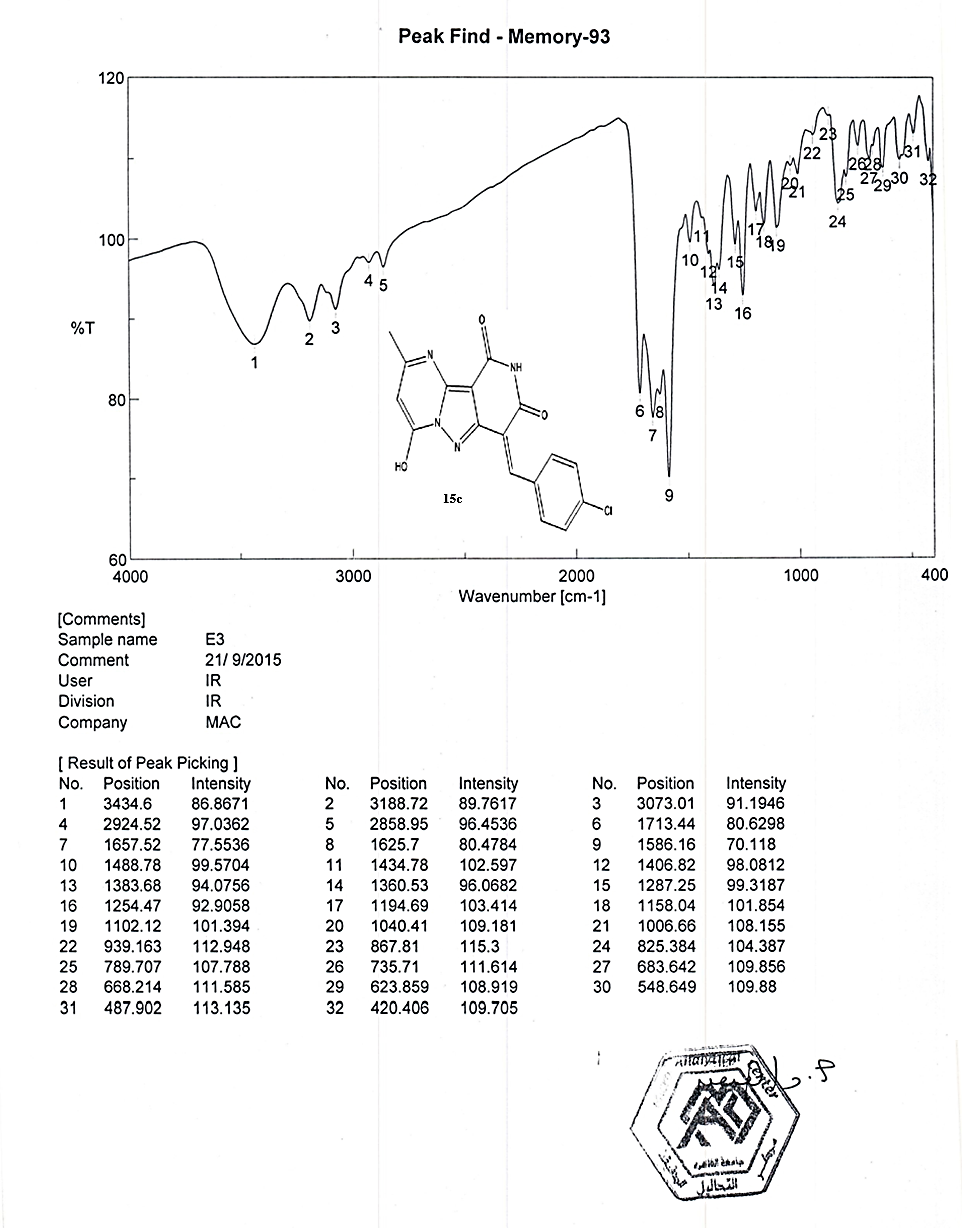


Figure (S52): IR spectrum of compound **15c**


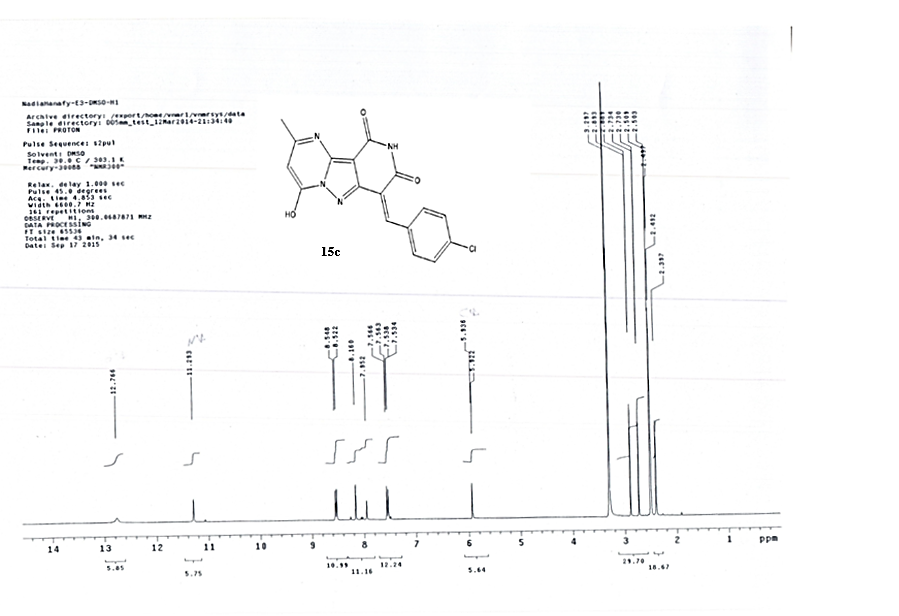


Figure (S53): ^1^H NMR spectrum of compound **15c**


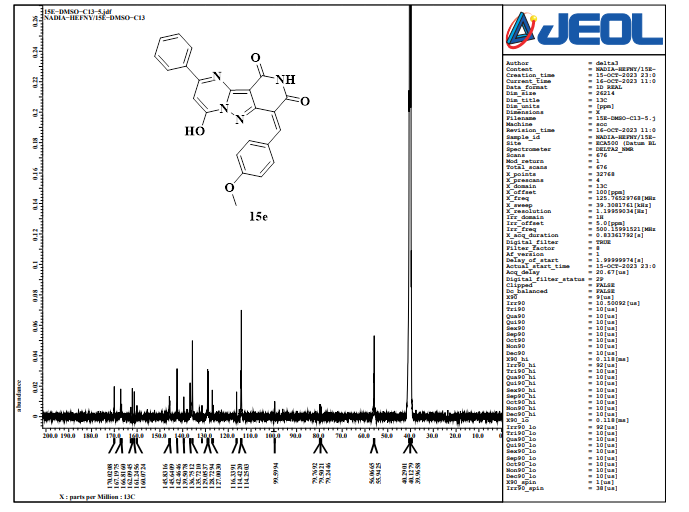


Figure (S54): ^13^C NMR spectrum of compound **15e**


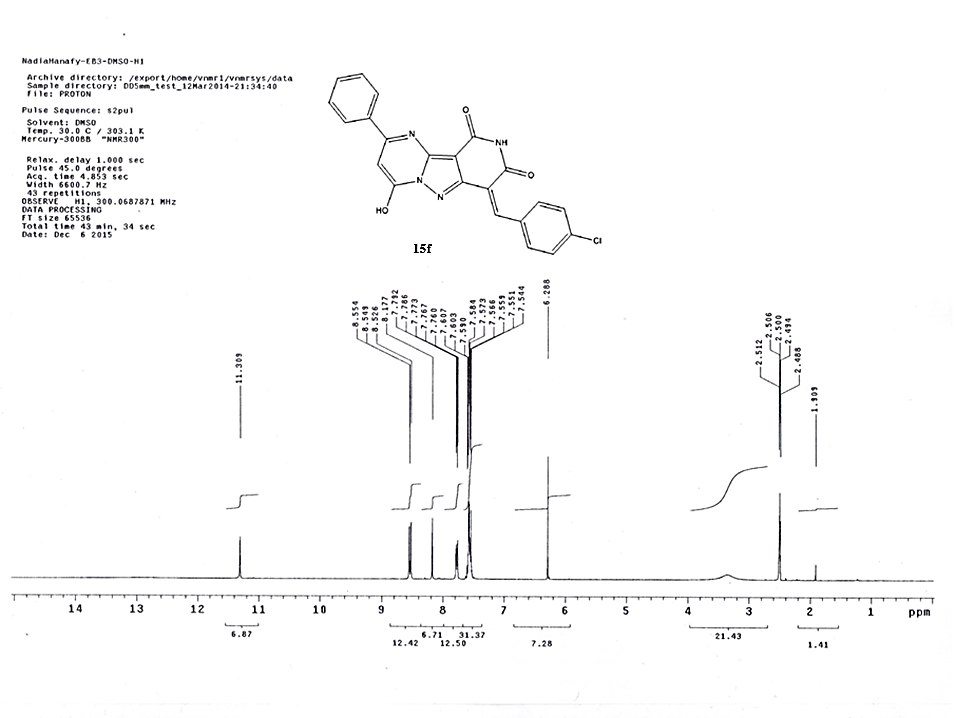


Figure (S55): ^1^H NMR spectrum of compound **15f**


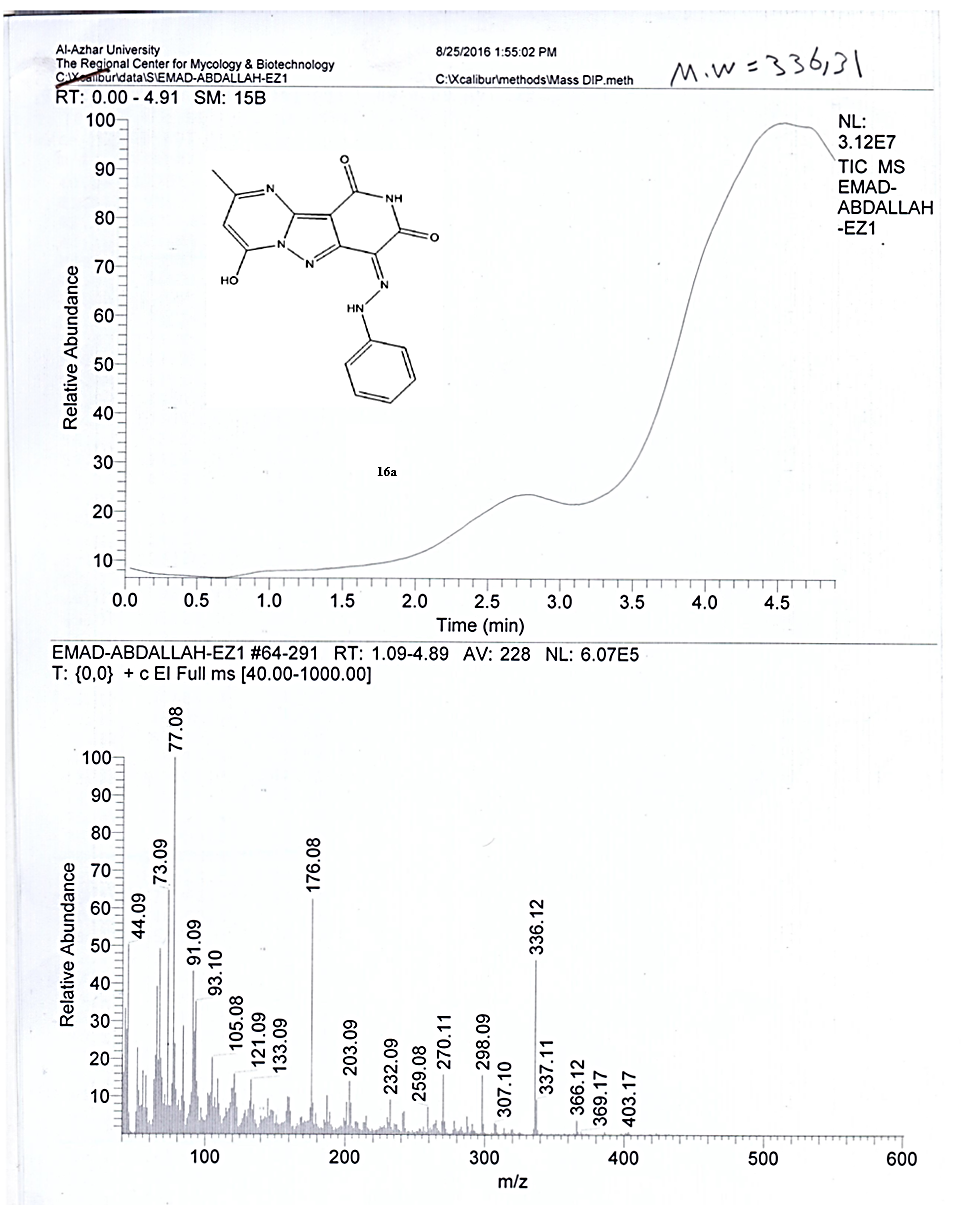


Figure (S56) Mass spectrum of compound **16a**


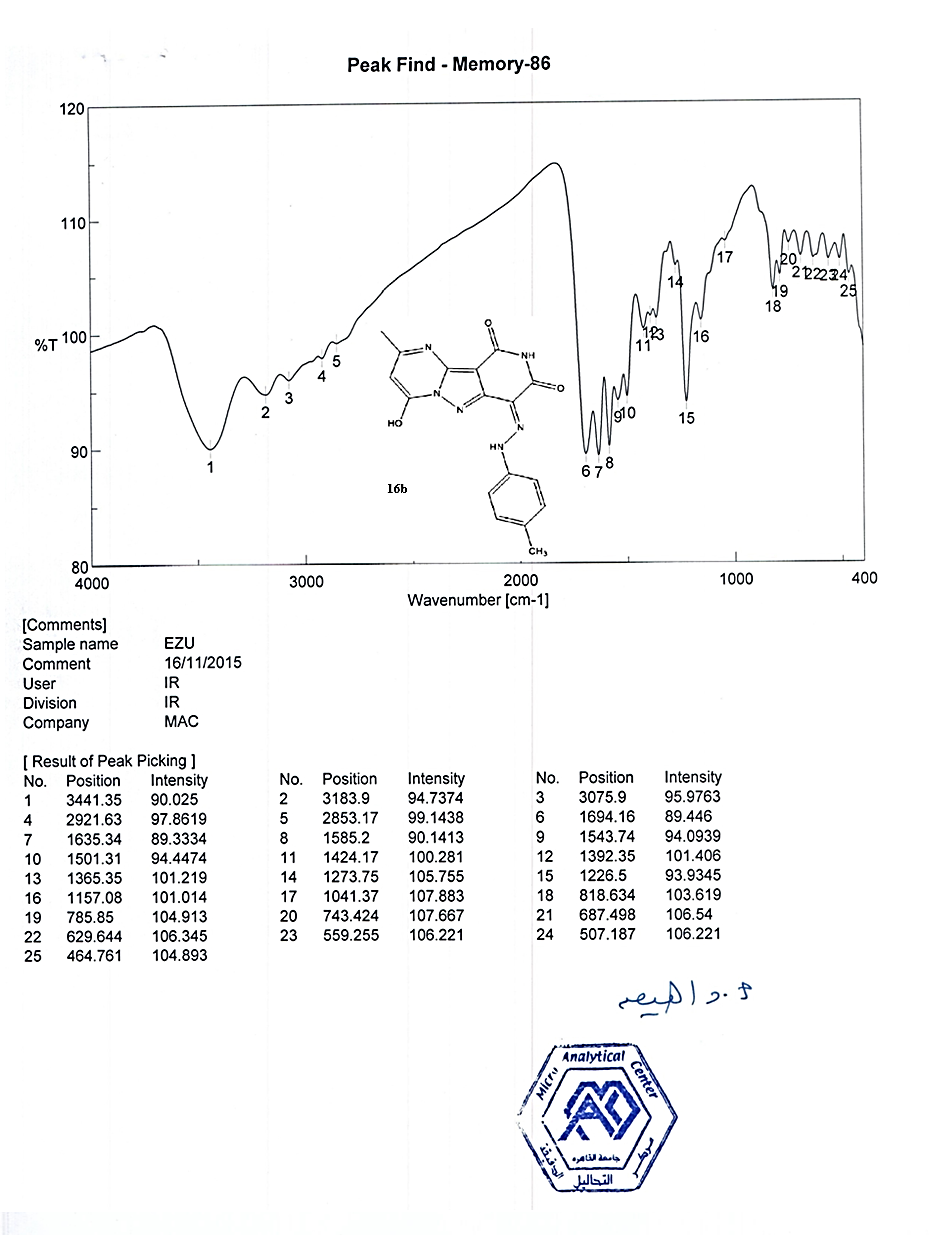


Figure (S57): IR spectrum of compound **16b**

**
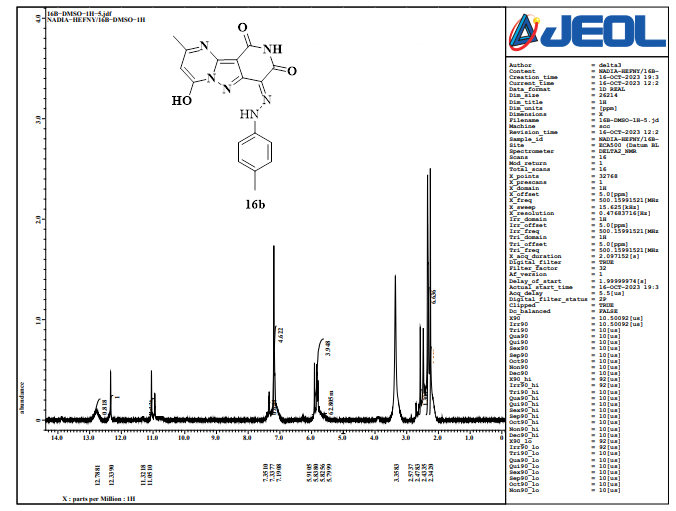
**

Figure (S58): ^1^H NMR spectrum of compound **16b**


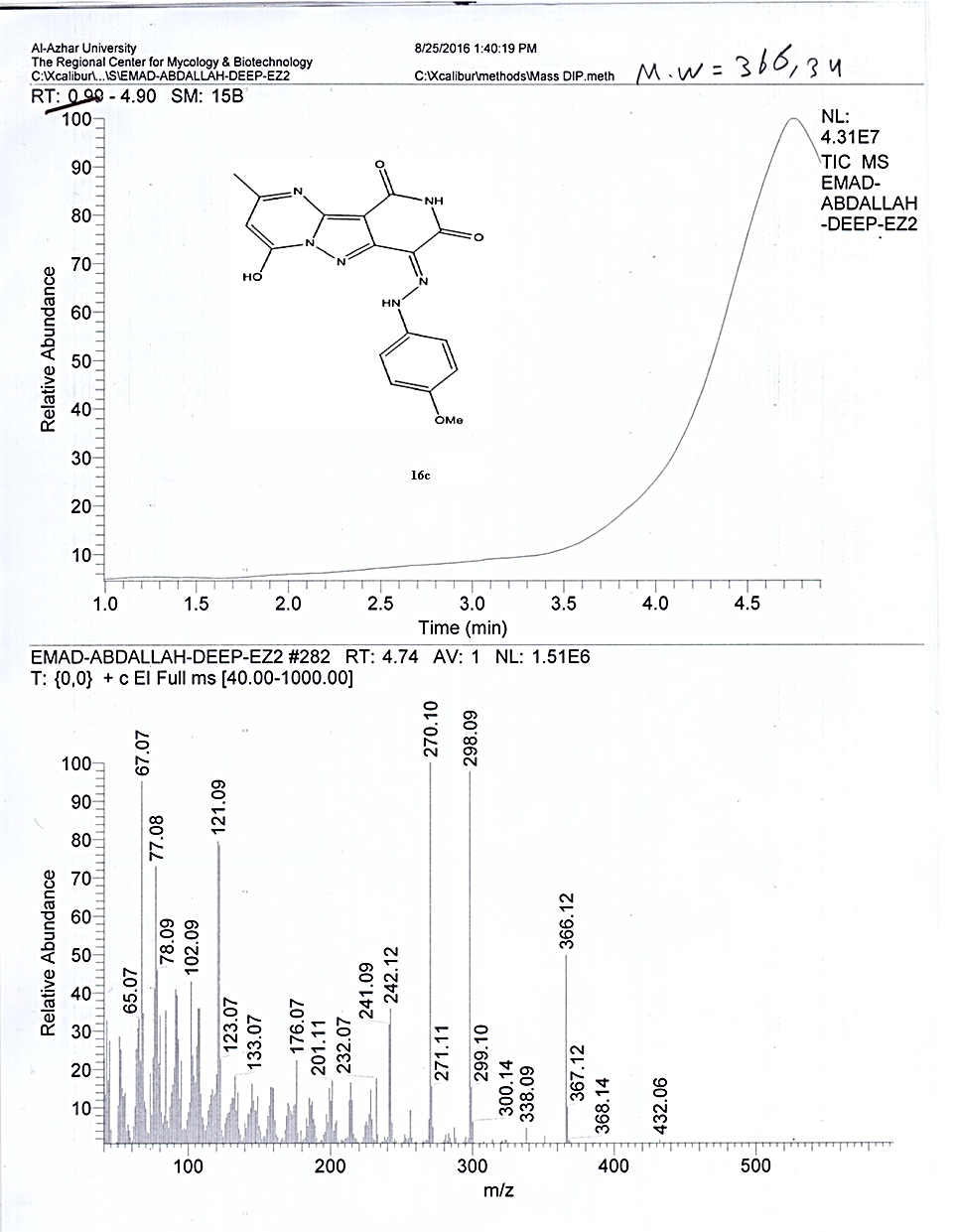


Figure (S59): Mass spectrum of compound **16c**


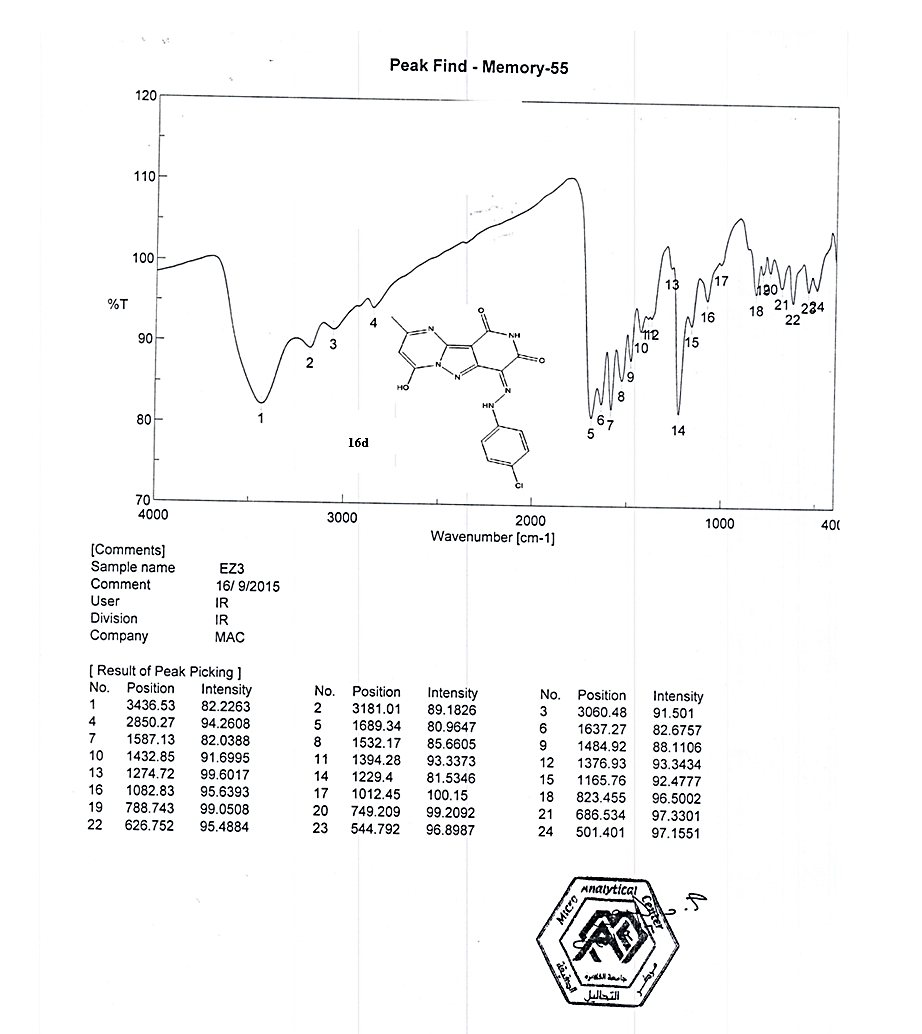


Figure (S60): IR spectrum of compound **16d**


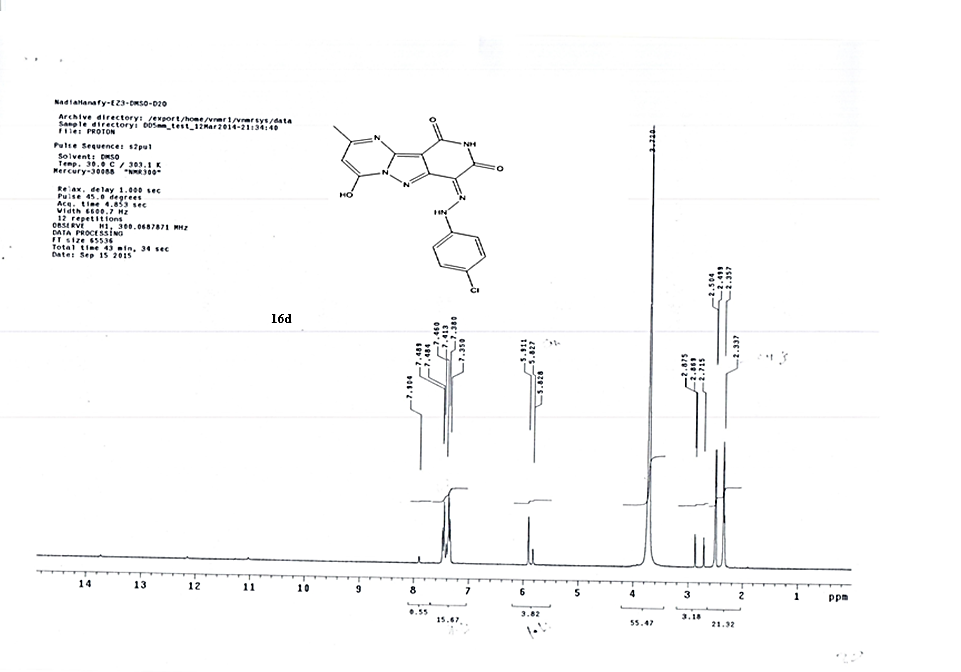


Figure (S61): ^1^H NMR spectrum of compound **16d**


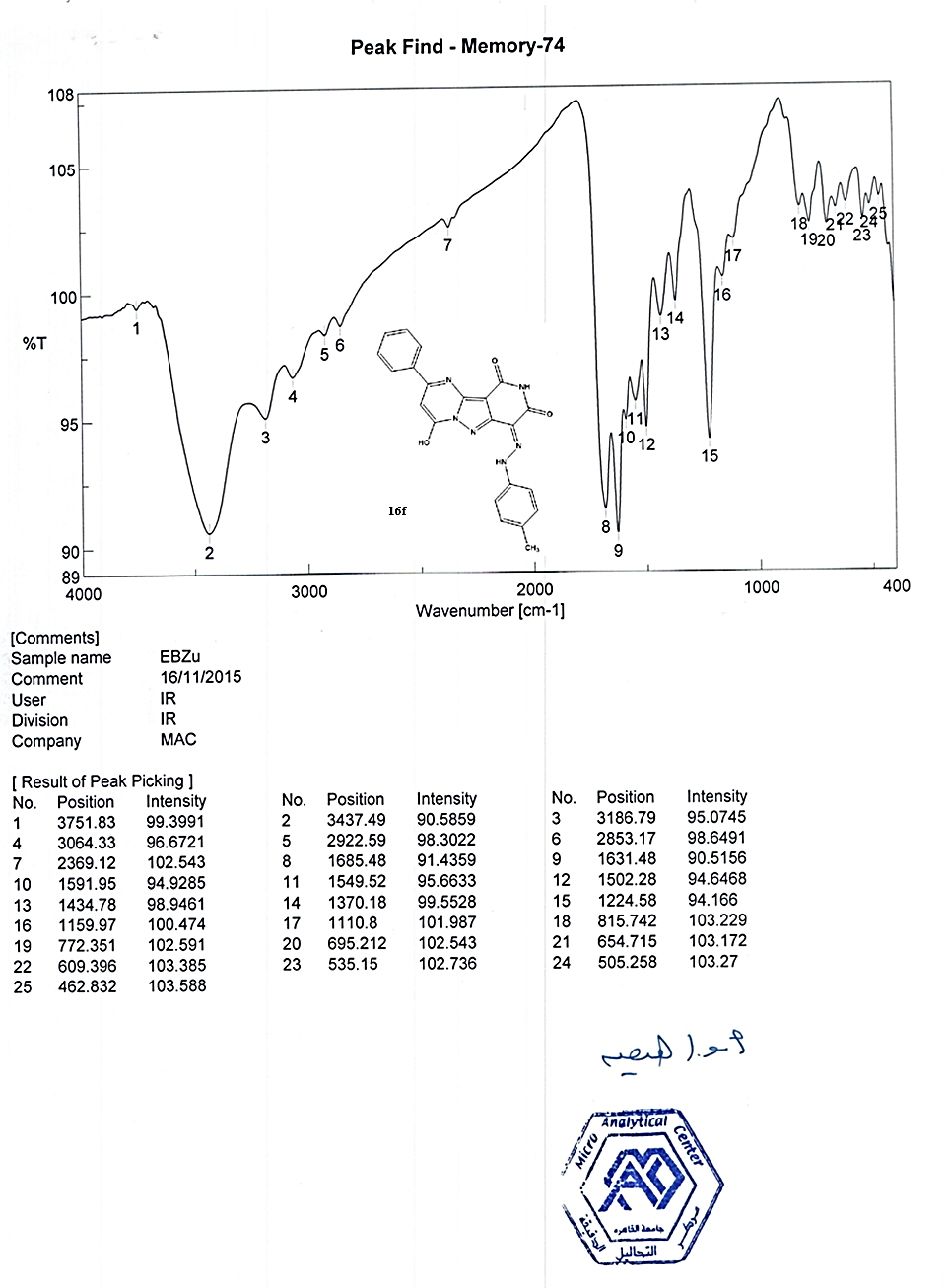


Figure (S62): IR spectrum of compound **16f**

**
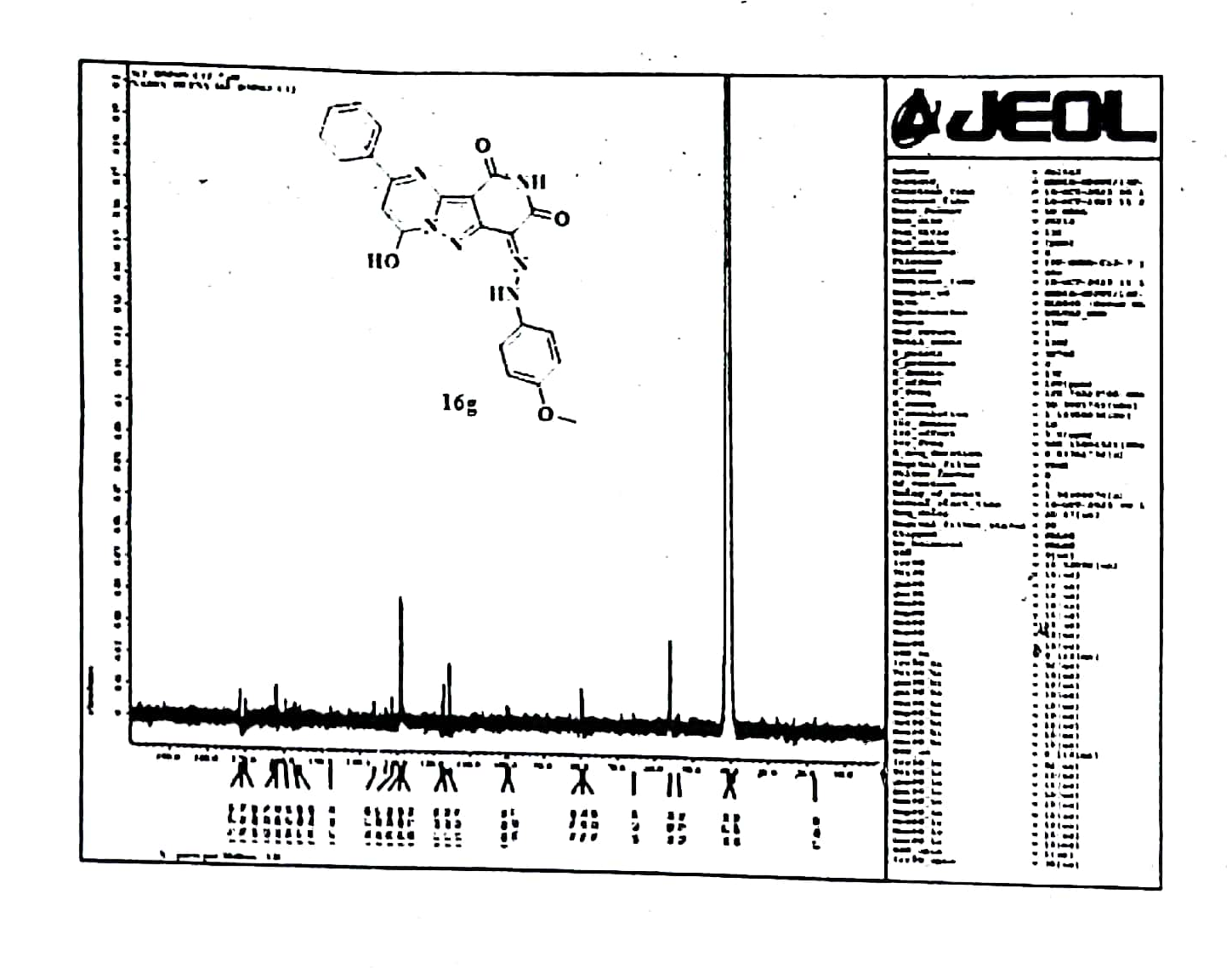
**

Figure (S63): ^13^C NMR spectrum of compound **16g**
